# Plasma-Derived sEVs from HNSCC Patients Differentially Regulate NF-κB Signaling in Macrophages Depending on the HPV Status

**DOI:** 10.3390/cancers18142219

**Published:** 2026-07-09

**Authors:** Diana Huber, Florian von Strachwitz, Linda Hofmann, Monika Pietrowska, Marta Gawin, Dapi Meng-Lin Chiang, Christiane Guder, Ramin Lotfi, Shosei Kishida, Michael W. Pfaffl, Barbara Wollenberg, Thomas K. Hoffmann, Cornelia Brunner, Marie-Nicole Theodoraki

**Affiliations:** 1 Department of Otorhinolaryngology, Head and Neck Surgery, Technical University Munich, 81675 Munich, Germany; 2 Department of Otorhinolaryngology, Head and Neck Surgery, Ulm University Medical Center, 89075 Ulm, Germany; 3 Gliwice Branch, Maria Sklodowska-Curie National Research Institute of Oncology, 44-102 Gliwice, Poland; 4 Institute of Genetics and Animal Biotechnology of the Polish Academy of Sciences, 05-552 Jastrzebiec, Poland; 5 Division of Animal Physiology and Immunology, School of Life Sciences Weihenstephan, Technical University of Munich, 85354 Freising, Germany; 6 Institute of Clinical Transfusion Medicine and Immunogenetics, German Red Cross Blood Transfusion Service Baden Wuerttemberg-Hessia, 89075 Ulm, Germany; 7 Institute for Transfusion Medicine, University Hospital Ulm, 89075 Ulm, Germany; 8 Graduate School of Medical and Dental Sciences, Kagoshima University, Kagoshima 890-8580, Japan; 9 Core Facility Immune Monitoring, Medical Faculty, Ulm University, 89075 Ulm, Germany

**Keywords:** head and neck squamous cell carcinoma, small extracellular vesicles, macrophages, cancer, HNSCC, NF-κB, human papillomavirus

## Abstract

Background: Head and neck squamous cell carcinomas (HNSCCs) are highly immunosuppressive, and tumor-associated macrophages constitute a major component of HNSCC tumor microenvironments. Here, we investigate the effects of circulating small extracellular vesicles (sEVs) from HNSCC patients on primary macrophages, to elucidate systemic sEV-mediated immune regulation in HNSCC patients. Methods: sEVs were isolated from plasma by size-exclusion chromatography. Internalization of PKH26-labeled sEVs was demonstrated, and mass spectrometry analysis was performed on HNSCC sEVs and sEV-treated macrophages. NF-κB activation was investigated by Western blot and p65 translocation assay. Downstream, mRNA and protein levels of cytokines and chemotaxis of T cells were investigated. Results: Proteomic analysis revealed time-dependent modulation of the macrophage proteome and NF-κB signaling pathway, which was confirmed by Western blot and p65 translocation assay. sEVs from HNSCC patients negative for human papillomavirus type 16 (HPV) differentially modulated NF-κB signaling and sEV uptake mechanisms compared with sEVs from HPV-positive patients and healthy donors. Conclusions: Circulating sEVs derived from HNSCC patient plasma modulate macrophage proteomic profiles and NF-κB signaling. HPV16 status was associated with differential sEV-mediated macrophage regulation, suggesting a potential role of sEVs in systemic immune modulation in HNSCC.

## Simple Summary

Head and neck squamous cell carcinomas (HNSCCs) are characterized by a strongly immunosuppressive tumor microenvironment in which tumor-associated macrophages represent a major cellular component. Small extracellular vesicles (sEVs) are one factor inducing immunosuppression, and this study investigates the effects of circulating sEVs from HNSCC patients on macrophages and their capacity to induce the immune-regulatory NF-κB signaling pathway. Interestingly, the HPV16 status of sEV donors influenced several parameters, including vesicle internalization, NF-κB activation, and CCL22 production. Overall, circulating plasma-derived sEVs from HNSCC patients substantially altered the macrophage proteome and modulated NF-κB signaling, highlighting their potential as therapeutic targets for preventing HPV16 status-dependent systemic immune regulation.

## 1. Introduction

Head and neck squamous cell carcinoma (HNSCC) comprises malignant tumors arising from the mucosal epithelium of the upper aerodigestive tract, which can develop following exposure to carcinogens or viral infections [1]. Most tumors occurring in the larynx or oral cavity are induced by tobacco and alcohol consumption, while infection with human papillomavirus (HPV), especially HPV-type 16, is typically associated with oropharyngeal tumors [2] and more infrequently with tumors in the oral cavity [3]. The global incidence of HNSCC continues to rise, with rising incidence rates in the United States and Europe largely attributed to the increasing prevalence of HPV-associated cancers [2,4], highlighting the need for new therapies targeting this group of patients. HNSCC is characterized by a highly immunosuppressive tumor microenvironment (TME) [5,6] and is heavily infiltrated by immune cells, although immune cell composition varies considerably between tumors. While infiltration with activated CD8+ or CD4+ T cells is associated with a favorable prognosis, high myeloid cell infiltration in the TME is associated with poor overall survival and therapy resistance [7]. The TME of HPV(+) HNSCC patients is generally infiltrated with higher numbers of anti-tumor immune cells, classifying these tumors as immunologically ‘hot’. In comparison, an HPV(−) HNSCC is characterized as immunologically ‘cold’ with less infiltration of anti-tumor lymphocytes but higher infiltration of myeloid cells. Tumor-associated macrophages (TAMs) were shown to be involved in multiple stages of tumorigenesis, including promotion of tumor growth, angiogenesis, immune evasion, and metastasis formation [8]. In HNSCCs, TAMs were shown to produce increased levels of EGF, promoting tumor growth [9], and to contribute to immunosuppression, for example, by PD-L1 expression, which inhibits T cell functions and induces apoptosis in natural killer cells [10,11].

An important mediator of communication within the TME is small extracellular vesicles (sEVs). sEVs are lipid bilayer-enclosed particles released by every cell type, which carry molecules derived from their cells of origin and circulate throughout the body via body fluids [12]. They mediate intercellular communication either by transferring their cargo to recipient cells through vesicular fusion or by interacting with cell surface receptors. Within the TME, sEVs contribute to communication between tumor cells and immune cells, thereby mediating immunosuppression [13].

In HNSCCs, circulating sEVs derived from the blood plasma of HNSCC patients contribute to systemic immunosuppression by inhibiting B cell functions, inducing apoptosis in CD8+ T cells, promoting the transformation of CD4+ T cells into regulatory T cells (Treg), and supporting their immunosuppressive abilities [14,15]. Furthermore, sEVs derived from HNSCC cells also drive tumor-promoting functions of TAMs through STAT3 activation [16] or via CD73(+) sEVs, which induce NF-κB activation and subsequent immunosuppression through the production of immunosuppressive cytokines [17]. The NF-κB family of transcription factors includes p65 (RelA), RelB, c-Rel, p105/p50 (NF-κB1), and p100/52 (NF-κB2) and regulates several important functions, including cell survival, proliferation, and immune cell functions [18]. Moreover, the NF-κB pathway has been shown to be constitutively activated in HNSCC tissues and cell lines, contributing to tumor progression [19,20].

While NF-κB activation by sEVs derived from HNSCC cell lines has been widely reported, the effects of circulating plasma-derived sEVs from HNSCC patients on macrophages remain poorly understood. Therefore, this study investigates the impact of patient-derived circulating sEVs on primary macrophages to better characterize their potential role in systemic macrophage modulation and the induction of TAM-like characteristics, thereby more closely reflecting the in vivo situation. During the analysis of patient-derived sEV effects, differences associated with HPV status were observed. Since HPV status is associated with distinct biological features in HNSCCs, these observations prompted an exploratory analysis of potential differences in systemic immune modulation between HPV(+) and HPV(−) HNSCCs. These findings may provide insights into disease-associated mechanisms of macrophage-mediated immune regulation and highlight potential HPV status-associated differences in systemic immune modulation in HNSCCs.

## 2. Materials and Methods

### 2.1. Plasma Sample Collection (In Vivo Model)

Small extracellular vesicles were isolated from the blood plasma of healthy donors (HD) or newly diagnosed, treatment naïve HNSCC patients, with every donor providing informed and written consent (Votum #90/15). HNSCC patients with advanced tumor stages and healthy donors with higher age, matching the HNSCC cohort, were preferred. Venous blood was collected in citrate tubes (Sarstedt, Nümbrecht, Germany) at the Ulm University Medical Center, Department of Otorhinolaryngology, and prepared by centrifugation at room temperature (RT) at 1000× *g* and at 2500× *g* for 10 min each before plasma was frozen in aliquots at −20 °C.

### 2.2. Cell Lines (In Vitro Model)

The mouth ordinary epithelium (MOE) cell line 1a, established by Kishida et al. [21], served as a non-cancer control. The HPV-negative squamous cell carcinoma cell line UD-SCC-1 was derived from the oropharynx and established at the University of Düsseldorf. UPCI-SCC-90 cells were established at the University of Pittsburgh, derived from HPV-positive high-stage squamous cell carcinoma from the base of the tongue.

All cell lines were cultivated in DMEM (gibco, Thermo Fisher Scientific, Waltham, MA, USA) without phenol-red, supplemented with 10% (*v*/*v*) exosome-depleted fetal bovine serum (FBS) (A2720801, Thermo Fisher Scientific, Waltham, MA, USA), 1% (*v*/*v*) ZellShield (Minerva Biolabs GmbH, Berlin, Germany), and 1% (*v*/*v*) non-essential amino acids (gibco, Thermo Fisher Scientific) at 37 °C with 5% CO_2_. All cultures were authenticated by short tandem repeat analysis just prior to performance of the described experiments. For sEV isolation from supernatants, cells were incubated for 72 h at 37 °C without medium exchange.

### 2.3. Isolation and Characterization of sEVs

sEVs were either isolated from blood plasma or from cell culture supernatant from UD-SCC-1, UPCI-SCC90, and MOE1a cells by sequential centrifugation, ultrafiltration, and size exclusion chromatography, as described before [22,23]. sEV concentration was determined using the Pierce BCA Protein Assay (23225, Thermo Fisher Scientific), and the samples were concentrated to the required concentration for subsequent experiments using Amicon Ultra centrifugal filters with a molecular weight cut-off of 100 kDa (Merck Millipore).

Nanoparticle tracking analysis, Western blots, and transmission electron microscopy were performed to confirm successful isolation of sEVs, as suggested by the Minimal information for studies of extracellular vesicles (MISEV) guidelines [24]. These methods were employed routinely in our lab, as described in more detail previously [25]. For cryo-TEM analysis, 2 µL of the sample was applied to a holey carbon-coated copper grid (Ted Pella, Inc.) and blotted with Whatman No. 1 filter paper to remove excess liquid. The grid was immediately vitrified by rapid plunge-freezing into liquid ethane cooled to −178 °C using a Leica EM GP plunger (Leica Microsystems GmbH). Vitrified grids were transferred under liquid nitrogen to a Talos transmission electron microscope (FEI) equipped with a Gatan 626 cryo-holder. Imaging was performed at an accelerating voltage of 200 kV under low-dose conditions (~20 e^−^/Å^2^) to minimize radiation damage. Micrographs were acquired using a CETA CMOS camera (Thermo Fisher Scientific, Inc.) while maintaining the specimen at cryogenic temperatures throughout data collection. Isolated sEVs can be assigned to EV-TRACK ID: EV200068.

### 2.4. Isolation and Cultivation of Macrophages and T Cells

Immune cells used for experiments were isolated from Buffy Coats from healthy donors, provided by the German Red Cross (DRK Ulm), by centrifugation at RT at 800× *g* for 10 min in pre-filled Leucosep tubes (Greiner Bio-One, Kremsmünster, Austria).

Immune cells were isolated using the CD14 MicroBeads for monocytes, the CD8+ T Cell Isolation Kit for CD8+ T cells, and the CD4+ T Cell Isolation Kit, followed by biotinylated anti-CD39 antibodies and anti-Biotin MicroBeads for CD4+ CD39+ regulatory T cells, all from Miltenyi Biotec (Bergisch-Gladbach, Germany) according to the manufacturer’s instructions. The cells were used for experiments if the purity was at least 90%. Monocytes were seeded into 24-well plates with a cell number of 500,000 cells per well in IMDM (gibco, Thermo Fisher Scientific) supplemented with 10% exosome-depleted FBS, 1% ZellShield, and 1000 U/mL GM-CSF (Miltenyi Biotec) for 6 days, with 0.5 mL of medium being exchanged with fresh IMDM containing 2000 U/mL GM-CSF on day 3. On day 6, 15 µg of sEVs were added to one well containing approximately 2 × 10^6^ cells in 1 mL of medium.

### 2.5. PKH-Labeling of sEVs and Uptake Experiments

The required amount of sEVs concentrated to 100 µL was mixed with 100 µL Diluent C provided in the PKH26 cell linker kit (Sigma-Aldrich, St. Louis, MO, USA), incubated with 1 µL PKH26 diluted in Diluent C for 10 min, and washed with PBS using the Amicon Ultra Centrifugal Filters with a 100 kDa cut off. As a negative control, the same procedure was performed using 100 µL PBS instead of sEVs.

For microscopy images, macrophages were incubated with PKH-labeled sEVs for several time points (30 min, 1 h, 2 h, 4 h, 16 h, and 24 h). Then, the cells were washed with pre-warmed PBS, fixed with 4% paraformaldehyde (PFA) for 20 min, and permeabilized with 0.01% Triton-X in PBS for 3 min before incubation with ActinGreen and NucBlue ready probes (both Invitrogen) for 30 min. The cells were mounted in Gold antifade mounting medium (Invitrogen) and imaged the next day using the Zeiss Axio Observer microscope. For flow cytometry analysis, the cells were detached after incubation with sEVs using TrypLE (gibco, Thermo Fisher Scientific) and washed three times with PBS before measuring the fluorescence intensity at 525 nm using the Gallios flow cytometer from Beckmann Coulter. For inhibition experiments, sEV uptake was inhibited by adding 20 mg/mL dynasore (324410, Sigma Aldrich) and 100 µmol/L genistein (G6649-5MG, Sigma Aldrich) 15 min prior to adding sEVs.

### 2.6. LC-MS/MS Analysis of Macrophages and sEVs

Macrophages were incubated with PBS or 15 µg/mL sEVs for 6 h or 24 h, then three wells per condition, containing around 2 × 10^6^ cells per well, were lysed. sEVs used for the incubation experiments were also lysed, and all samples were prepared by the filter-aided sample preparation (FASP) method established by Wisniewski et al. [26], modified as described previously with trypsin digestion only [27]. The samples were separated into two strong anion exchanger fractions by elution at pH 5 and pH 2 and analyzed separately. The employed nano-LC-MS/MS system comprised a UltiMate 3000 RSLCnano liquid chromatograph connected to a Q Exactive Plus mass spectrometer (both from Thermo Scientific). Peptides were separated on a reverse phase Acclaim PepMap 100 nanoViper C18 column (particle size: 2 µm, diameter: 75 µm, length: 500 mm) using an acetonitrile gradient (phase A: 0.1% formic acid in water; phase B: 80% ACN, 0.1% formic acid), at 30 °C and flow rate of 300 nL/min (for 200 min). The gradient was as follows: 3–8% B for 7 min, 8–35% B for 130 min, 35–60% B for 20 min, 80% B for 20 min, and 3% B for 20 min. The system was operated in data-dependent analysis (DDA) mode with survey scans acquired at a resolution of 70,000 at *m*/*z* 200 in MS mode, and 17,500 at *m*/*z* 200 in MS2 mode. Spectra were recorded in the scanning range of 300–2000 *m*/*z* in the positive ion mode. Higher energy collisional dissociation ion fragmentation was performed with normalized collision energies set to 25. Protein identification was performed using the Swiss-Prot human database: UniProtKB Release 2023_03, consisting of 248,842,690 entries (UniProtKB/Swiss-Prot: 569,793 entries and UniProtKB/TrEMBL: 248,272,897 entries), with a precision tolerance of 10 ppm for peptide masses and 0.02 Da for fragment ion masses. Raw data were imported into Proteome Discoverer v.1.4 (Thermo Scientific) <Thermo raw files> for protein identification and quantification. The Sequest engine was used for database searches, and proteins where at least two peptides per protein were found with an FDR threshold of 0.01 were considered as positively identified. Abundances were estimated in Proteome Discoverer using the Precursor Ions Area detector node. The mass spectrometry proteomics data have been deposited to the ProteomeXchange Consortium via the PRIDE [28] partner repository with the dataset identifier PXD069623 and 10.6019/PXD069623.

### 2.7. NF-κB Activation Assays

Macrophages were incubated with 15 µg/mL sEVs, 100 µL PBS, or 80 HAU Sendai virus (SV) (10100774, Charles River Laboratories, Wilmington, MA, USA) as a positive control, for 2 h, 4 h, or 24 h. They were also co-incubated with sEVs and NF-κB inhibitors: 10 µM Bay 11-7082 (196870, Sigma Aldrich, Darmstadt, Germany), 250 µg/mL Caffeic acid phenethyl ester (CAPE) (211200, Sigma Aldrich), and 180 µg/mL curcumin (C7727, Sigma Aldrich) for 2 h, 4 h, and 24 h. Concentrations of NF-κB inhibitors are adopted from Kötting et al. [29]. Multiple inhibitors with different pharmacological mechanisms were used to confirm sEV-induced NF-κB activation and minimize off-target artifacts.

To investigate the effects of different sEVs on NF-κB activation, macrophages were incubated with sEVs for 3 h, because in the previous assay, similar NF-κB activation was observed after 2 h and 4 h. Finally, macrophages were incubated with the sEV-uptake inhibitors dynasore and genistein for 15 min prior to adding sEVs for 3 h.

NF-κB activation was either assessed directly by measuring nuclear levels of p65 or by measuring the ratio of pIκB to IκB by Western blot analysis using anti-IκBα (1:1000, 9242, Cell Signaling Technology, Danvers, MA, USA) or anti-pIκBα (1:500, MA5-15224, Invitrogen). As a loading control, α-Tubulin was measured, and the ratio of pIκB/IκB was calculated. Measuring NF-κB activation through pIκB and IκB allows measuring canonical as well as non-canonical NF-κB signaling, while measuring p65 nuclear translocation reflects canonical NF-κB signaling.

To measure p65 nuclear translocation, nuclear extracts were prepared using the Nuclear Extract Kit (40410, Active Motive, Carlsbad, CA, USA) according to the manufacturer’s instructions. The total protein concentration of nuclear extracts was measured using the ProStainTM Protein Quantification Kit (15001, Active Motif), and the concentration of p65 in the nuclear extracts was determined using the TransAM^®^ NF-κB p65 Activation Assay (40096, Active Motif), according to the manufacturer’s instructions.

### 2.8. Measurement of Cytokines

The supernatant of macrophages was collected after 24 h of incubation with sEVs, PBS, or SV, centrifuged for 10 min at 12,000× *g,* and stored at −80 °C. Cytokine concentration of interleukin (IL)-1β, IL-6, IL-10, CC-chemokine ligand 2 (CCL2), C-X-C motif chemokine (CXCL) 10, and tumor necrosis factor (TNF) was determined in the supernatants by the Multiplex Assay from Merck Millipore using the Luminex Technology according to the manufacturer’s instructions. Furthermore, production of CCL5 and CCL22 was measured by ELISA to investigate HPV-dependent differences. EIA plates were coated with anti-capture antibody (CCL22: MAB336, CCL5: MAB678, R&D Systems) and incubated overnight. The plate was blocked with 2% BSA in PBS for 1 h before adding the cell culture supernatant for 2 h. Biotinylated detection antibody (CCL22: BAF336, CCL5: BAF278, R&D Systems) was added, followed by HRP-conjugated Streptavidin (N100, Thermo Fisher) and TMB substrate (N301, Thermo Fisher), with washing in between. TMB was incubated until a blue color developed, then the reaction was stopped by adding 2% H_2_SO_4_, and the absorbance at 450 nm was measured using the Tecan spectrophotometer.

mRNA expression of several cytokines was determined by RT-qPCR. Macrophages were incubated with sEVs for 24 h and lysed with RLT buffer from the RNeasy mini-Kit (Qiagen, Hilden, Germany). Total RNA was isolated according to the manufacturer’s instructions, and the isolated RNA was quantified at 260 nm using the Tecan plate reader. mRNA purity was tested by OD260/280 and OD260/230, and mRNA was transformed into cDNA using the QuantiNova Reverse Transcription Kit (Qiagen) according to the manufacturer’s instructions. RT-qPCR was performed using the QuantiNova SYBR Green PCR Kit (Qiagen), as described previously [30]. The primers were obtained from Biomers: *B2M* (forward TTC TGG CCT GGA GGC TAT, reverse TCA GGA AAT TTG ACT TTC CAT), *CCL2* (forward AAG ATC TCA GTG CAG AGG CTC G, reverse TTG CTT GTC CAG GTG GTC CAT), *CXCL10* (forward GGT GAG AAG AGA TGT CTG AAT CC, reverse GTC CAT CCT TGG AAG CAC TGC A), *IL6* (forward CAA TCT GGA TTC AAT GAG GAG AC3′, reverse 5′CTC TGG CTT GTT CCT CAC TAC TC), *TNF* (forward GCA GAG GAC CAG CTA AGA GG, reverse GAG CCG TGG GTC AGT ATG TG) and *IFNB* (qHsaCED0019234) and *IL10* (qHsaCED0003369) from Biorad.

B2M served as a reference gene with stable B2M expression over all groups, and relative target mRNA expression levels were compared to PBS-treated macrophages, which served as the control treatment. Relative gene expression was calculated as delta-delta Cp values, and results are depicted as x-fold expression of PBS samples.

### 2.9. T Cell Chemotaxis

To measure chemotaxis of T cells towards conditioned medium (CM) of sEV-treated macrophages, macrophage supernatants were collected after 24 h and centrifuged at 12,000× *g*. CD8+ T cells were activated with Immunocult CD3/CD28 T cell activator (Stemcell Technologies, Cologne, Germany) and 10 ng/mL IL-2 for 6 h prior to seeding 200,000 cells per well on transwell inserts (3421, Costar Corning Incorporated, Corning, NY, USA) with a pore size of 5 µm. Tregs were seeded without prior activation onto the transwell inserts, also with a cell number of 200,000 cells per insert. A total of 500 µL of CM was added to the lower chamber, and after 1.5 h of incubation, the filters were removed carefully, and the cells present in the lower chamber were collected and counted by the flow cytometer Gallios (Beckmann Coulter, Brea, CA, USA) with a fixed time of acquisition of 1 min.

### 2.10. Statistical Analysis

Proteins detected in 3 out of 4 replicates in proteomics analysis were identified and used to generate the Venn diagram using the online tool InteractiVenn (https://www.interactivenn.net/) [31]. Further analysis was performed using the open-source software Perseus (version 2.0.7.0) [32]. Normalized abundances were transformed by log2 transformation and subtraction of the median of all values before performing an ANOVA. The ANOVA significant proteins were summarized by hierarchical clustering. To perform gene-set enrichment analysis, the web-based tool ShinyGO 0.85.1 [33] was used.

Data from other experiments were analyzed using GraphPad Prism 10.2.1. Data were checked for normal distribution, and pairwise comparisons were performed using Student’s *t* test for parametric and the Mann–Whitney test for non-parametric data.

**Figure 1:**
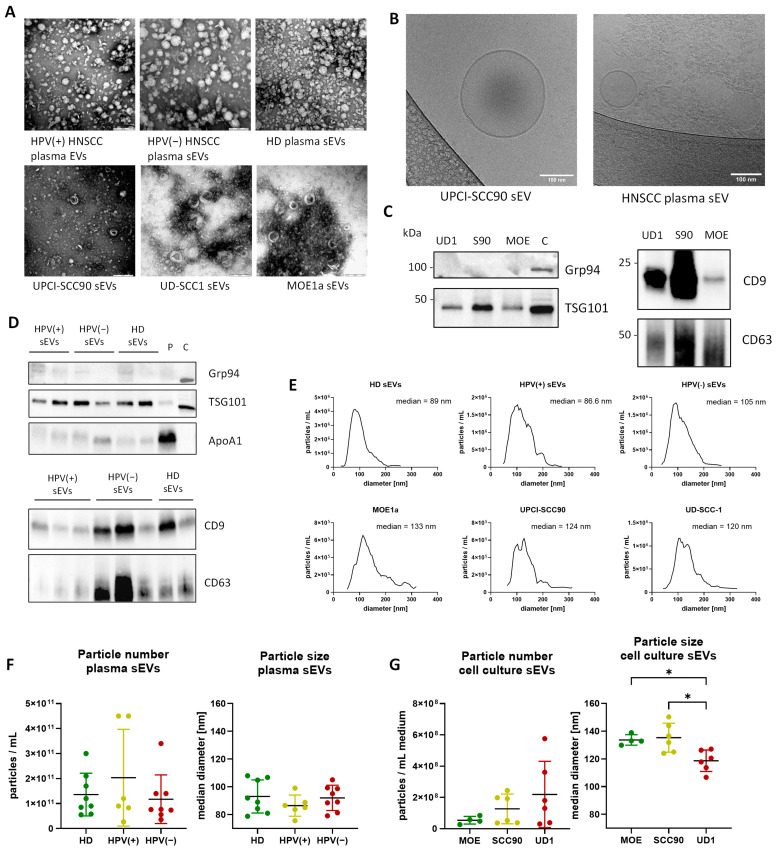
Characterization of isolated sEVs from plasma and cell culture supernatant. (**A**) Representative transmission electron microscopy images of plasma (upper row) or cell culture-derived (lower row) sEVs prepared by negative staining. Scale bar = 200 nm. (**B**) Representative cryo-electron microscopy images of sEVs isolated from cell culture (left) or from plasma (right). Scale bar = 100 nm. (**C**,**D**) Western blot analysis of cell culture-derived sEVs (**C**) or plasma-derived sEVs (**D**) to confirm the presence of sEV-specific markers TSG101, CD9, and CD63, and the absence of contaminant Grp94 and ApoA1 (only plasma-derived sEVs). UD1 = sEVs from UD-SCC-1 cells, S90 = sEVs from UPCI-SCC90 cells, MOE = sEVs from MOE-1a cells, C = cell lysate, P = plasma sample. (**E**) Size distribution of particles detected in sEV samples from plasma sEVs (upper row or cell culture-derived sEVs (lower row) determined by nanoparticle tracking analysis. (**F**,**G**) Particle number per mL and particle size depicted as median diameters from plasma sEVs (**F**) or from cell culture-derived sEVs (**G**). * *p* < 0.05.

**Figure 2:**
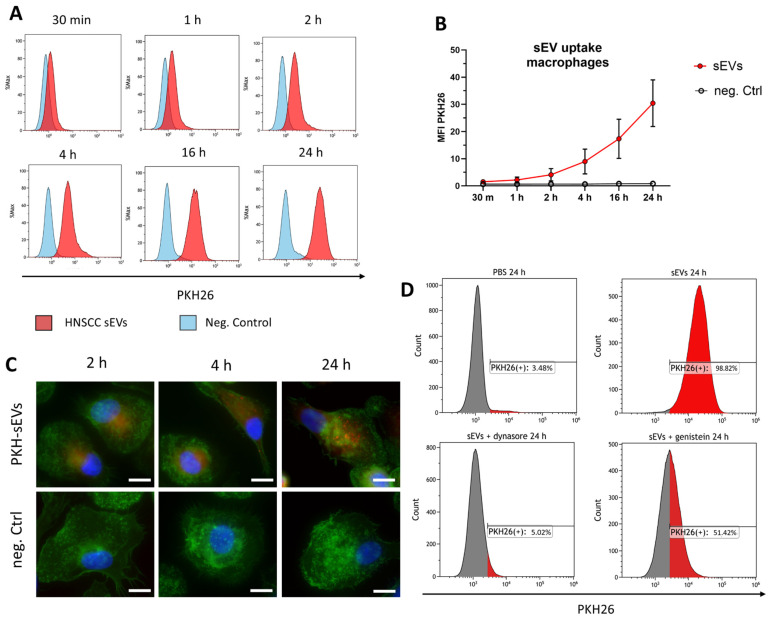
Internalization of PKH26-labeled sEVs by macrophages. (**A**) Representative histograms displaying the median fluorescence intensity (MFI) of PKH26 in macrophages incubated with PKH26-labeled sEVs (red) or negative control (blue) for 30 min, 1 h, 2 h, 4 h, 16 h, and 24 h, measured by flow cytometry analysis. As a negative control, cells were incubated with PBS, which had also been incubated with PKH26 dye. (**B**) Time-dependent sEV uptake by macrophages measured by flow cytometry from three independent replicates. Depicted are mean values from three independent experiments at each time point with error bars indicating SD. (**C**) Microscopy images of macrophages with internalized PKH26-labeled sEVs (red) and stained actin cytoskeleton (green) and nuclei (blue) after 2 h, 4 h, and 24 h of incubation. Scale bar = 10 µm. (**D**) Representative histograms of macrophages incubated with uptake inhibitors dynasore and genistein 15 min prior to incubation with PKH-labeled sEVs for 24 h. Adapted from Huber [34].

**Figure 3:**
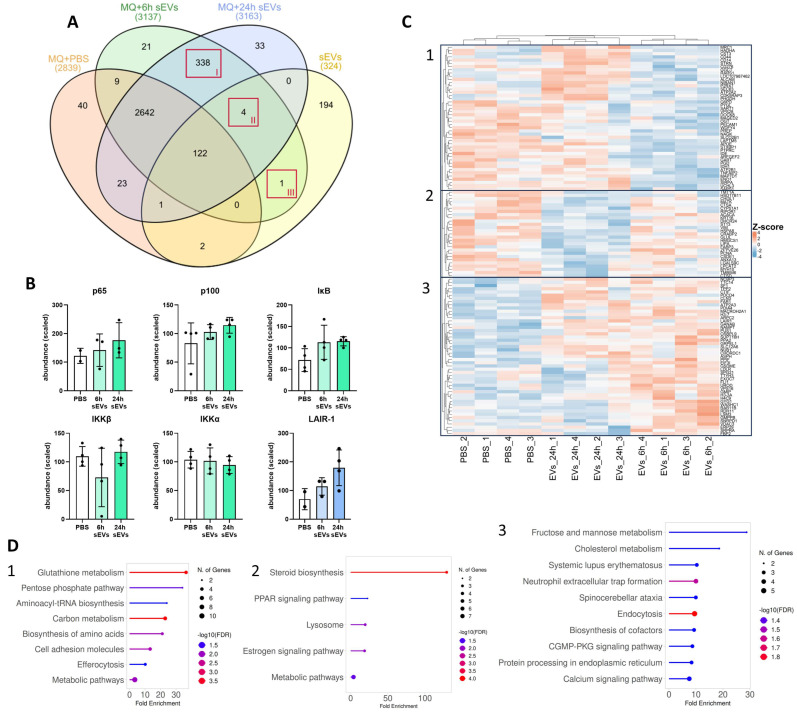
Time-dependent proteome alterations in macrophages incubated with HNSCC sEVs. sEVs isolated from plasma of HNSCC patients were analyzed by LC-MS/MS analysis as well as macrophages incubated with these sEVs for 6 h and 24 h or with PBS. (**A**) A Venn diagram of detected proteins in macrophages incubated with PBS (MQ + PBS), macrophages incubated with sEVs for 6 h (MQ + 6h sEVs) or 24 h (MQ + 24h sEVs), or isolated sEVs (sEVs) was generated using the free web-based tool InteractiVenn [31]. Proteins that were detected in at least 3 out of 4 replicates were taken for analysis. (**B**) Several NF-κB-related proteins were detected in the group of proteins highlighted as I in (**A**). (**C**) Hierarchical clustering of significantly different proteins in macrophage samples incubated with or without sEVs based on the z-score. Each sample group consists of 4 replicates with macrophages generated from one Buffy coat and HNSCC sEVs from 4 high-stage HNSCC patients. sEVs measured by MS analysis were the same samples as used for macrophage incubation. (**D**) KEGG annotation enrichment analysis performed on proteins highlighted as blocks 1, 2, and 3 in (**C**). Analysis was performed using the free online tool ShinyGO [33]. Figure adapted from Huber [34].

**Figure 4:**
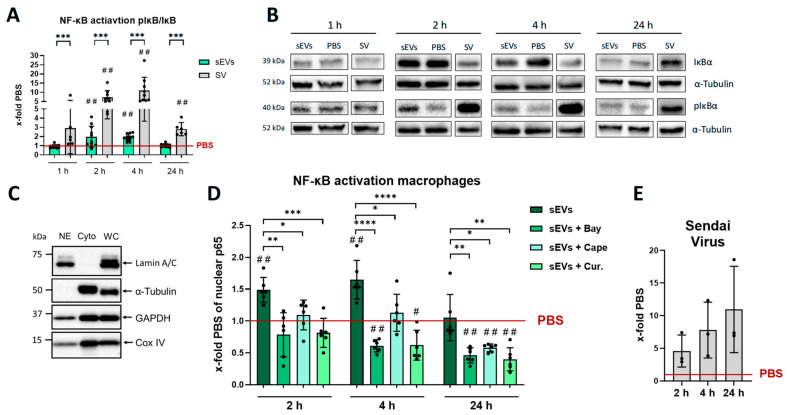
NF-κB activation in macrophages after incubation with HNSCC sEVs. (**A**,**B**) Macrophages were incubated with 15 µg of HNSCC sEVs, PBS as a negative control, or Sendai virus (SV) as a positive control for 1 h, 2 h, 4 h, and 24 h, and activation of the NF-κB signaling pathway was determined by measuring the ratio of pIkB and IkB. (**A**) NF-κB activation in macrophages incubated with sEVs and SV normalized to PBS. (**B**) Representative images of Western blots used to quantify NF-κB activation in macrophages (in (**A**)). (**C**–**E**) Canonical NF-κB signaling was measured by measuring levels of p65 present in the nuclear fraction of macrophages incubated with HNSCC sEVs with or without NF-κB inhibitors. (**C**) Representative Western blot to confirm successful separation of nuclear from cytoplasmic fraction. NE = nuclear extracts, Cyto = cytoplasmic fraction, WC = whole cell lysates. (**D**) NF-κB activation was assessed by measuring the nuclear translocation of p65. Depicted are p65 levels in nuclei of macrophages incubated with HNSCC sEVs, or co-incubated with HNSCC sEVs and NF-κB inhibitors: Bay 11-7082 (Bay), Caffeic acid phenethylester (Cape), or curcumin (Cur.), and normalized to PBS control. Dots represent individual sEV donors (*n* = 6) from experiments using macrophages from three different buffy coats. (**E**) Nuclear p65 levels in macrophages treated with the positive control Sendai virus (SV) were normalized to PBS controls, with dots representing experiments with macrophages from three different buffy coats. For better visibility of the results and due to very high differences in levels of NF-κB activation, the positive control was shown separately. Depicted in all graphs are mean values and SD with dots representing individual sEV donors. Differences with statistical significance were indicated with stars above the respective comparison with * representing *p* < 0.05; **: *p* < 0.01, ***: *p* < 0.005, and ****: *p* < 0.0001. # above bars represent statistically significant difference in comparison to PBS control with # representing *p* < 0.05; ##: *p* < 0.01. All other comparisons were not significant. Adapted from Huber [34].

**Figure 5:**
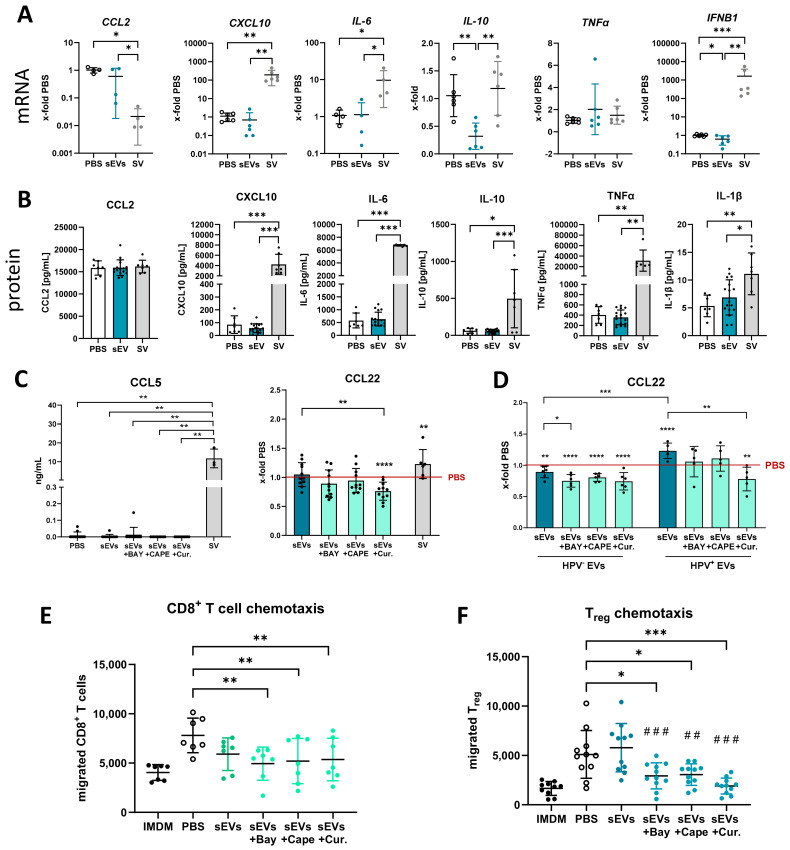
Downstream effects of NF-κB activation by sEVs. (**A**) RNA expression levels of several cytokines in macrophages incubated with PBS, HNSCC sEVs, or Sendai virus (SV) for 24 h. (**B**) Levels of cytokines secreted by macrophages after incubation with PBS, sEVs, or Sendai virus for 24 h measured by Milliplex Multiplex assay. (**C**) Levels of CCL5 and CCL22, which are among NF-κB-target genes, and co-incubation with NF-κB inhibitors: Bay, Cape, and Curcumin, measured by ELISA. (**D**) Levels of CCL22, measured in (**C**) and separated into macrophages incubated with HNSCC sEVs from HPV-positive patients (HPV+) or from HPV-negative HNSCC patients (HPV−). (**E**,**F**) Chemotaxis of CD8+ T cells (**E**) or CD4 + CD39+ regulatory T cells (Treg) (**F**) towards fresh IMDM medium (IMDM) or supernatant of macrophages incubated with PBS or HNSCC sEVs with or without NF-κB inhibitors. Statistical analysis was performed to compare the number of migrated cells between different conditions of macrophages. IMDM was used as a negative control and not included in the statistical analysis. Differences with statistical significance were indicated with stars above the respective comparison, with * representing *p* < 0.05; ** *p* < 0.01, *** *p* < 0.005, and ****: *p* < 0.0001. above bars represent statistically significant difference in comparison to PBS control with ##: *p* < 0.01, and ###: *p* < 0.005. All other comparisons were not significant. Adapted from Huber [34].

Parts of Figures 1–5 were adjusted from the Dissertation of the first author [34].

## 3. Results

### 3.1. Clinical Parameters of sEV Donors

Table 1 shows the clinical parameters of all sEV donors. The group of HNSCC patients, consisting of 64 donors, was slightly older, with a mean age at sample collection of 63.9 years, while HD (*n* = 10) had a mean age of 59.6 years. Alcohol as well as tobacco consumption was higher in HNSCC patients compared to HDs, and most donors in both groups were male. In most HNSCC patients, the primary tumor was located in the oropharynx, followed by the oral cavity, hypopharynx, larynx, and nasopharynx, with 79.7% of patients having lymph node metastases (N+). Most HNSCCs were classified as high-stage tumors in UICC stage IV and III, and the tumor size was classified as T3 or T4 for most patients. HPV status was defined based on HPV16 DNA detection and p16 immunohistochemistry. If both parameters were positive, the tumor was classified as HPV-positive. The standard diagnostic procedure in the University Hospital Ulm is HPV16 DNA testing, as HPV16 is the predominant high-risk HPV subtype in HNSCCs. Therefore, the terms ‘HPV(+)’ and ‘HPV(−)’ refer to HPV16-positive and HPV16-negative cases, respectively, throughout this manuscript. Other subtypes were not assessed.

In the current cohort, 31.3% of the HNSCC patients were tested positive for HPV, and 48.4% were HPV(−), while 20.3% were not tested because of tumor location other than oropharynx or oral cavity, or for unknown reasons.

In Table 2, the parameters of the HNSCC patients from Table 1 are shown, which were used in experiments comparing the effects of HPV(+) and HPV(−) sEVs. In these cohorts, tumors were mainly located in the oropharynx, followed by the oral cavity, and only very few patients had tumors in other locations. HPV(+) HNSCC patients were slightly younger, with a mean age of 61.5 years compared to 65.4 years in HPV(−) HNSCC patients, and alcohol and tobacco consumption were higher in HPV(−) HNSCC patients.

### 3.2. sEVs Characterization and Internalization by Macrophages

Characterization of sEVs is shown in Figure 1, where morphology, size distribution, and presence of sEV markers confirm successful isolation of sEVs. Figure 1A shows the morphology of sEVs isolated from blood plasma and cell lines with the typical cup-shaped morphology, especially in cell line-derived sEVs, but also in plasma sEVs. In Figure 1B, the representative cryo-EM images show vesicles with a double-layer membrane, also characteristic of sEVs. Figure 1C,D confirms the presence of sEV markers CD63, CD9, and TSG101 in isolated sEVs from cell culture and blood plasma, and substantially reduced signals of plasma contaminant ApoA1 and cellular contaminant Grp94. The particle size ranged from 50 to 180 nm in plasma-derived sEVs and 50 to 220 nm in cell line-derived sEVs, with sEVs from UD-SCC-1 cells being significantly smaller compared with MOE-1a- and UPCI-SCC90-derived sEVs. Furthermore, the particle counts were smaller in cell-culture-derived sEVs compared to plasma-derived sEVs, with no significant differences between different cell lines. Plasma-derived sEVs did not show significant differences based on pathological characteristics.

### 3.3. Internalization of sEVs by Macrophages

PKH-labeled sEVs were internalized by macrophages as early as 30 min after incubation, as observed by the shift in the median fluorescence intensity (MFI) in Figure 2A and uptake further increased with prolonged incubation (Figure 2A,B). Microscopy images of sEV-treated macrophages further demonstrated internalization of sEVs and accumulation around the nucleus (Figure 2C). Co-incubation with uptake inhibitors almost completely reduced the percentage of PKH-positive macrophages after treatment with dynasore and reduced it to approximately 50% after treatment with genistein (Figure 2D).

### 3.4. HNSCC sEVs Change the Proteome Profile of Macrophages

Figure 3A shows proteins detected in sEV samples and in macrophages incubated with or without sEVs, including the overlap between the different sample groups. The lists of proteins are provided in Supplementary File S1. The proteins highlighted with (II) and (III) were present in sEVs and in macrophages incubated with sEVs, but not in PBS-treated macrophages, and may therefore represent proteins directly transferred to macrophages via sEVs. However, the number of only 5 proteins is relatively low compared to the 324 proteins detected in sEVs and over 3100 proteins in sEV-treated macrophages. The proteins highlighted with (I) were more abundant and can be considered to be upregulated following sEV incubation, since they were only detected in sEV-treated, but not in PBS-treated, macrophages. Interestingly, some of the proteins found in this group were associated with the NF-κB signaling pathway (Figure 3B), with p65 and IκB being increased by sEV incubation. Supervised hierarchical clustering of common proteins detected in macrophages incubated with or without sEVs revealed that most replicates clustered according to their groups (Figure 3C). However, clustering did not follow a chronological pattern, as PBS-treated samples clustered between the 6 h and 24 h time points. Based on protein expression patterns, three segments were identified, which were further investigated by gene-set enrichment analysis as shown in Figure 3D. Proteins downregulated after 6 h of sEV incubation, but upregulated after 24 h (segment 1) were associated with metabolic and biosynthetic pathways, as well as “cell adhesion molecules” and “efferocytosis”. Proteins downregulated after 24 h of sEV incubation (segment 2) are mainly involved in steroid biosynthesis, but also “PPAR signaling”, “Lysosome”, “Estrogen signaling pathway”, and “Metabolic pathways”. Proteins, which were mainly increased by sEV incubation (segment 3), are associated with several metabolism and signaling pathways, as well as “Endocytosis” and “Protein processing in the endoplasmic reticulum”.

### 3.5. sEV-Dependent NF-κB Activation in Macrophages

To assess NF-κB modulation by HNSCC sEVs, NF-κB activation was measured in macrophages incubated with sEVs. Western blot data revealed a significant activation of the NF-κB signaling pathway after sEV incubation for 1 h, 2 h, and 4 h (Figure 4A,B), but not after 24 h of incubation. To measure nuclear p65, the cells were fractionated into cytoplasmic and nuclear fractions. Measurement of nuclear p65 levels allowed specific assessment of canonical NF-κB pathway activation. Figure 4C confirms successful fractionation, with Lamin A/C being present in nuclear extracts but absent in the cytoplasmic fraction, while α-Tubulin was detected in the cytoplasmic and whole cell lysates but not in nuclear extracts. NF-κB nuclear translocation, as measured by p65 ELISA of nuclear extracts, was increased in sEV-treated macrophages after 2 h and 4 h, with normalized levels after 24 h (Figure 4D). This activation was inhibited by co-incubation with NF-κB inhibitors: Bay, CAPE, and Curcumin, which decreased NF-κB activation even below the basal levels. Incubation with the positive control, Sendai virus (SV), increased NF-κB activation with increasing incubation time (Figure 4E).

### 3.6. Downstream Effects of NF-κB Activation

As p65 is a transcription factor regulating the expression of several immune-related genes, the expression of several cytokines was determined. RNA expression of IL-10 and IFNB1 was significantly decreased by HNSCC sEVs (Figure 5A). At the protein level, sEV incubation did not significantly affect cytokine production (Figure 5B). The positive control, Sendai virus, significantly increased cytokine expression levels on the protein level, except for CCL2, while at the RNA level, expression of CXCL10, IL-6, and IFNB1 increased, and levels of CCL2 decreased significantly. As important NF-κB target genes, production of CCL5 and CCL22 was measured in macrophages incubated with sEVs and NF-κB inhibitors. CCL5 production was not induced by sEVs and also CCL22 production seemed to remain unaffected (Figure 5C). However, when sEVs were separated based on the HPV status of HNSCC sEV donors, HPV(+) sEVs significantly increased CCL22 production, while HPV(−) sEVs significantly decreased CCL22 production (Figure 5D).

Since macrophages are important modulators of the TME, the effect of HNSCC sEVs on macrophage-mediated chemotaxis of T cells was investigated. Macrophage incubation with sEVs decreased chemotaxis of CD8(+) T cells slightly, but not statistically significantly (Figure 5E), and chemotaxis was further reduced after treatment with NF-κB inhibitors. In contrast, Treg chemotaxis was slightly increased upon sEV incubation (Figure 5F), and co-incubation with NF-κB inhibitors decreased Treg migration significantly compared to PBS-treated macrophages.

### 3.7. Different Effects of sEVs Dependent on HPV Status

**Figure 6:**
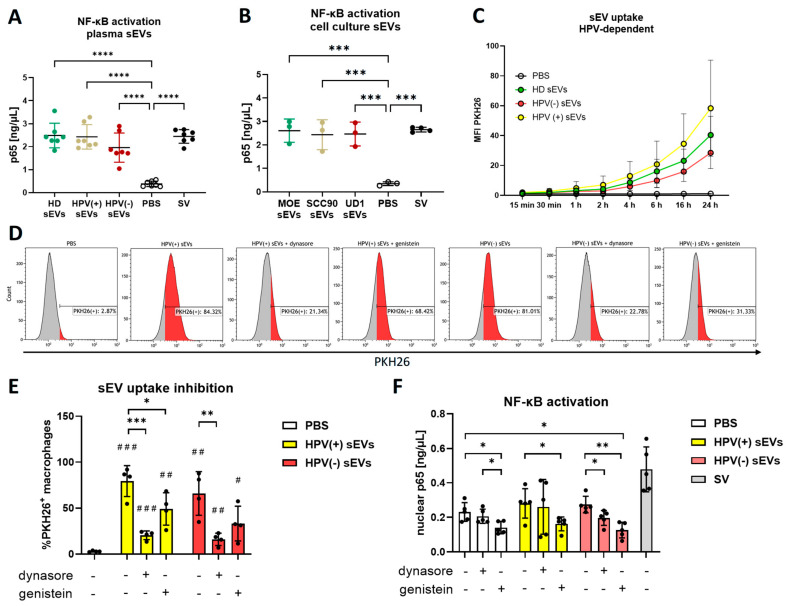
Different effects of HNSCC sEVs are dependent on HPV status. (**A**,**B**) Activation of NF-κB signaling, measured by determining levels of nuclear p65. Macrophages were incubated with PBS as a negative control or Sendai virus (SV) as a positive control, and with plasma-derived sEVs from HD, HPV(+), and HPV(−) HNSCC patients (**A**) or with cell culture-derived sEVs from MOE-1a (healthy epithelial cell line), UPCI-SCC90 (HPV(+) HNSCC cell line), or UD-SCC-1 (HPV(−) HNSCC cell line). (**B**) Experiments were performed with macrophages from three donors, and dots represent individual sEV donors. (**C**) Internalization dynamics of sEVs derived from healthy donors (HD), HPV(+) and HPV(−) HNSCC patients by measuring fluorescence intensities of macrophages incubated with PKH26-labeled sEVs. Depicted are mean values and SD of median fluorescence intensities from 3 independent experiments. (**D**) Representative histograms from measuring inhibition of sEV uptake by uptake inhibitors: dynasore and genistein by flow cytometry. (**E**) Quantification and statistical analysis of sEV uptake inhibition by dynasore and genistein measured by flow cytometry. (**F**) NF-κB activation after incubation with sEVs from HPV(+) and HPV(−) HNSCC patients and co-incubation with sEV uptake inhibitors: dynasore and genistein. Experiments were performed with macrophages from two donors, and dots represent individual sEV donors. Differences with statistical significance were indicated with stars above the respective comparison with * representing *p* < 0.05; **: *p* < 0.01, ***: *p* < 0.005, and ****: *p* < 0.0001. # above bars represent statistically significant difference in comparison to PBS control with # representing *p* < 0.05; ##: *p* < 0.01, and ###: *p* < 0.005. All other comparisons were not significant. Adapted from Huber [34].

Incubation of macrophages with HD, HPV(+), and HPV(−) sEVs revealed significantly increased NF-κB activation induced by all sEV types compared with PBS-treated macrophages. A trend toward lower NF-κB activation was observed for HPV(−) sEVs compared with HD and HPV(+) sEVs, although this difference did not reach statistical significance (Figure 6A). Also, cell line-derived sEVs significantly increased NF-κB activation, but no reduction in NF-κB activation was observed for HPV(−) sEVs compared with the other sEV types (Figure 6B).

To further investigate HPV-dependent differences in plasma-derived sEVs, uptake dynamics of sEVs from HD, HPV(+), and HPV(−) HNSCC patients were evaluated and revealed a trend toward increased internalization of HPV(+) sEVs compared with HD and especially HPV(−) sEVs (Figure 6C). To investigate the role of sEV internalization for NF-κB activation, uptake of HPV(+) and HPV(−) sEVs by macrophages was inhibited using uptake inhibitors dynasore and genistein (Figure 6D,E). Dynasore reduced the percentage of sEV-positive macrophages from approximately 90% to 20%, whereas genistein reduced it to around 45% (Figure 6E).

Co-incubation with genistein significantly decreased NF-κB activation induced by HPV(+) and HPV(−) sEVs. However, NF-κB activation was also reduced in PBS-treated macrophages, indicating an sEV-independent inhibitory effect of genistein (Figure 6F). In contrast, dynasore did not affect basal NF-κB activation in PBS-treated macrophages or NF-κB activation induced by HPV(+) sEVs, but significantly decreased NF-κB activation by HPV(−) sEVs.

## 4. Discussion

Tumor-associated macrophages (TAMs) are important regulators of tumor progression and immune evasion in HNSCCs and also contribute to therapy resistance [35]. Tumor-derived sEVs modulate the immunological functions of macrophages [16,17], and circulating sEVs were shown to induce systemic immunosuppression in tumor patients, including HNSCC patients [14,15]. In contrast to studies investigating cell line-derived sEVs, we demonstrate here that plasma-derived sEVs from HNSCC patients modulate macrophage proteomes and activate NF-κB signaling, revealing systemic sEV-mediated immune regulation of macrophages with potential implications for novel therapeutic strategies. This approach allows investigation of more complex sEV-mediated effects in a patient-derived context, which may include contributions from sEVs of different cellular origins. Despite the increased variability inherent to this patient-derived approach, exploratory analyses revealed differences associated with HPV status, including differential modulation of canonical NF-κB signaling and sEV uptake by HPV(+) versus HPV(−) HNSCC-derived sEVs.

MS-based global proteomic analysis of macrophages revealed p65 stabilization and possible NF-κB activation after incubation with plasma-derived sEVs from HNSCC patients, which was confirmed by Western blot and p65 nuclear translocation assays. In these experiments, NF-κB activation was increased after 2 h and 4 h of incubation with HNSCC sEVs, but not after 24 h, while the positive control, Sendai Virus, increased NF-κB activation further with time. This is consistent with a transient activation of NF-κB by HNSCC sEVs, followed by a return to basal levels, potentially through negative feedback mechanisms. LAIR-1 is such an inhibitor of NF-κB activation and was found to be increased with prolonged sEV incubation in the data of MS analysis, further supporting this hypothesis. Co-incubation with NF-κB inhibitors decreased sEV-induced NF-κB activation, in some cases even below basal levels, which is likely due to the constitutive NF-κB activity present in monocytic cells, including macrophages [36].

Supervised hierarchical clustering revealed that macrophages treated with HNSCC sEVs for 24 h were closer to PBS-treated cells than those incubated with sEVs for 6 h, supporting the hypothesis that sEV-induced effects are transient. Some metabolism pathways, but also “Efferocytosis” and “Cell adhesion molecules” seemed to be downregulated by sEVs at first, but upregulated after 24 h. The modulation of efferocytosis is consistent with our previous RNA sequencing and functional data demonstrating enhanced efferocytosis upon HNSCC sEV exposure for 48 h [37]. In addition, PPAR signaling was affected by HNSCC sEVs in the proteomics analysis, as well as in RNA-sequencing data from our previous publication [37]. As peroxisome proliferator-activated receptors (PPARs) regulate lipid metabolism and immune functions and can be activated by fatty acids present in sEVs [38,39,40], these findings suggest that HNSCC sEVs transiently reprogram macrophage metabolic and immune pathways.

As a transcription factor, p65 was expected to regulate the expression of several inflammatory target genes. However, only reduced mRNA levels of IL-10 and IFN1B were observed. Type I interferons can exert dose-dependent effects, where high levels promote apoptosis in tumor cells, whereas low levels may support tumor growth [41]. Thus, downregulation of IFN1B in macrophages by sEVs may contribute to a tumor-promoting environment. IL-10 is an important anti-inflammatory cytokine, which is often secreted by immunosuppressive TAMs and inhibits cytotoxic T-cell functions [42]. However, while mRNA levels of IL-10 were downregulated by sEV incubation, protein levels remained stable, which can have various reasons, including differences in stability, time delay in transcription and translation processes, and post-transcriptional modifications [43]. Furthermore, although NF-κB activation is frequently associated with pro-inflammatory cytokine production, NF-κB also regulates numerous genes involved in cell survival and metabolism. Proteomic analysis of sEV-treated macrophages revealed enrichment of pathways related to cellular metabolism, endocytosis, and protein processing rather than inflammatory cytokine production. Therefore, sEV-induced NF-κB activation may contribute to proteomic remodeling without necessarily resulting in biologically relevant cytokine secretion.

Interestingly, separating sEV donors according to HPV status revealed significantly increased levels of CCL22 by HPV(+) sEVs, but not by HPV(−) sEVs. CCL22 produced by TAMs in esophageal squamous cell carcinoma patients promoted metastasis formation and chemoresistance [44,45], suggesting altered macrophage-mediated immune regulation. Although CCL22 is a known Treg-attracting chemokine [46], Treg chemotaxis remained unaffected by altered CCL22 levels in this study, and no HPV-dependent differences were observed. In contrast, CD8+ T cell chemotaxis was slightly decreased towards sEV-treated macrophages, indicating inhibition of anti-tumor T cell infiltration by sEV-treated macrophages. This effect was further enhanced by NF-κB inhibition, indicating that NF-κB signaling also contributes to the regulation of T-cell attraction. Since increased T cell infiltration is associated with favorable outcomes [7], altered T cell chemotaxis may represent one mechanism by which sEV-modulated macrophages influence immune cell recruitment within the TME.

Although sEV-induced NF-kB activation was confirmed by two independent methods, downstream effects on cytokine expression or T cell attraction remained modest. In contrast to the Sendai virus, sEVs only induced transient NF-κB activation. Since cytokine mRNA and protein levels were assessed after 24 h, it is possible that early transcriptional responses induced by sEVs were not captured in our experimental design. Moreover, NF-κB activation may result in selective rather than broad downstream effects, which will require further investigation.

Similar to CCL22 production, NF-κB activation was induced to higher levels by HPV(+) compared to HPV(−) sEVs, but similar to sEVs from HDs, with neither difference reaching statistical significance. In this assay, nuclear levels of p65 were determined, which belongs to the canonical NF-κB signaling pathway. All types of sEVs induced significantly increased canonical NF-κB signaling compared to the negative control, although activation was less pronounced with HPV(−) sEVs. mRNA and miRNA profiles, as well as protein contents of sEVs from HPV(+) and HPV(−) HNSCC cell lines, were shown to differ and cause functionally distinct effects in immune cells [47,48]. For instance, Harden et al. identified a set of miRNAs selectively packaged into EVs from HPV(+) cells, predicted to inhibit necrosis and apoptosis [49]. Differences in sEV cargo composition, as reported in the studies above, may represent one possible explanation for the increased canonical NF-κB activation induced by HPV(+) sEVs, although this was not directly assessed in the present study. In contrast, HPV(−) sEVs might preferentially engage non-canonical NF-κB signaling or other signaling pathways to induce immunosuppressive properties.

sEVs mediate cellular communication by transferring their cargo or interacting with cell surface receptors. In our experiments, only five proteins were found to be directly transferred by sEVs, although a large number of proteins were found to be altered after sEV incubation. This suggests sEV-mediated modulation of protein expression either by interacting with surface receptors or by delivering modulatory components other than proteins. Since the internalization of sEVs by macrophages was confirmed by uptake assays, this supports the hypothesis of cargo transfer. Furthermore, gene set enrichment analysis revealed pathways increased by sEV incubation after 6 h and 24 h include “Endocytosis”, “Cholesterol metabolism”, and “Protein processing in endoplasmic reticulum”, which can be explained by the uptake and processing of sEVs. Since sEVs accumulate around the nucleus after internalization, as observed in microscopy images, this suggests processing of internalized sEVs in the endoplasmic reticulum. As non-protein components, miRNAs were reported to be associated with sEVs and transferred to recipient cells, thereby mediating cellular changes in the recipient cells [50]. For example, EVs derived from esophageal cancer patients transformed macrophages into tumor-promoting TAMs by activating the PTEN/AKT/STAT6 signaling pathway via miR-21-5p [51]. Furthermore, sEVs from oral squamous cell carcinoma patients were reported to carry oncogenic miRNAs, which reprogram monocytes via the NF-κB pathway [52]. Another factor contributing to alterations in macrophage proteomes and signaling might be sEV-associated lipids, as fatty acids have been shown to be enriched in tumor-derived EVs and modulate dendritic cell function [53].

Inhibition of sEV uptake by incubation with dynasore decreased NF-κB activation induced by HPV(−) sEVs significantly, further indicating that internalization is necessary for NF-κB activation by HPV(−) sEVs. In contrast, inhibiting uptake of HPV(+) sEVs did not decrease NF-κB activation, suggesting activation of NF-κB through cell surface receptors by components of HPV(+) sEVs. There are several receptors located at the surface of macrophages that are able to activate NF-κB signaling, including Toll-like receptors (TLRs), TNF receptors, and many more [18]. Tumor-derived sEVs from colorectal cancer cells, for example, were shown to activate NF-κB in macrophages through TLR4 [54]. Other studies reported NF-κB activation in macrophages through HNSCC sEVs carrying TGFβ [55] or CD73 [17] and inducing a tumor-promoting phenotype, which might also be the underlying mechanism induced by HPV(+) sEVs. The different cargo composition of HPV(+) and HPV(−) sEVs, including differences in miRNAs, lipids, proteins, and surface-associated molecules, may contribute to the observed differences in macrophage responses by influencing vesicle uptake, receptor engagement, and subsequent signaling pathway activation.

To further investigate the differences induced by HPV(+) and HPV(−) HNSCC sEVs and in comparison to HD sEVs, sEVs derived from HPV(+) and HPV(−) HNSCC cell lines from one healthy epithelial cell line were used for further experiments. It has to be considered that plasma-derived sEVs not only consist of tumor-derived sEVs but also display a mixture of sEVs from different cellular origins, while cell line-derived sEVs only consist of sEVs from one cell type and therefore have a less complex composition. So, because of the more homogenous composition of cell line-derived sEVs, more distinct differences in NF-κB activation were expected by sEVs from HPV(+) and HPV(−) HNSCC cell lines. However, all cell culture-derived sEVs induced comparable levels of NF-κB activation, which contrasts with the trend towards differential activation observed with plasma-derived sEVs from HPV(+) versus HPV(−) patients. This discrepancy suggests that the mechanisms and effects underlying NF-κB activation by circulating plasma sEVs may differ from those induced by tumor cell line-derived sEVs alone, possibly reflecting the more complex and heterogeneous composition of plasma sEVs in vivo.

Tumor-derived sEVs are often reported to reprogram immune cells towards tumor progression; however, sEVs derived from other cell types, including immune cells, are also capable of modulating the immune response [56]. In HNSCCs, the immune landscape differs largely between HPV(+) and HPV(−) disease, and these differences might also be reflected in the activation of NF-κB signaling. HPV(+) HNSCC is characterized by persistent inflammation and elevated levels of immunomodulatory molecules, including immune checkpoints [5,57]. Interestingly, activated NF-κB signaling in HPV-associated HNSCCs was reported to portend improved outcomes [58], and HPV-associated HNSCC is more sensitive to anti-cancer treatment. Decreased NF-κB signaling by HPV(−) sEVs could therefore potentially be linked to the broader immunosuppressive profile reported in non-HPV-associated HNSCC patients, although this connection remains speculative. HPV(−) sEVs might be able to suppress the immune response by decreasing levels of canonical pro-inflammatory NF-κB signaling in macrophages. Supporting this hypothesis in a related immune cell type, incubation of dendritic cells with HNSCC sEVs revealed increased maturation and activation in DCs treated with HPV(+) sEVs, while HPV(−) sEVs decreased DC maturation and activation [59]. Beyond their effects on NF-κB signaling, tumor-derived sEVs carry tumor-associated antigens that are able to induce anti-tumor immune responses [60]. Many cancer-derived sEVs, however, also contain immunosuppressive molecules, which decrease the anti-tumor immune response.

Another factor that has to be considered when interpreting the different effects of HPV(+) and HPV(−) sEVs is the increased smoking and alcohol consumption by HNSCC patients, particularly in the HPV(−) cohort, as well as differences in age, sex distribution, and tumor localization. These variables reflect the well-established epidemiological characteristics of HPV-positive and HPV-negative HNSCCs but may also influence systemic immune responses [61,62] and the composition of circulating sEVs, and thus cannot be excluded as contributing factors to the observed effects.

A general limitation of using patient-derived plasma sEVs is the increased variability introduced by donor heterogeneity, which can obscure subtle sEV-induced differences and reduce statistical power. In addition, the sEV dose used in vitro does not directly correspond to a defined physiological exposure level in vivo: although it was selected based on preliminary dose-finding experiments and is comparable to measured plasma sEV concentrations, the actual exposure of tissue-resident macrophages to circulating sEVs is difficult to estimate, given the complex distribution and tissue accumulation of sEVs in vivo.

Despite these limitations, taken together, these findings support the hypothesis that circulating sEVs from HPV(+) and HPV(−) HNSCC patients differentially modulate systemic immune functions of macrophages, potentially contributing to the distinct immune landscapes and clinical behavior observed between these two HNSCC subtypes.

## 5. Conclusions

HPV(+) HNSCC patients are generally younger, respond better to therapy, and have a more favorable prognosis, whereas HPV(−) patients are typically older and present with more comorbidities, increasing their vulnerability to treatment-related adverse effects. Despite these differences, treatment de-escalation in HPV(+) disease has not yet been successful [63,64,65], and both groups currently receive comparable treatment regimens with similar impacts on quality of life. This underlines the need for improved, more tailored therapeutic approaches for HNSCC subgroups.

This study shows that plasma-derived sEVs from HNSCC patients modulate macrophage proteome and NF-κB signaling. sEVs from HPV(+) and HPV(−) patient cohorts exhibited distinct effects on NF-κB activation and macrophage responses, which were linked to differences in sEV-macrophage interaction dynamics, including vesicle uptake dependence.

Using patient-derived plasma sEVs, our findings demonstrate systemic sEV-mediated immune modulation in HNSCCs beyond direct tumor-immune cell interactions. These results provide a basis for further mechanistic studies on sEV-driven macrophage reprogramming, its role in shaping the immune landscape of HNSCCs, and its potential exploitation for HPV status-specific therapeutic approaches.

## Figures and Tables

**Table 1:** Clinical parameters of all sEV donors.

Mean Age [Years]	HNSCC Patients (*n* = 64)		Healthy Donors (*n* = 10)	
	63.9 Range: 45–88		59.6 Range: 37–72	
	*n*	%	*n*	%
Sex				
male	53	82.8	8	80.0
female	11	17.2	2	20.0
Smoking				
smokers	39	60.9	2	20.0
non-smokers	9	14.1	7	70.0
former smokers	16	25.0	1	10.0
Alcohol consumption				
yes	28	43.8	1	10.0
no	8	12.5	3	30.0
occasionally	21	32.8	6	60.0
former alcoholic	7	10.9	0	0.0
Primary tumor site				
Oral cavity	18	28.1		
Oropharynx	33	51.6		
Hypopharynx	7	10.9		
Larynx	4	6.3		
Nasopharynx	2	3.1		
HPV16 status				
positive	20	31.3		
negative	31	48.4		
not tested	13	20.3		
T status				
1	5	7.8		
2	18	28.1		
3	18	28.1		
4	23	35.9		
Nodal status				
N0	13	20.3		
N+	51	79.7		
UICC stage				
I	5	7.8		
II	9	14.1		
III	19	29.7		
IV	31	48.4		

**Table 2:** Clinical parameters of HNSCC patients for experiments comparing HPV16(+) and HPV16(−) HNSCC patients.

Mean Age [Years]	HPV16(+) HNSCC Patients (*n* = 20)		HPV16(−) HNSCC Patients (*n* = 22)	
	61.5		65.4	
	*n*	%	*n*	%
Sex				
male	14	70.0	21	95.5
female	6	30.0	1	4.5
Smoking				
smokers	7	35.0	13	59.1
non-smokers	7	35.0	2	9.1
former smokers	6	30.0	7	31.8
Alcohol consumption				
yes	5	25.0	11	50.0
no	5	25.0	1	4.5
occasionally	9	45.0	6	27.3
former alcoholic	1	5.0	4	18.2
Primary tumor site				
Oral cavity	3	15.0	6	27.3
Oropharynx	15	75.0	15	68.2
Hypopharynx	0	0	0	0.0
Larynx	0	0	1	4.5
Nasopharynx	2	10.0	0	0.0

## Data Availability

The dataset supporting the conclusions of this article is available in the Supplementary Material, and mass spectrometry raw data are available via ProteomeXchange with identifier PXD069623.

## Supplementary Materials

The following supporting information can be downloaded at: https://www.mdpi.com/article/10.3390/cancers18142219/s1, Table S1: Supplement_Proteomics_analysis; Supplementary File S1: Mass spectrometry raw data are available via ProteomeXchange with identifier PXD069623.

## Abbreviations

The following abbreviations are used in this manuscript:

HNSCC	Head and Neck Squamous Cell Carcinoma
HPV	Human Papilloma Virus
NF-kB	Nuclear Factor kappa B
sEVs	Small extracellular Vesicles
TAM	Tumor-associated Macrophage
TME	Tumor Microenvironment
