# Supplementary figures and images for: Plasma-Derived sEVs from HNSCC Patients Differentially Regulate NF-κB Signaling in Macrophages Depending on the HPV Status
Source: Cancers (Basel). 2026 Jul 9;18(14):2219. doi: 10.3390/cancers18142219 (PMC13406845; doi:10.3390/cancers18142219)

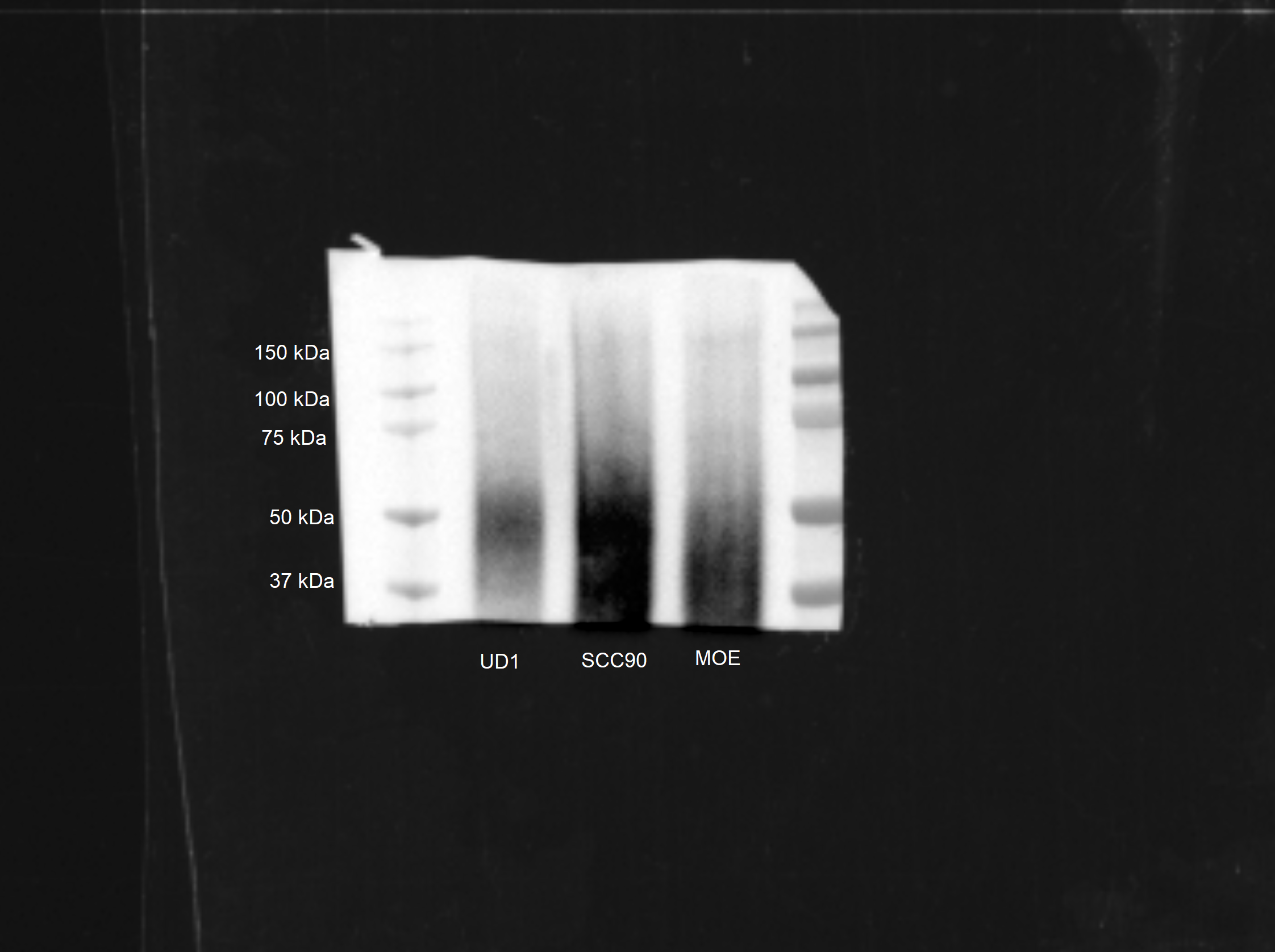

Supplement: Supplementary file 1 [file cancers-18-02219-s001.zip › supplement_proteomics_WB/full_WB_images_and_data/Fig1C_Cell_Culture_sEVs_CD63.tif]

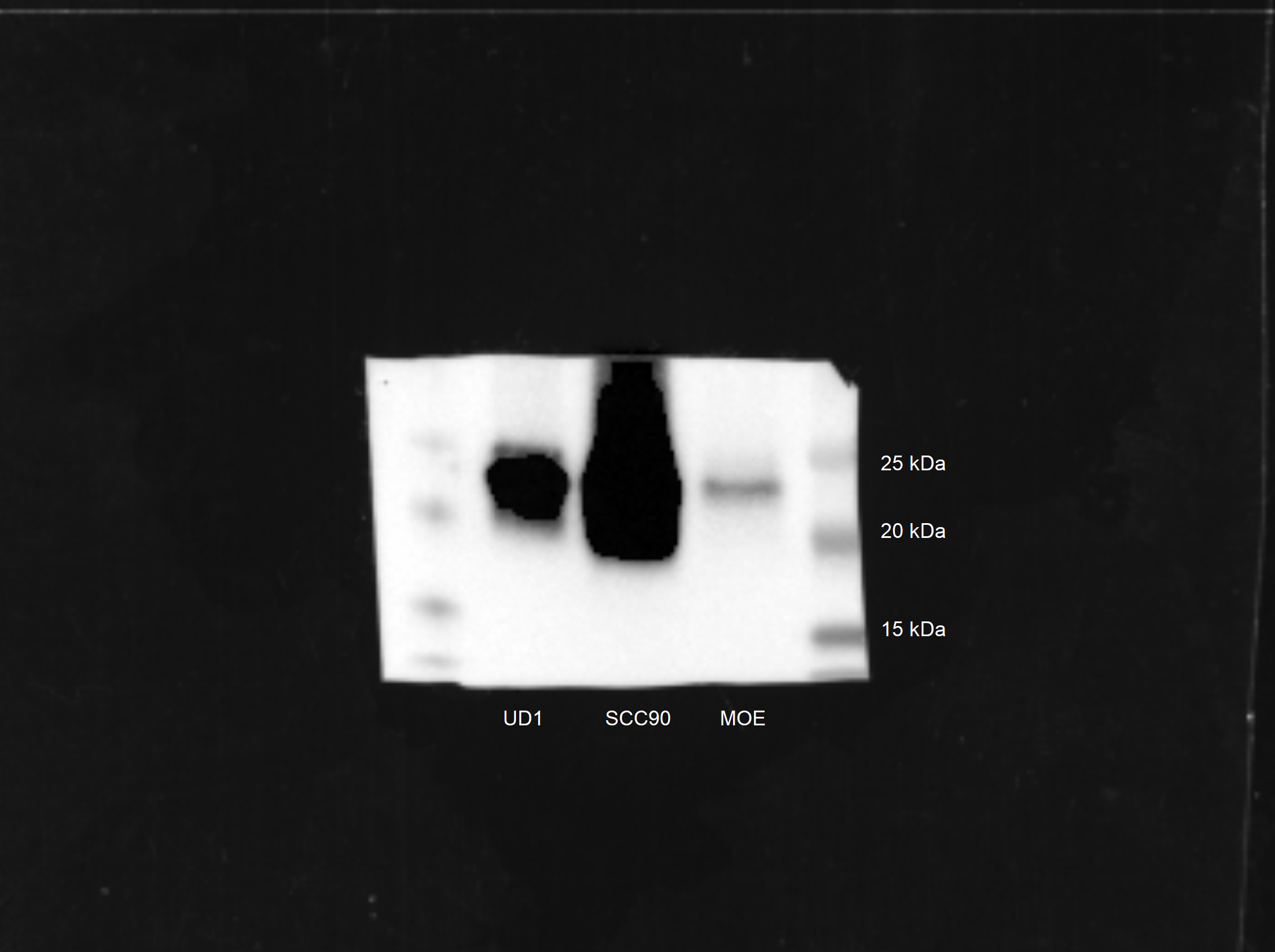

Supplement: Supplementary file 1 [file cancers-18-02219-s001.zip › supplement_proteomics_WB/full_WB_images_and_data/Fig1C_Cell_Culture_sEVs_CD9.tif]

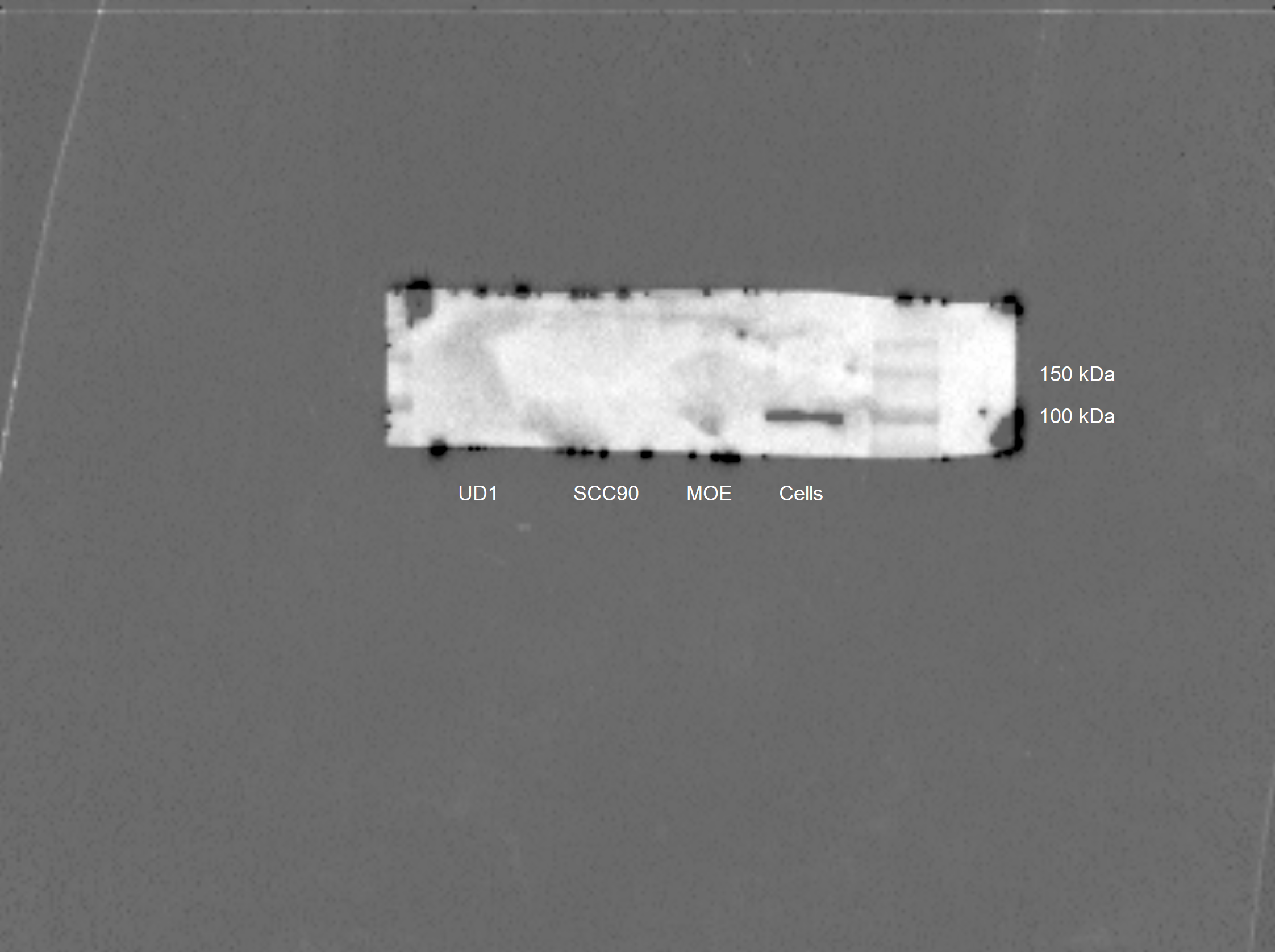

Supplement: Supplementary file 1 [file cancers-18-02219-s001.zip › supplement_proteomics_WB/full_WB_images_and_data/Fig1C_Cell_Culture_sEVs_Grp94.tif]

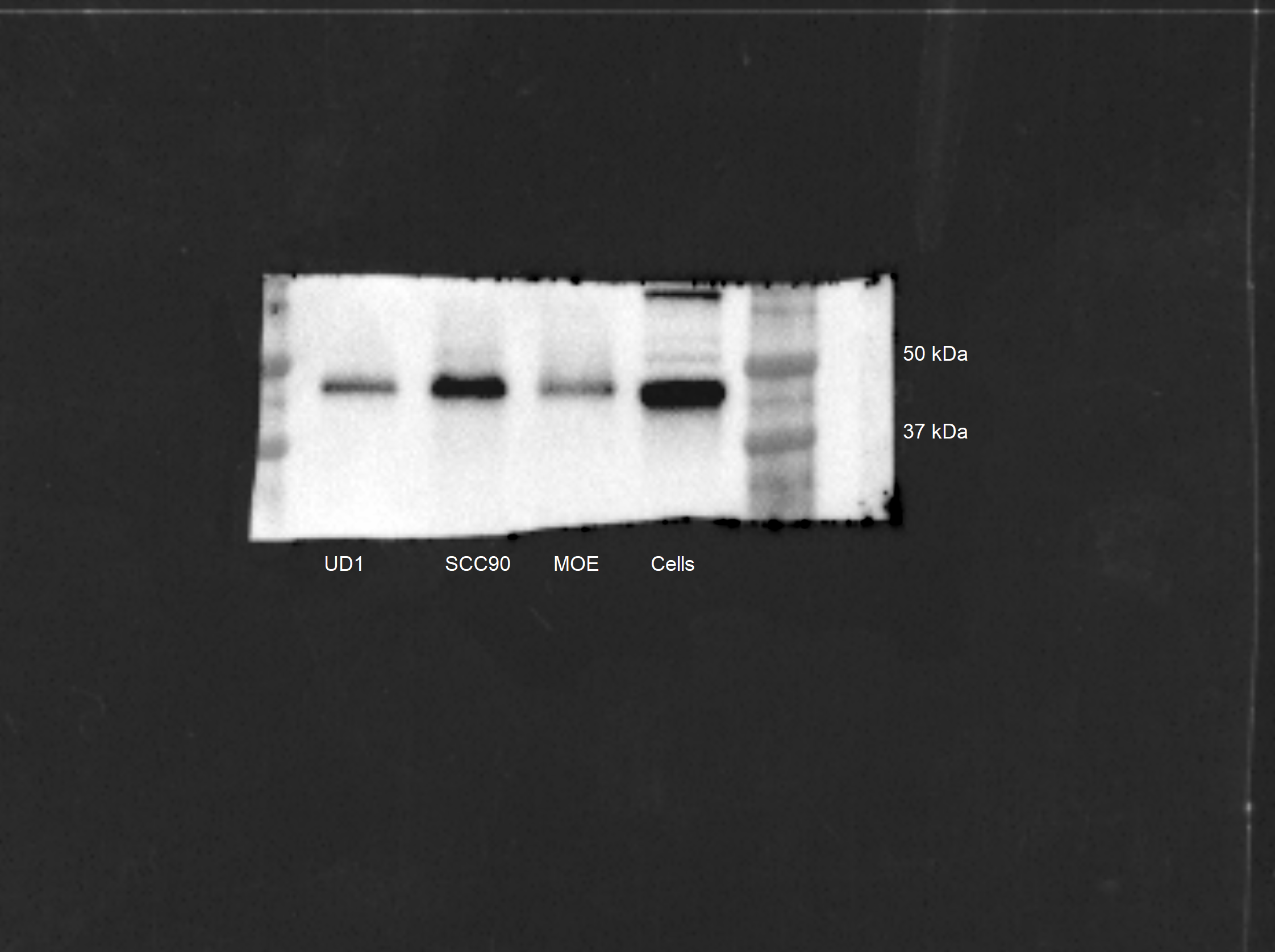

Supplement: Supplementary file 1 [file cancers-18-02219-s001.zip › supplement_proteomics_WB/full_WB_images_and_data/Fig1C_Cell_Culture_sEVs_TSG101.tif]

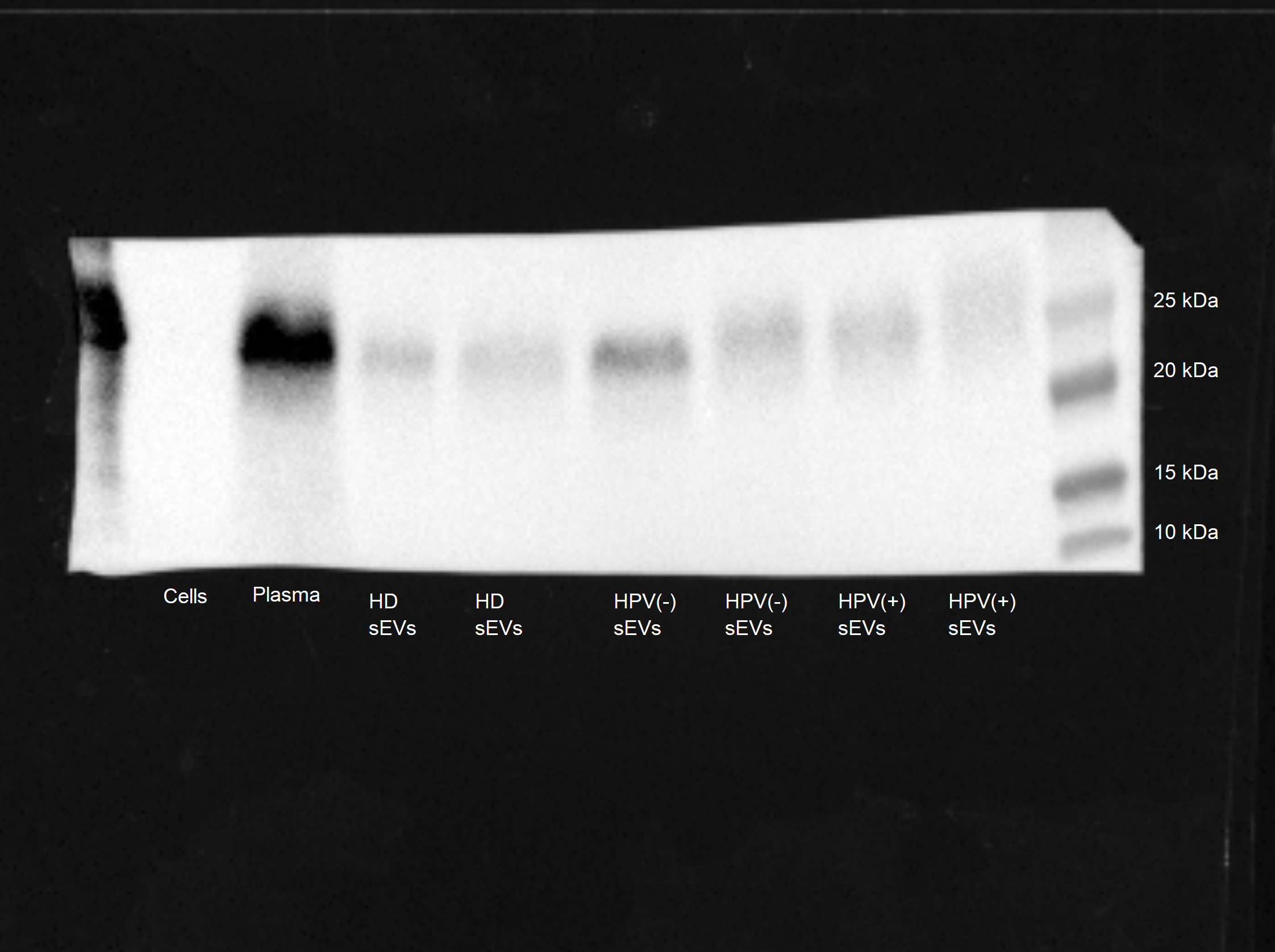

Supplement: Supplementary file 1 [file cancers-18-02219-s001.zip › supplement_proteomics_WB/full_WB_images_and_data/Fig1D_Plasma_sEVs_ApoA1.tif]

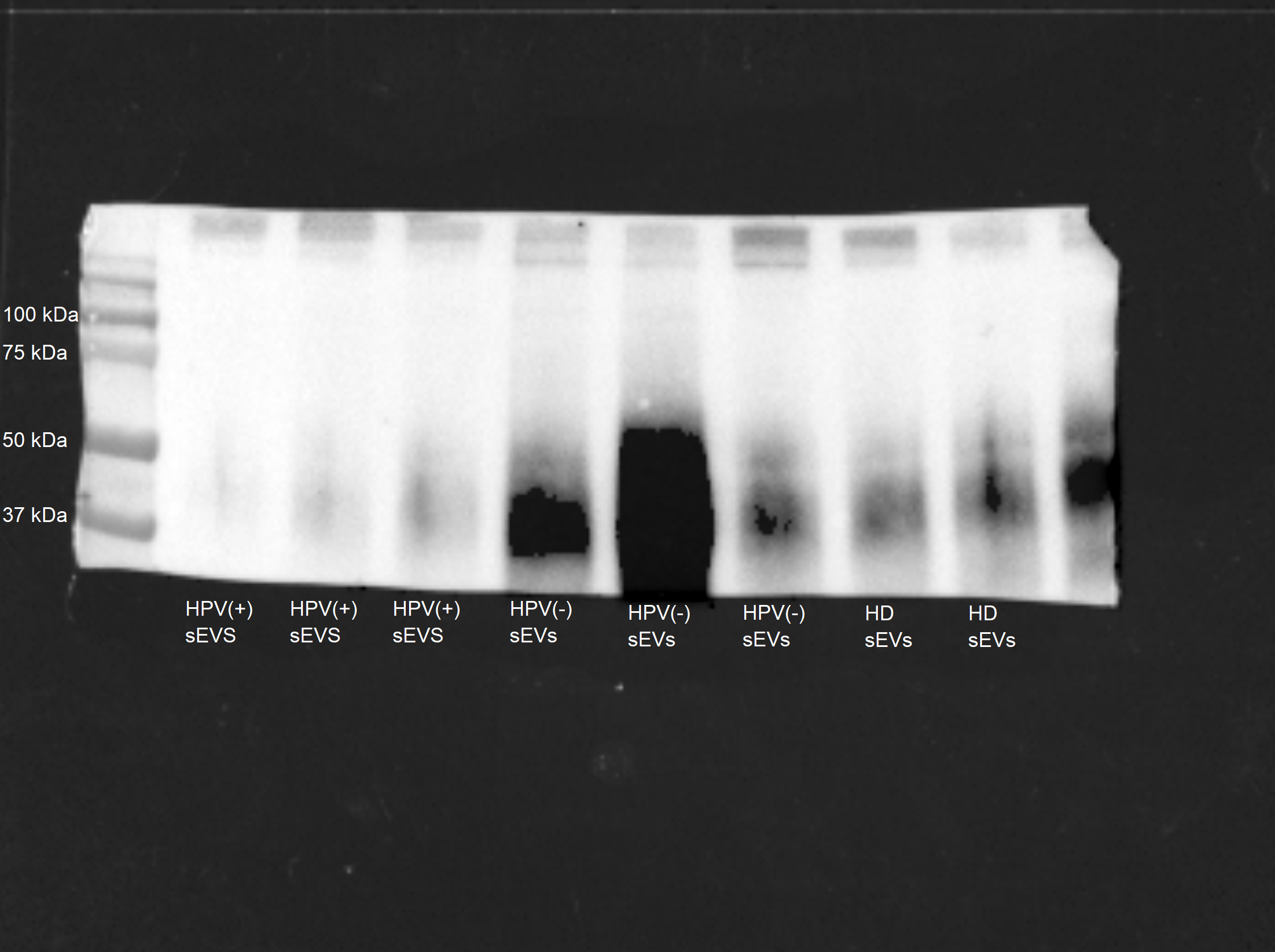

Supplement: Supplementary file 1 [file cancers-18-02219-s001.zip › supplement_proteomics_WB/full_WB_images_and_data/Fig1D_Plasma_sEVs_CD63.tif]

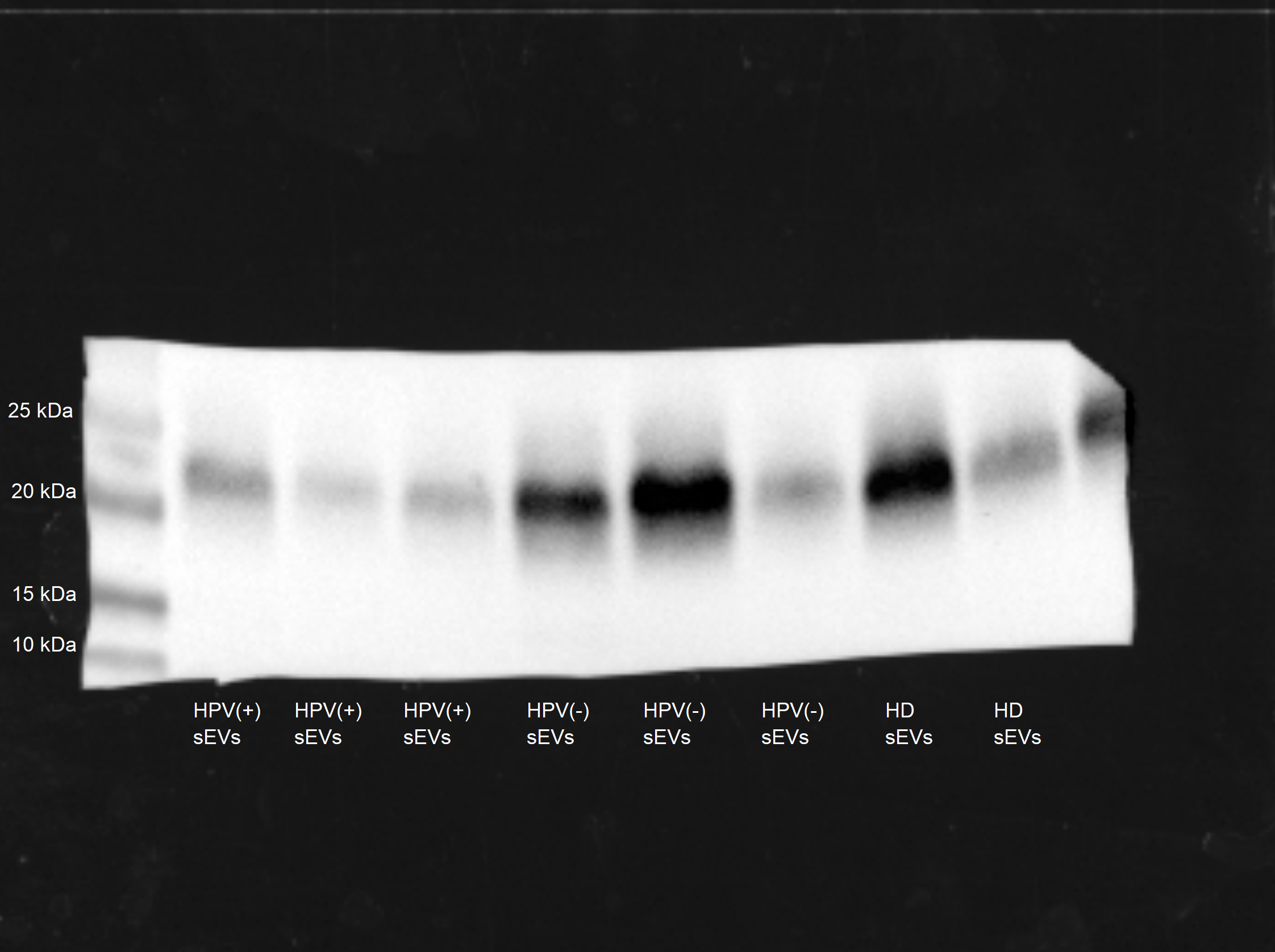

Supplement: Supplementary file 1 [file cancers-18-02219-s001.zip › supplement_proteomics_WB/full_WB_images_and_data/Fig1D_Plasma_sEVs_CD9.tif]

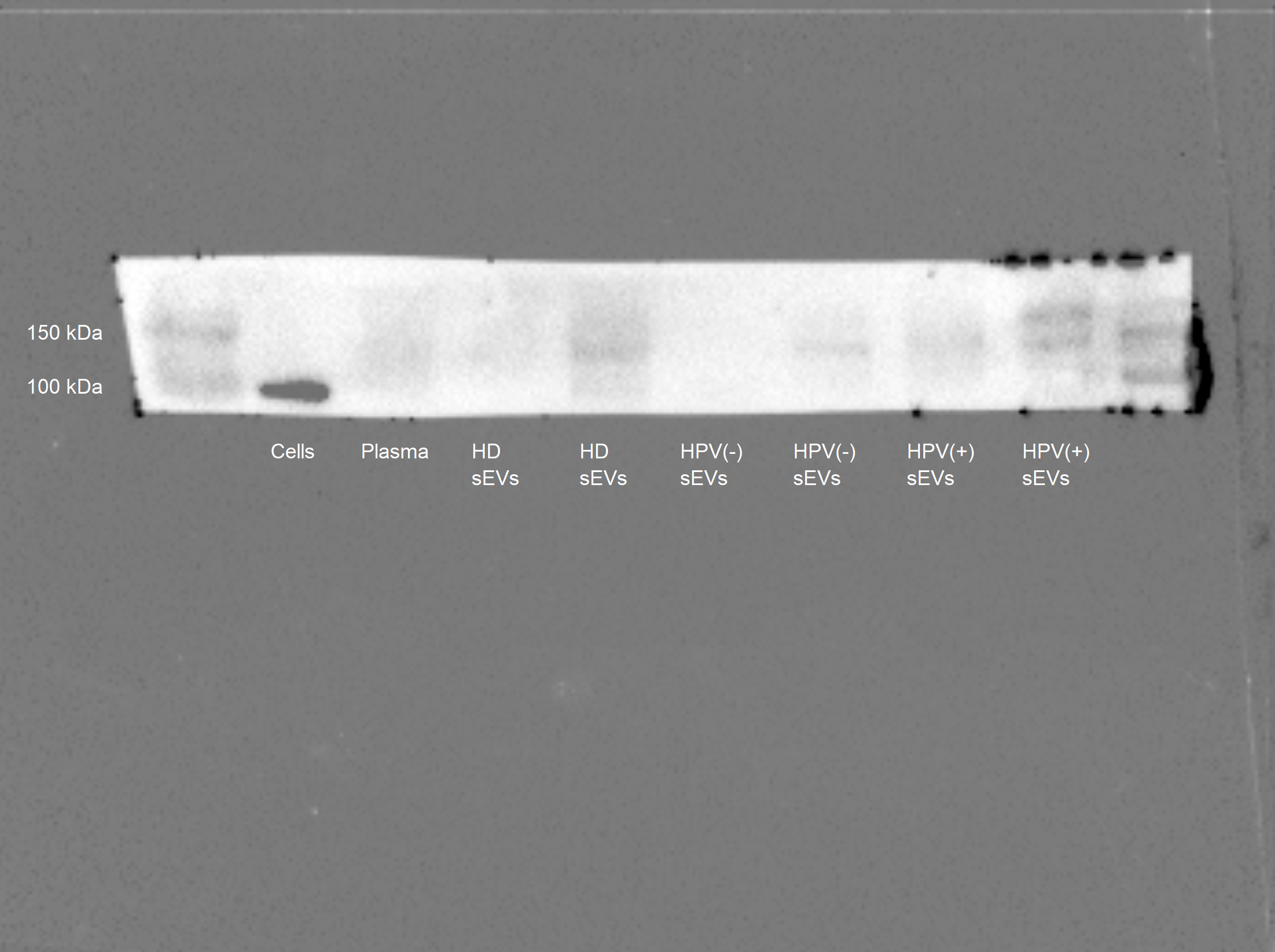

Supplement: Supplementary file 1 [file cancers-18-02219-s001.zip › supplement_proteomics_WB/full_WB_images_and_data/Fig1D_Plasma_sEVs_Grp94.tif]

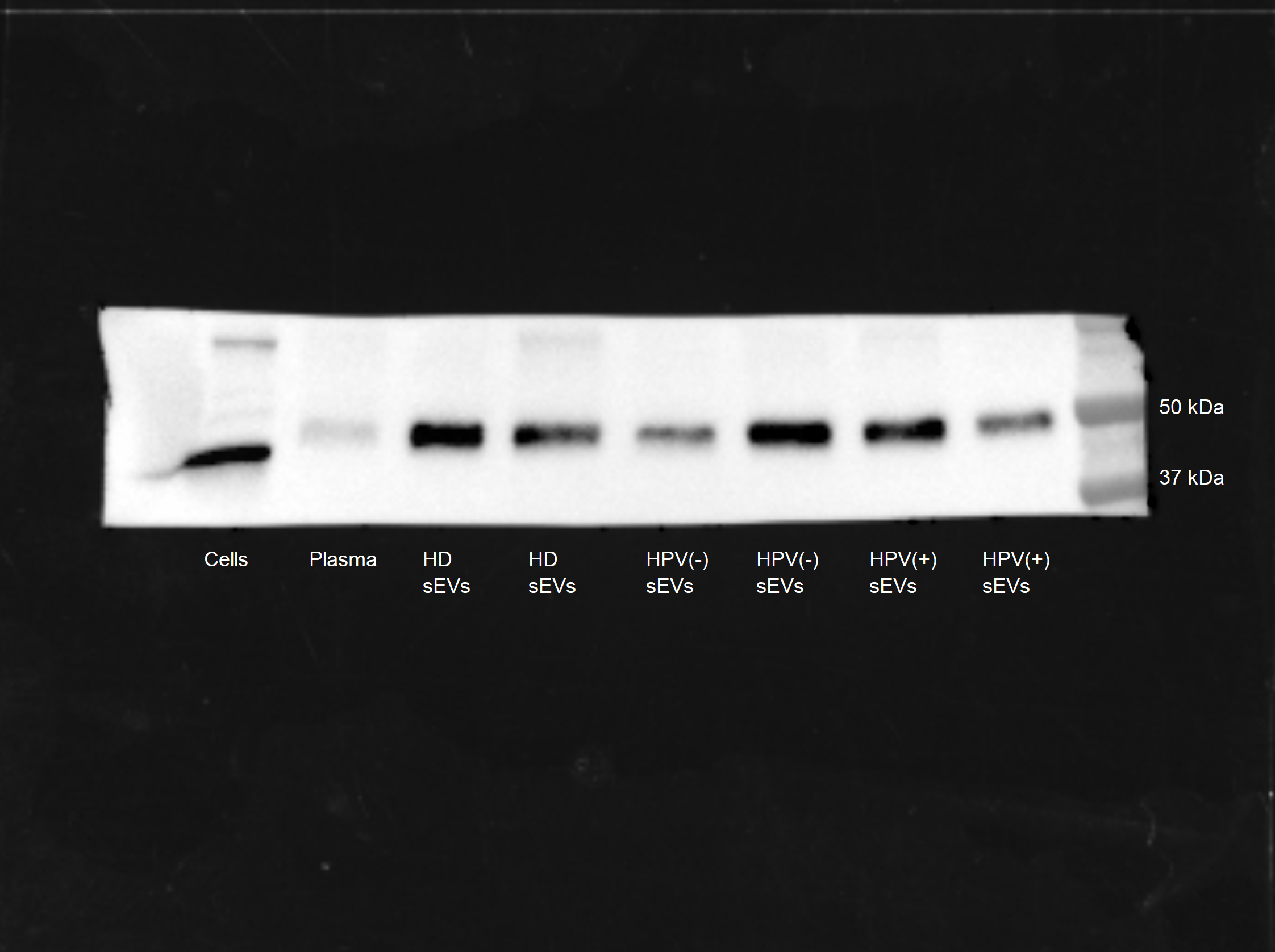

Supplement: Supplementary file 1 [file cancers-18-02219-s001.zip › supplement_proteomics_WB/full_WB_images_and_data/Fig1D_Plasma_sEVs_TSG101.tif]

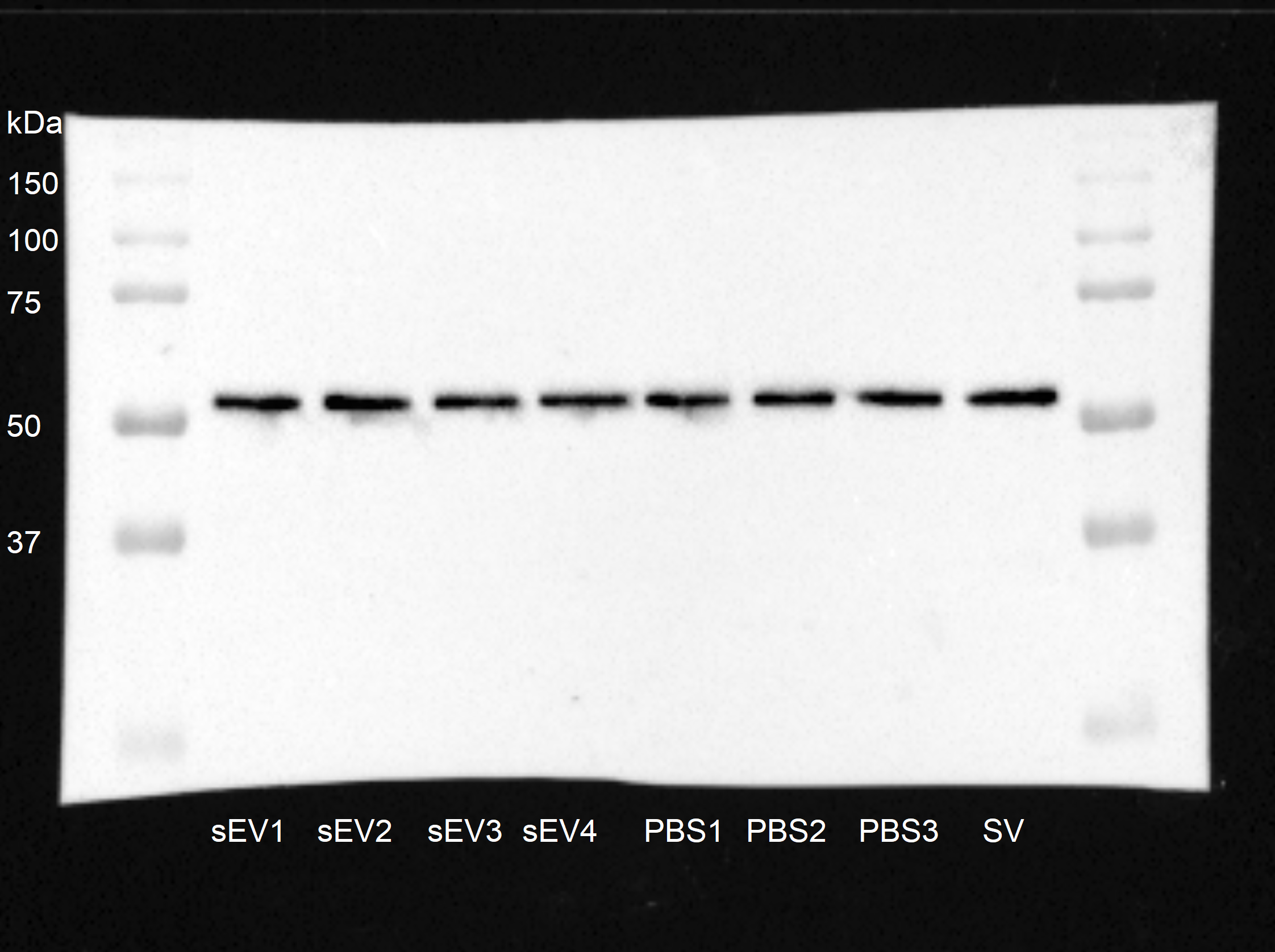

Supplement: Supplementary file 1 [file cancers-18-02219-s001.zip › supplement_proteomics_WB/full_WB_images_and_data/Fig4B_1h_aTubulin_1.tif]

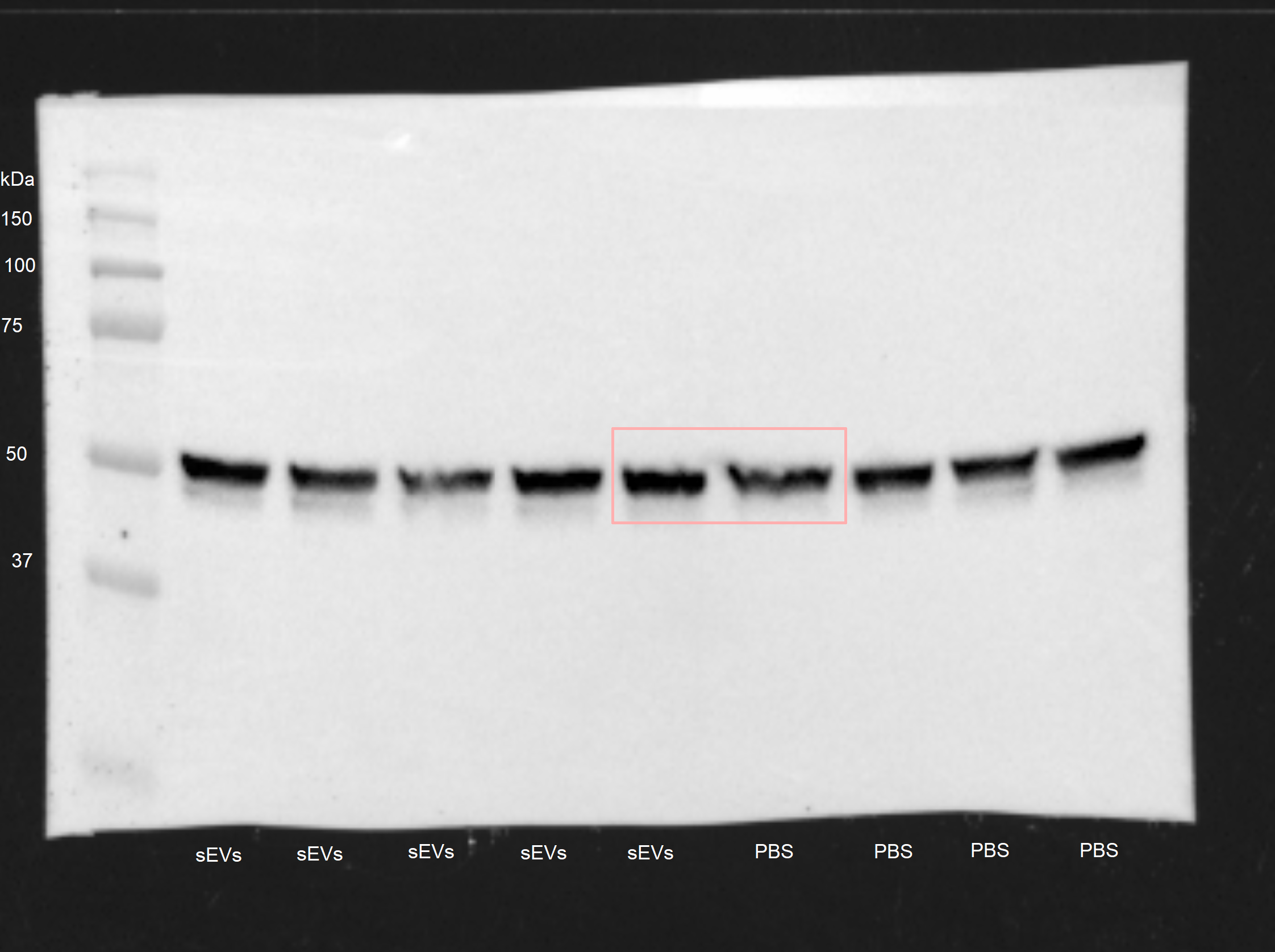

Supplement: Supplementary file 1 [file cancers-18-02219-s001.zip › supplement_proteomics_WB/full_WB_images_and_data/Fig4B_1h_aTubulin_for_IKBa_sEVs_PBS_2.tif]

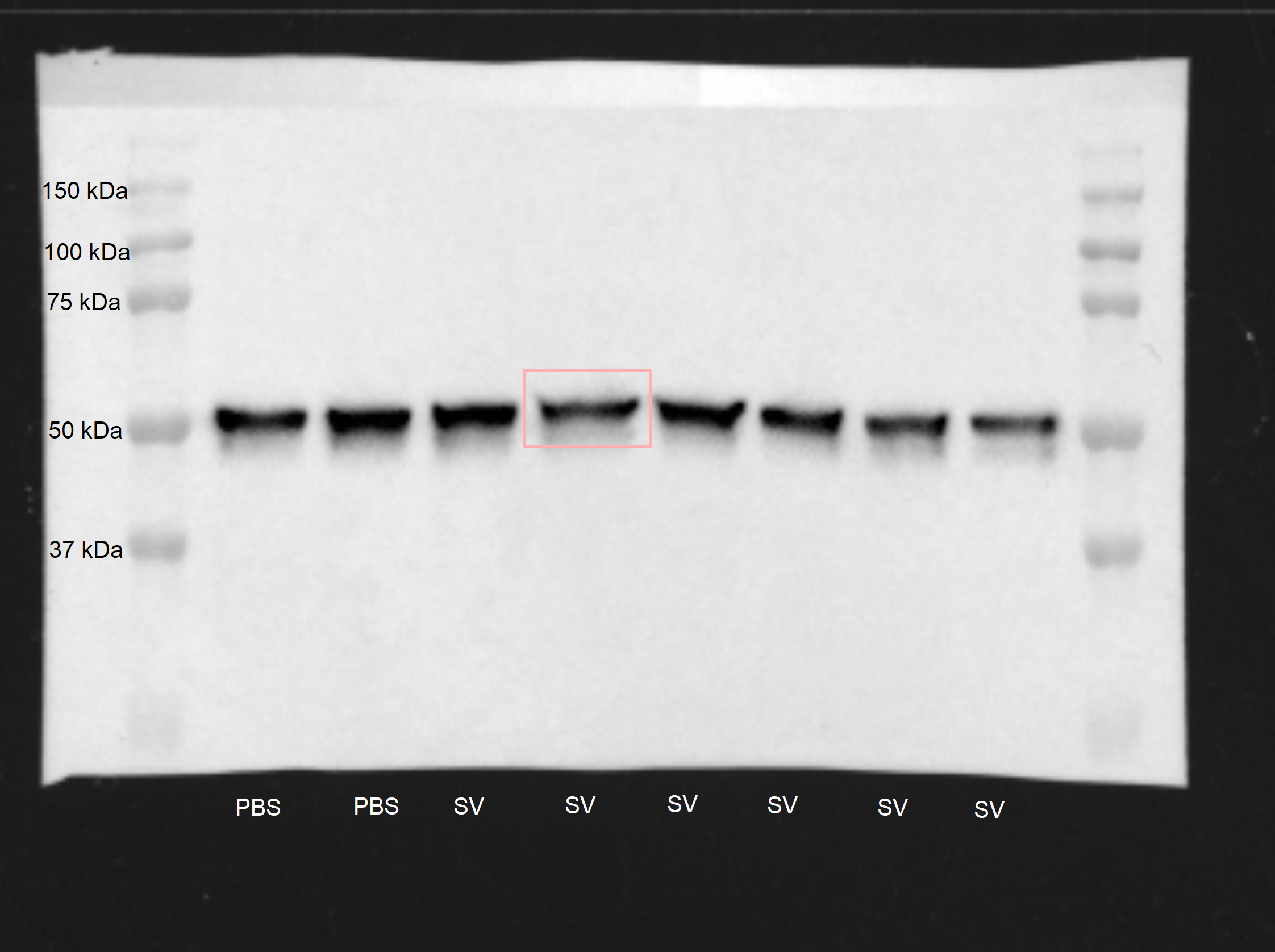

Supplement: Supplementary file 1 [file cancers-18-02219-s001.zip › supplement_proteomics_WB/full_WB_images_and_data/Fig4B_1h_aTubulin_for_IKBa_SV_2.tif]

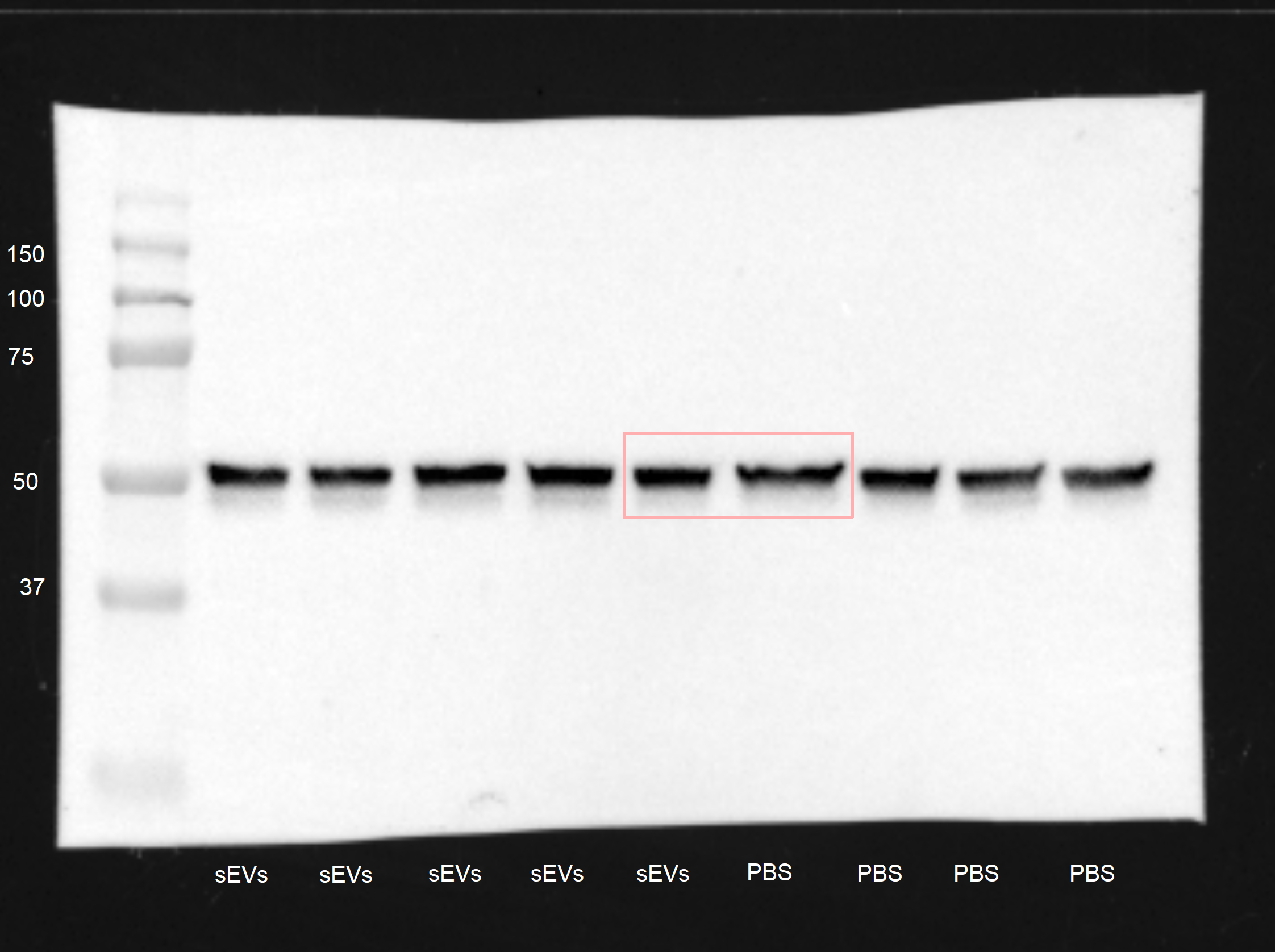

Supplement: Supplementary file 1 [file cancers-18-02219-s001.zip › supplement_proteomics_WB/full_WB_images_and_data/Fig4B_1h_aTubulin_for_pIKBa_sEVs_PBS_2.tif]

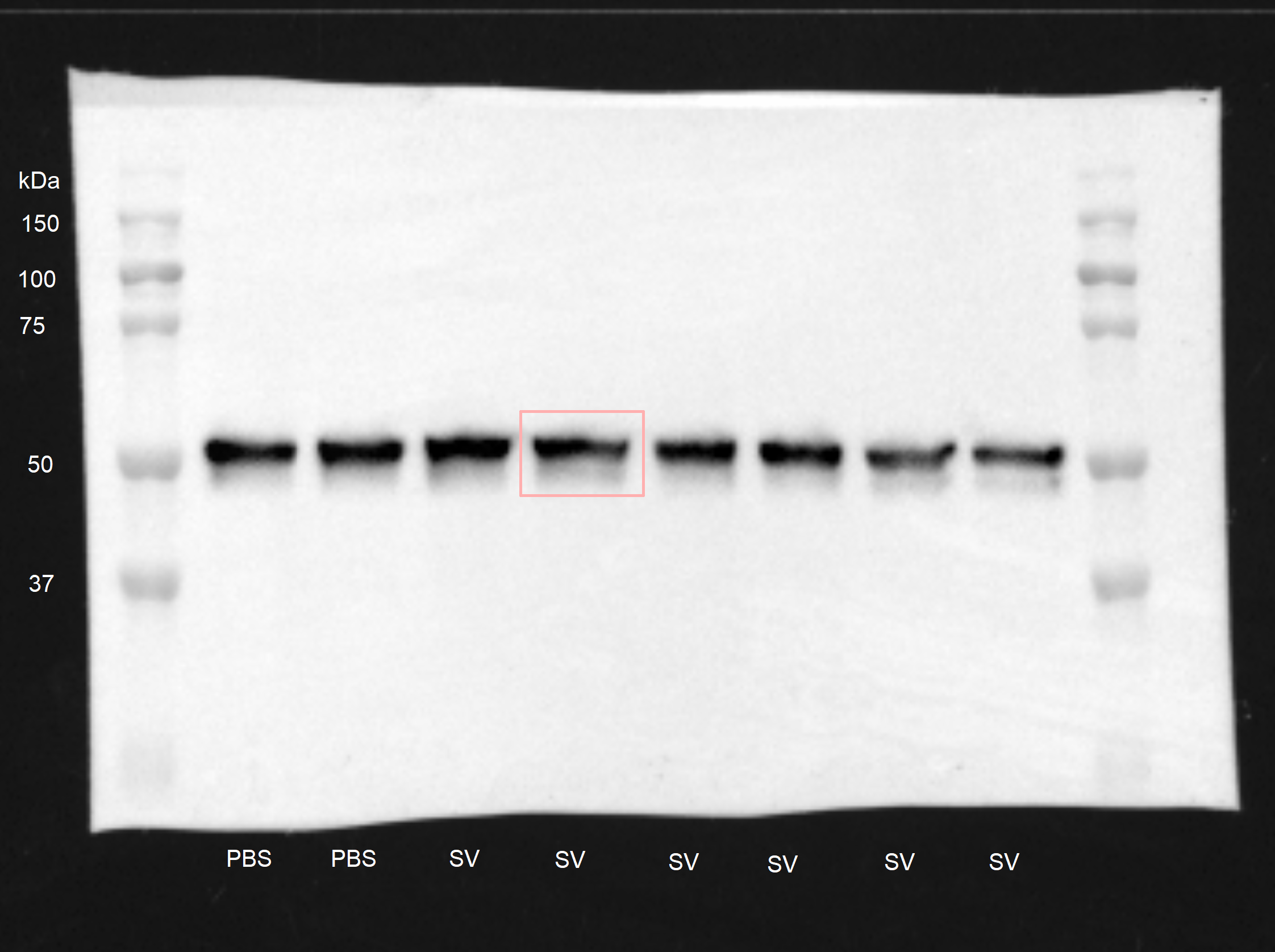

Supplement: Supplementary file 1 [file cancers-18-02219-s001.zip › supplement_proteomics_WB/full_WB_images_and_data/Fig4B_1h_aTubulin_for_pIKBa_SV_2.tif]

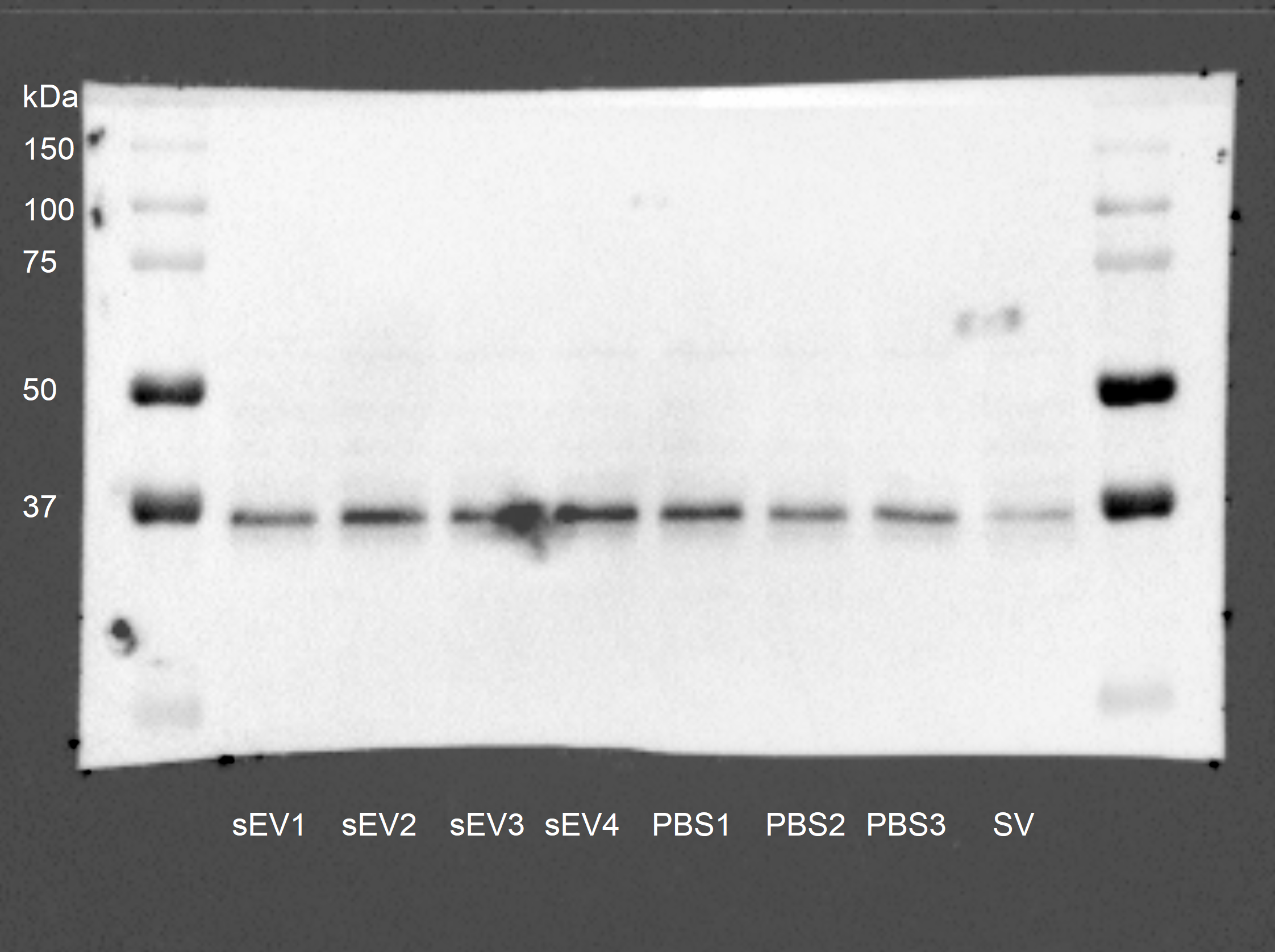

Supplement: Supplementary file 1 [file cancers-18-02219-s001.zip › supplement_proteomics_WB/full_WB_images_and_data/Fig4B_1h_IKBa_1.tif]

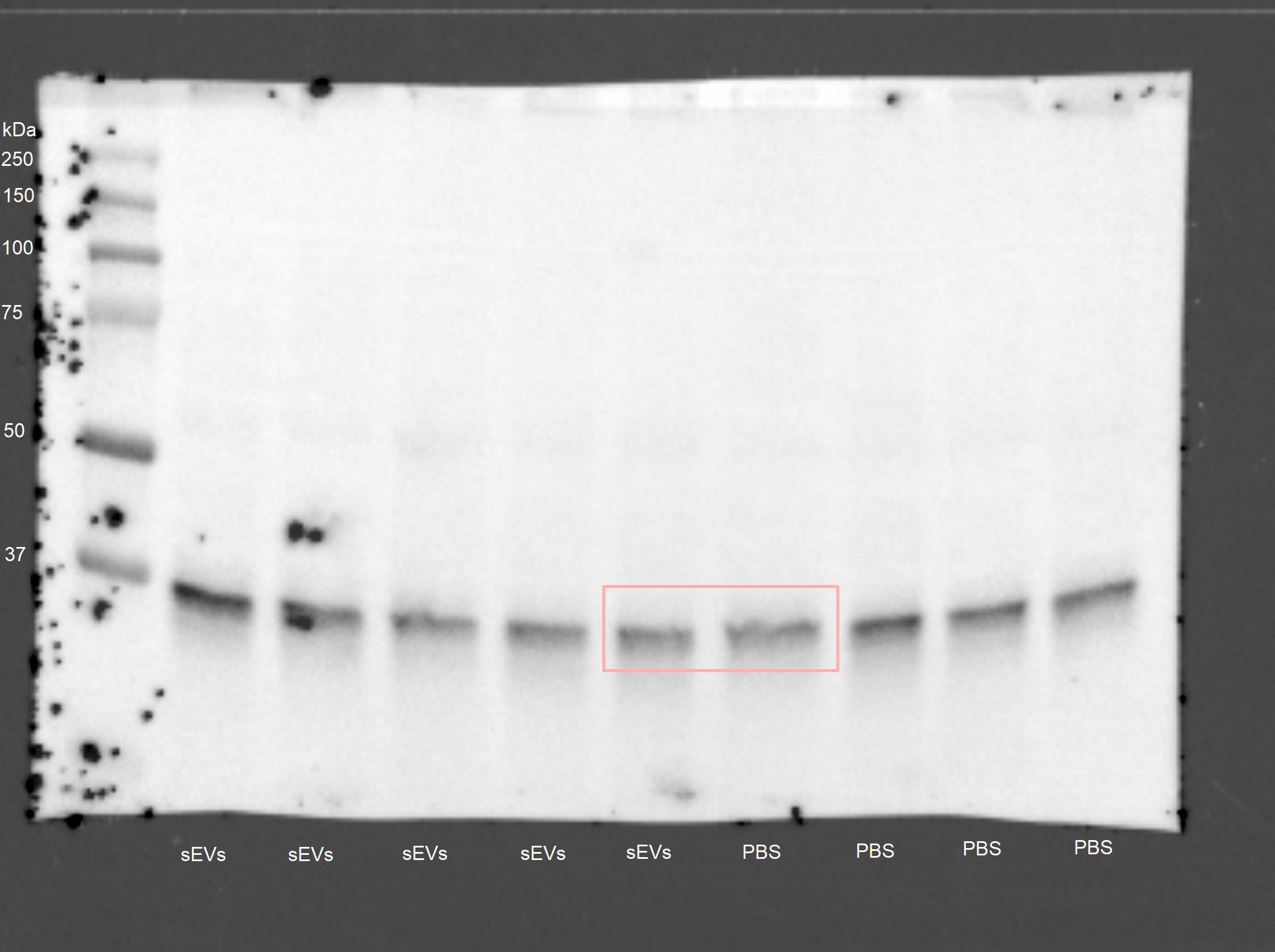

Supplement: Supplementary file 1 [file cancers-18-02219-s001.zip › supplement_proteomics_WB/full_WB_images_and_data/Fig4B_1h_IKBa_sEVs_PBS_2.tif]

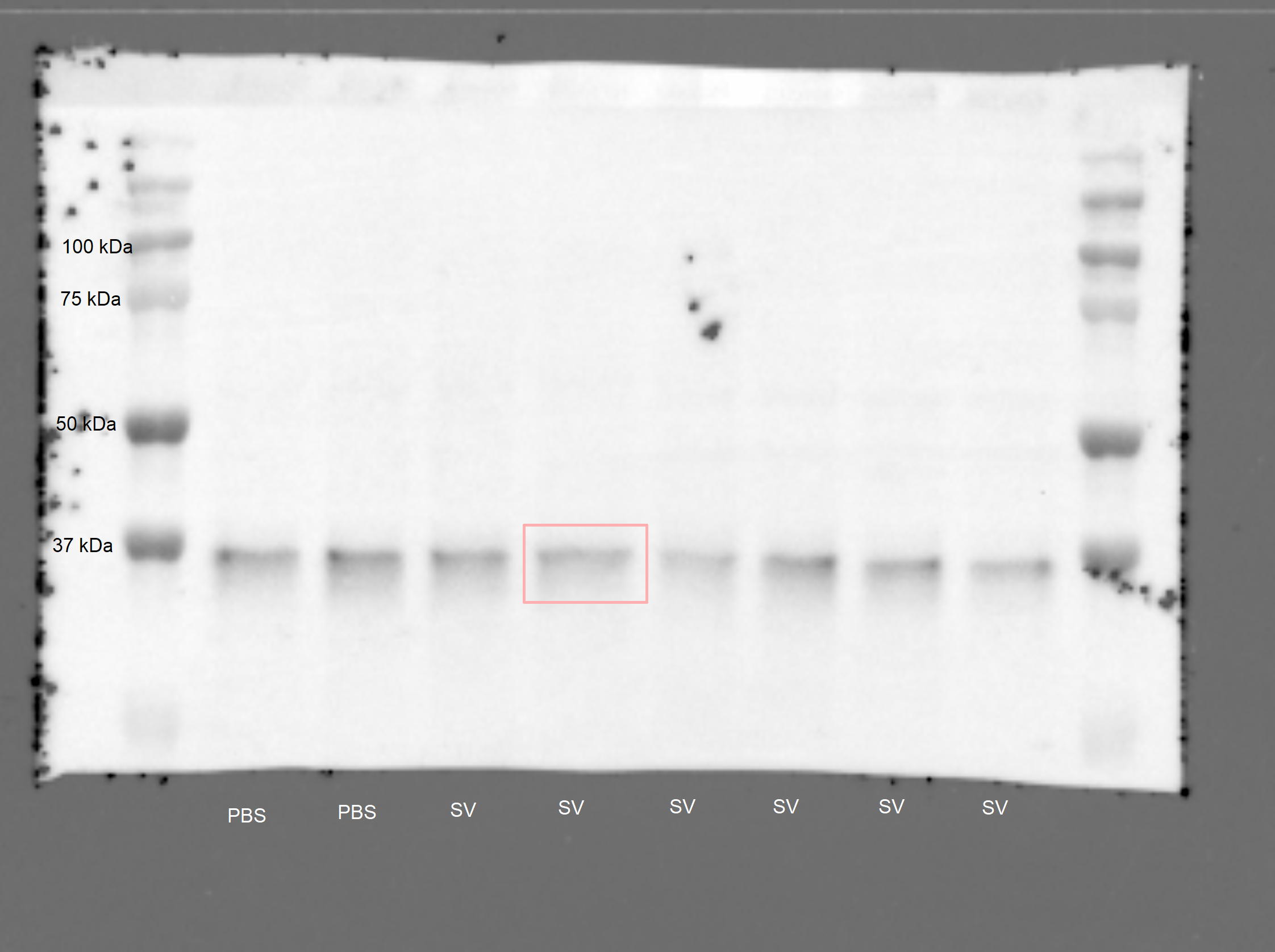

Supplement: Supplementary file 1 [file cancers-18-02219-s001.zip › supplement_proteomics_WB/full_WB_images_and_data/Fig4B_1h_IKBa_SV_2.tif]

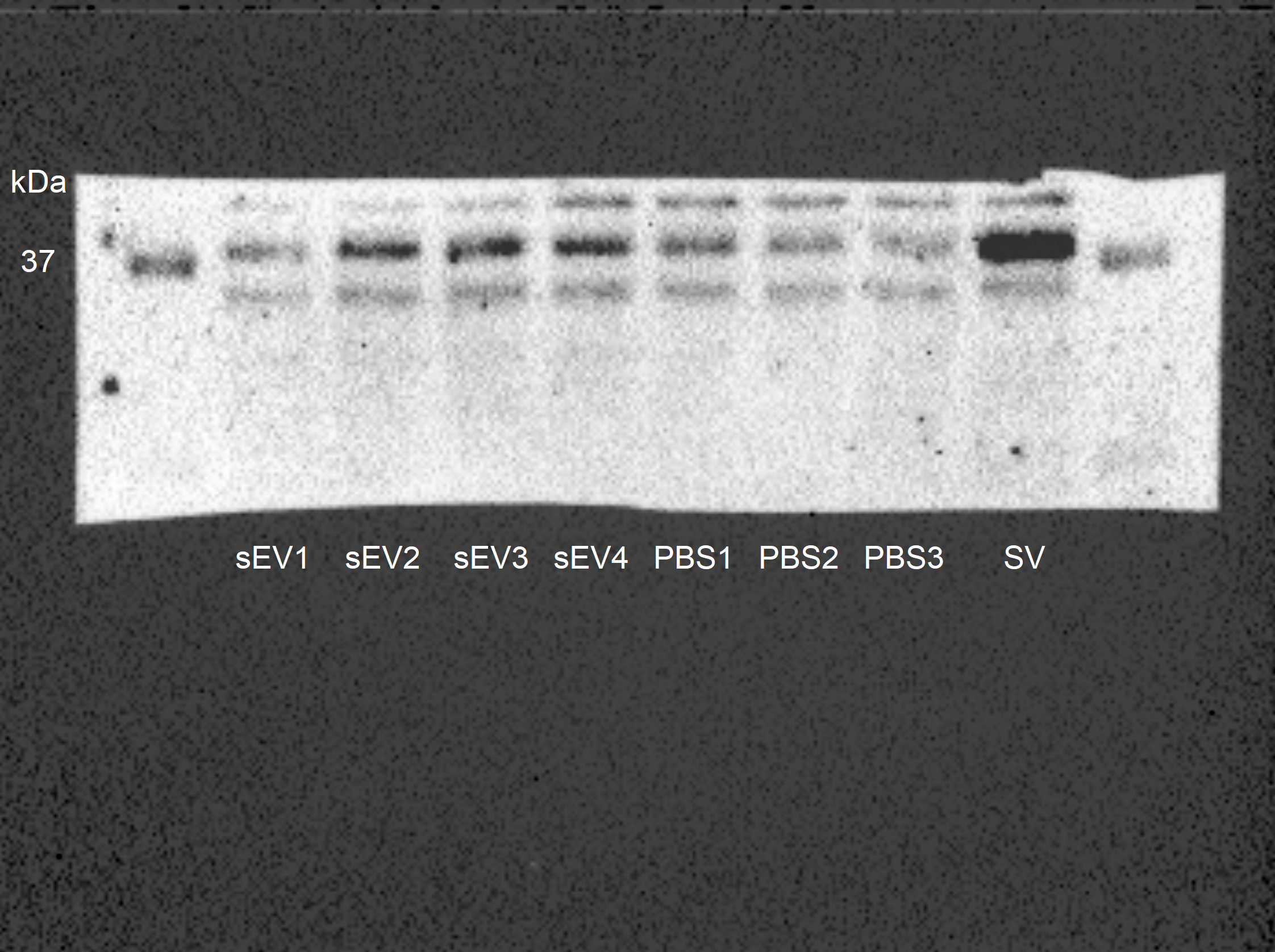

Supplement: Supplementary file 1 [file cancers-18-02219-s001.zip › supplement_proteomics_WB/full_WB_images_and_data/Fig4B_1h_pIKBa_1.tif]

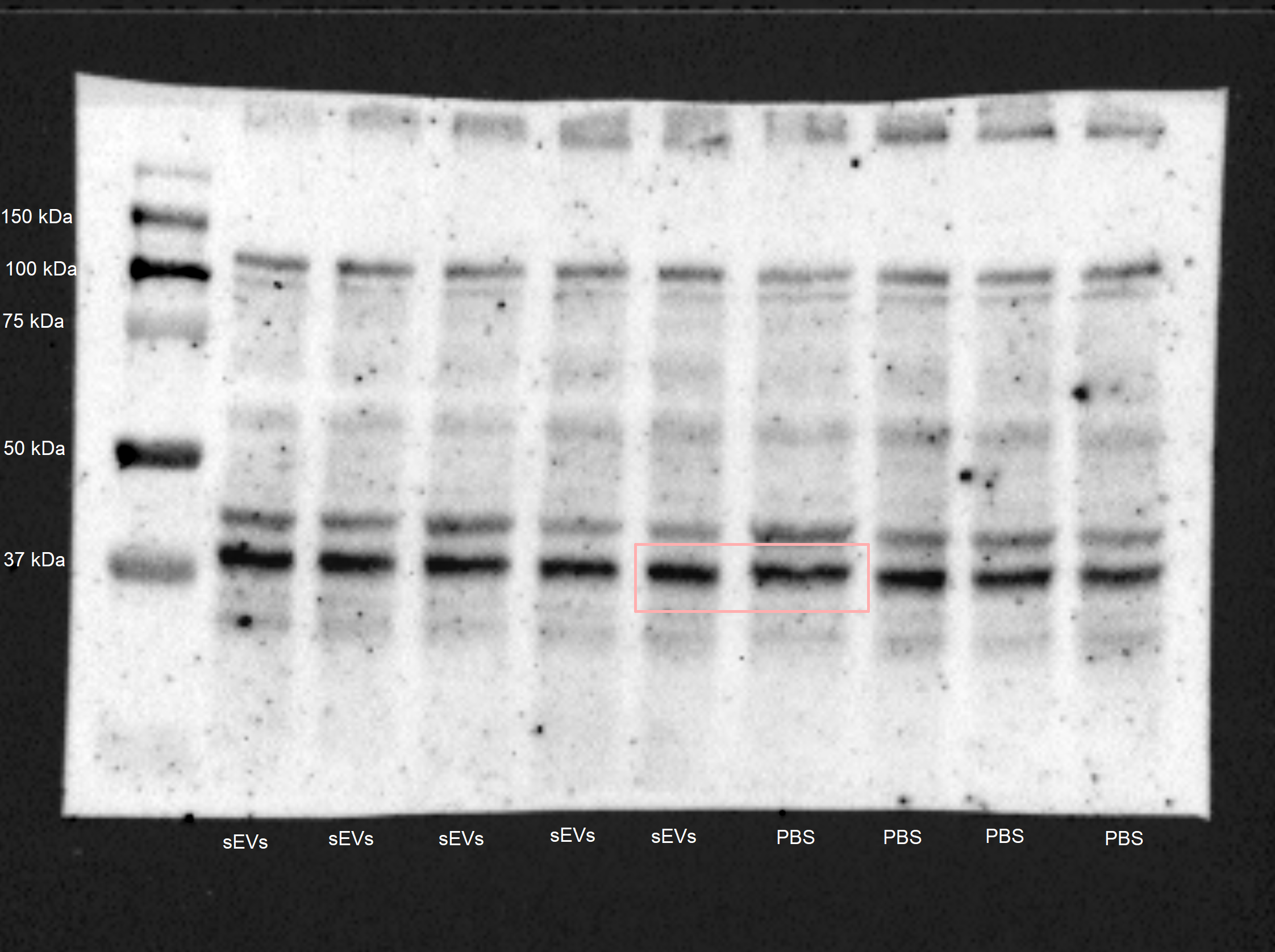

Supplement: Supplementary file 1 [file cancers-18-02219-s001.zip › supplement_proteomics_WB/full_WB_images_and_data/Fig4B_1h_pIKBa_sEVs_PBS_2.tif]

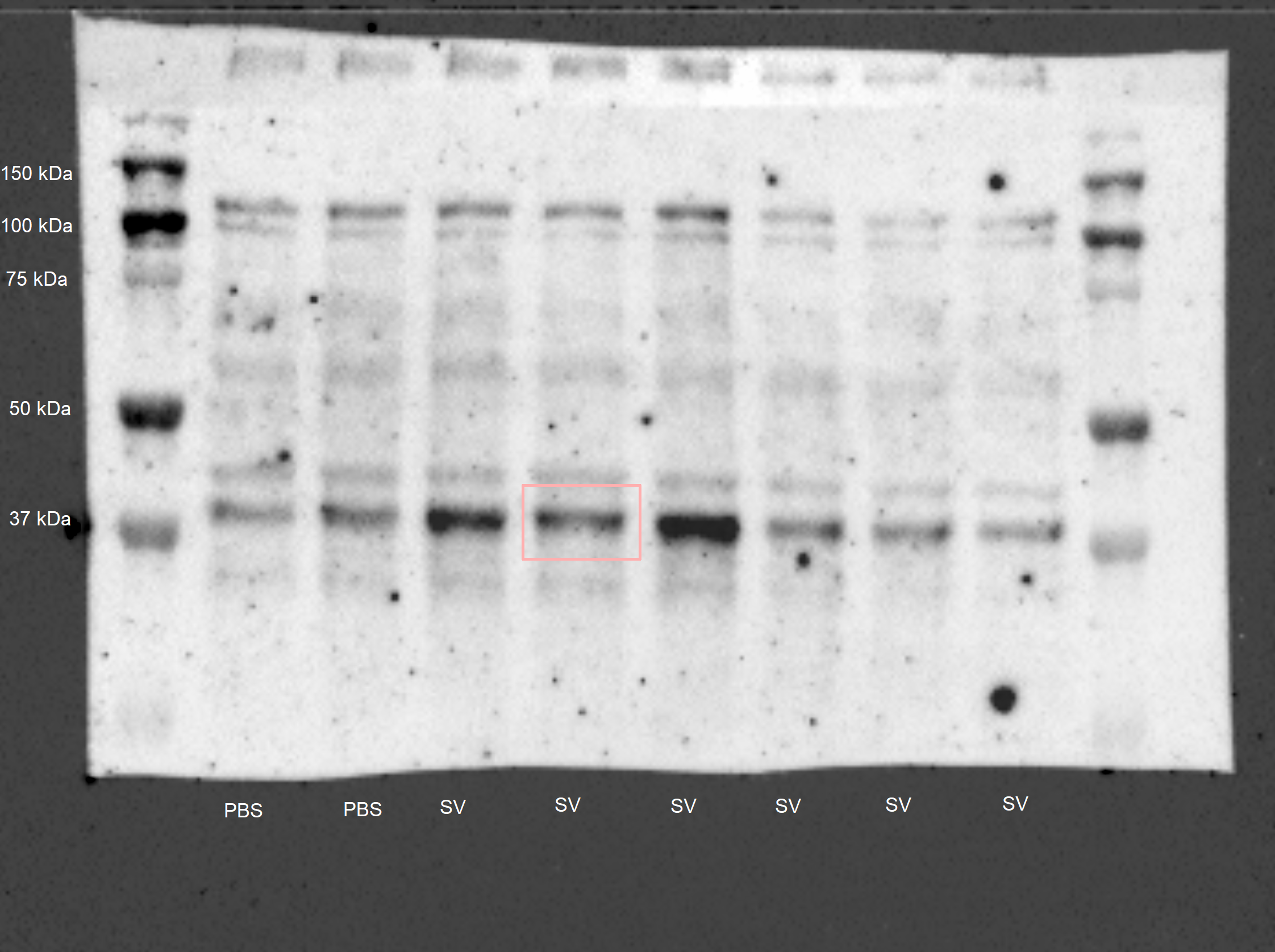

Supplement: Supplementary file 1 [file cancers-18-02219-s001.zip › supplement_proteomics_WB/full_WB_images_and_data/Fig4B_1h_pIKBa_SV_2.tif]

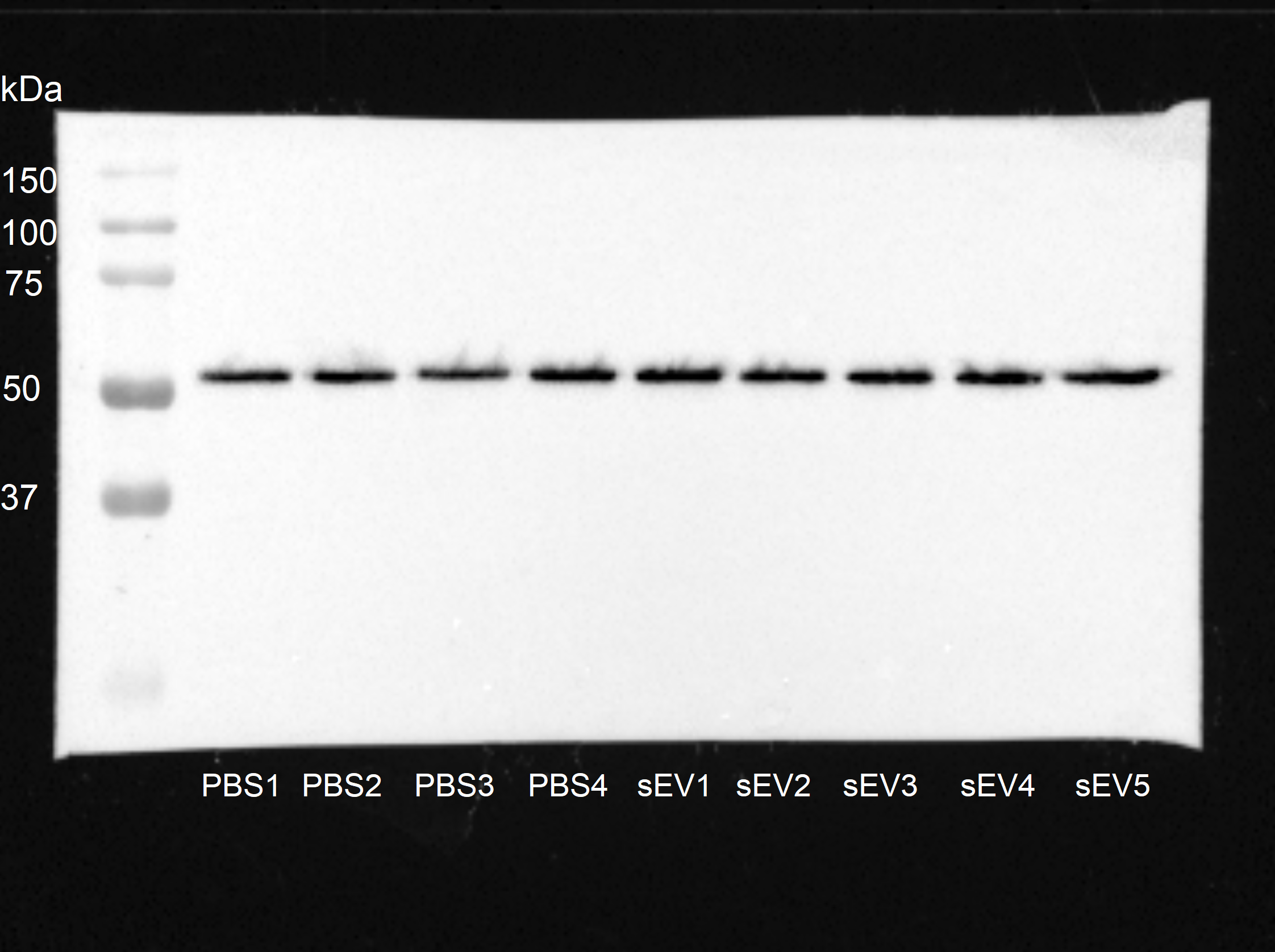

Supplement: Supplementary file 1 [file cancers-18-02219-s001.zip › supplement_proteomics_WB/full_WB_images_and_data/Fig4B_24h_aTubulin_1.tif]

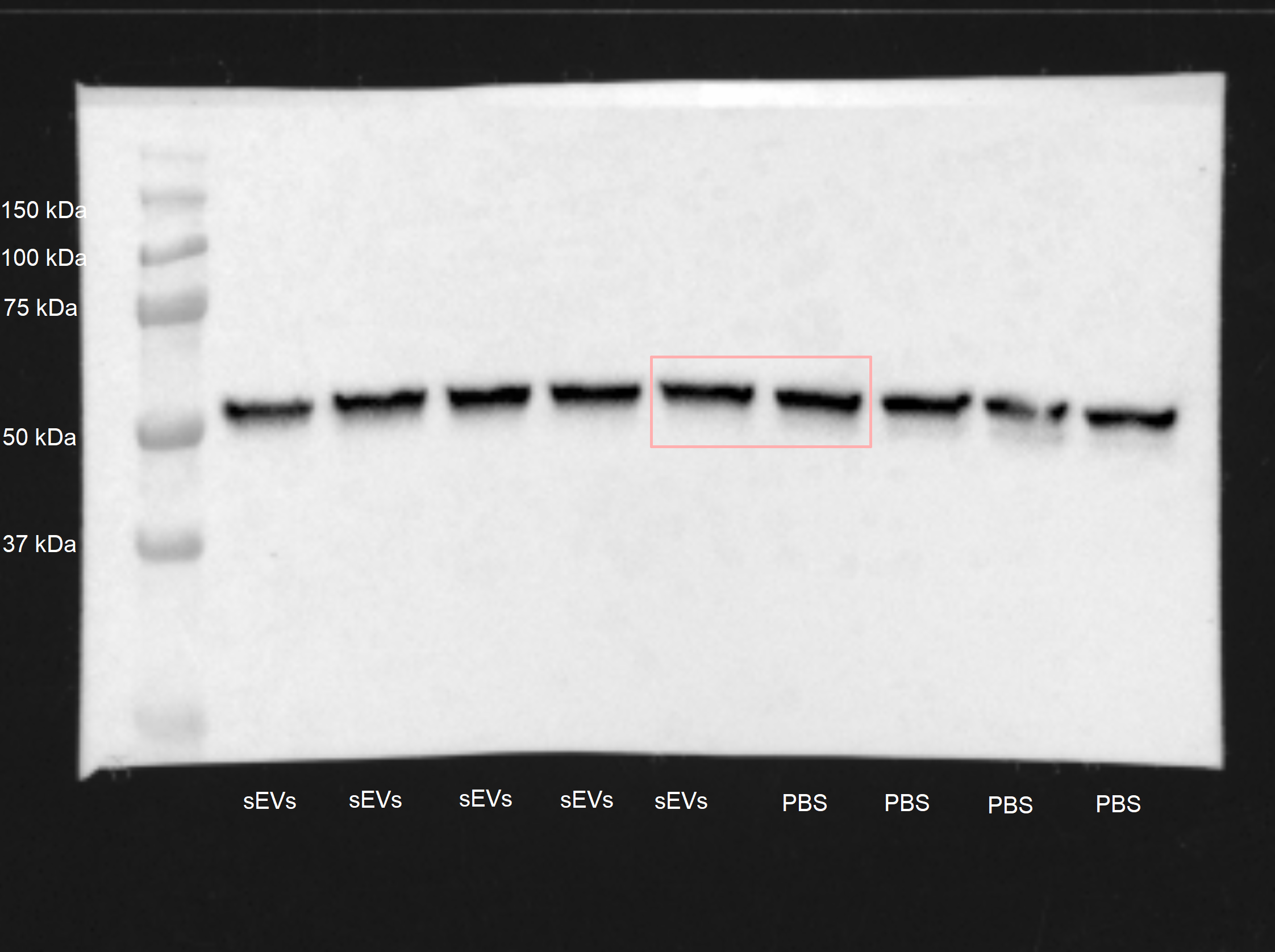

Supplement: Supplementary file 1 [file cancers-18-02219-s001.zip › supplement_proteomics_WB/full_WB_images_and_data/Fig4B_24h_aTubulin_for_IKBa_sEVs_PBS_2.tif]

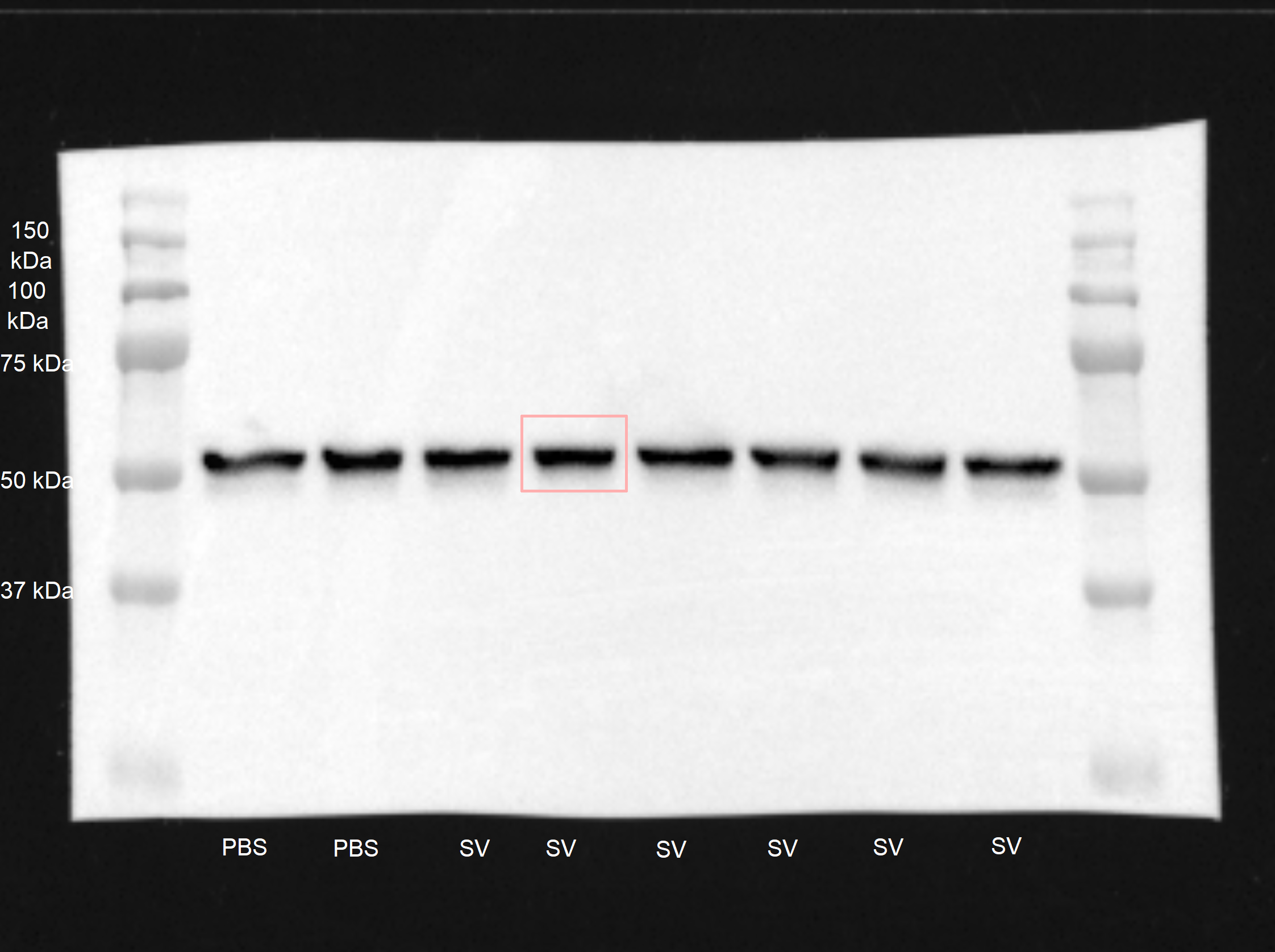

Supplement: Supplementary file 1 [file cancers-18-02219-s001.zip › supplement_proteomics_WB/full_WB_images_and_data/Fig4B_24h_aTubulin_for_IKBa_SV_2.tif]

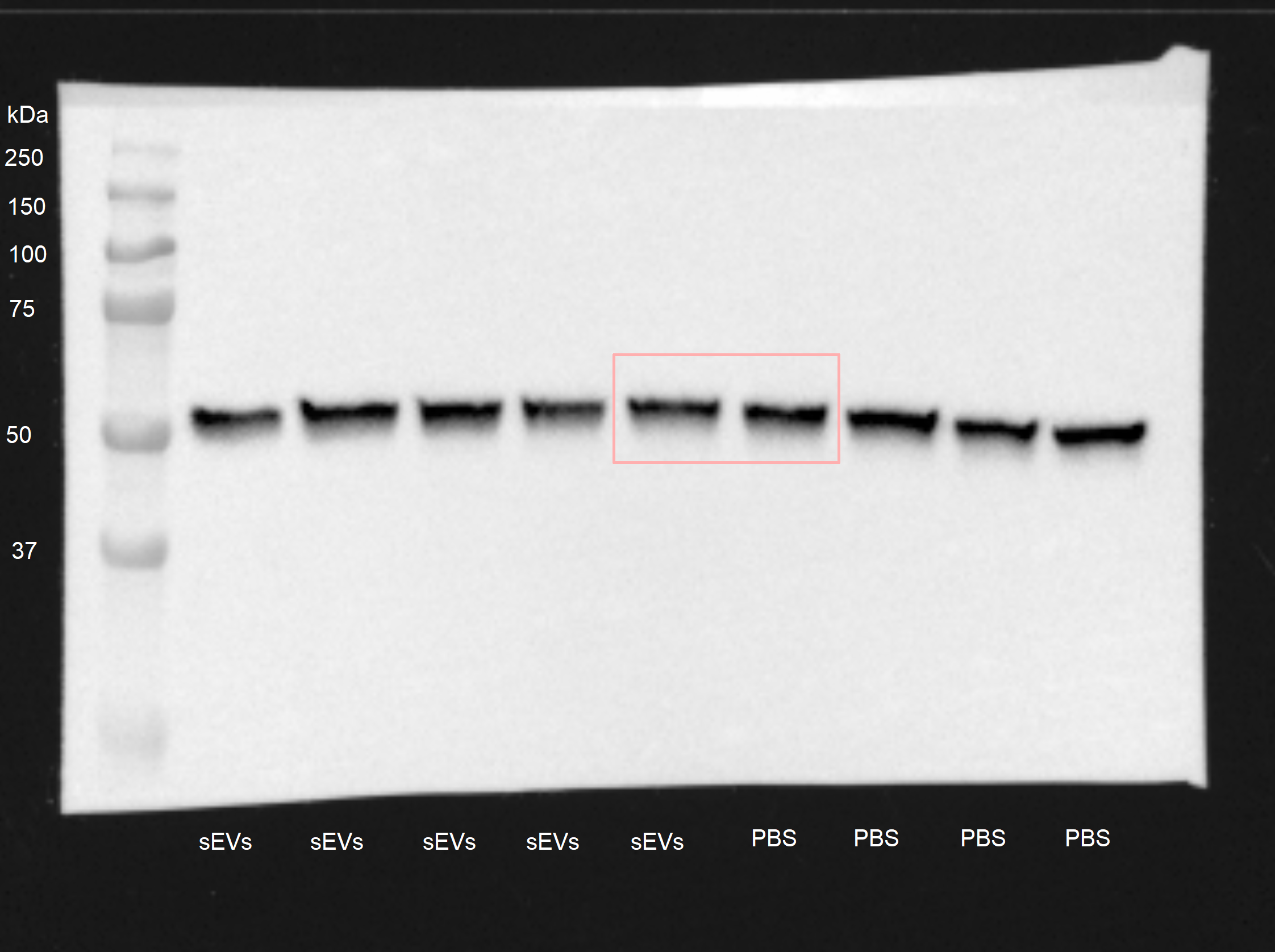

Supplement: Supplementary file 1 [file cancers-18-02219-s001.zip › supplement_proteomics_WB/full_WB_images_and_data/Fig4B_24h_aTubulin_for_pIKBa_sEVs_PBS_2.tif]

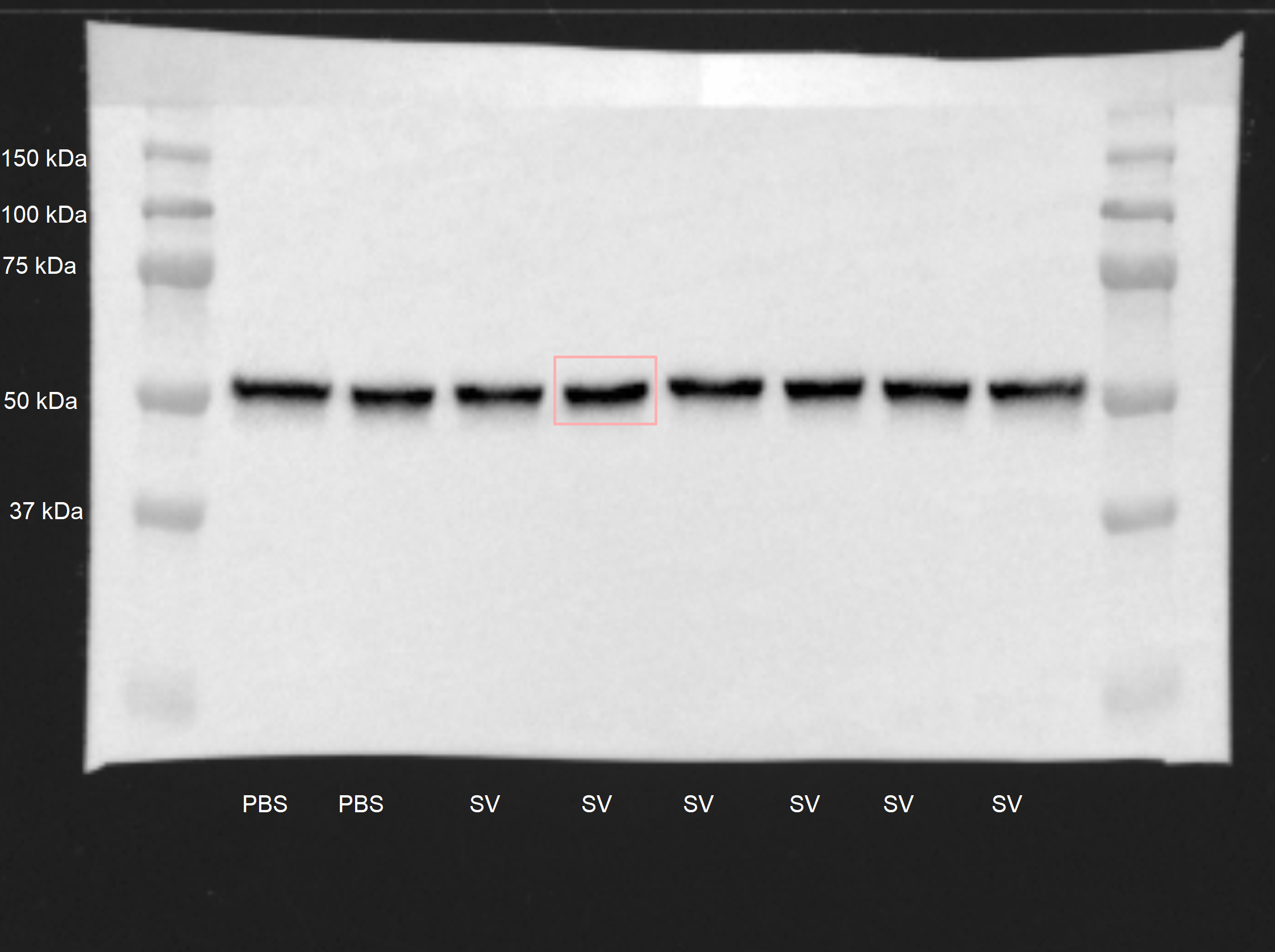

Supplement: Supplementary file 1 [file cancers-18-02219-s001.zip › supplement_proteomics_WB/full_WB_images_and_data/Fig4B_24h_aTubulin_for_pIKBa_SV_2.tif]

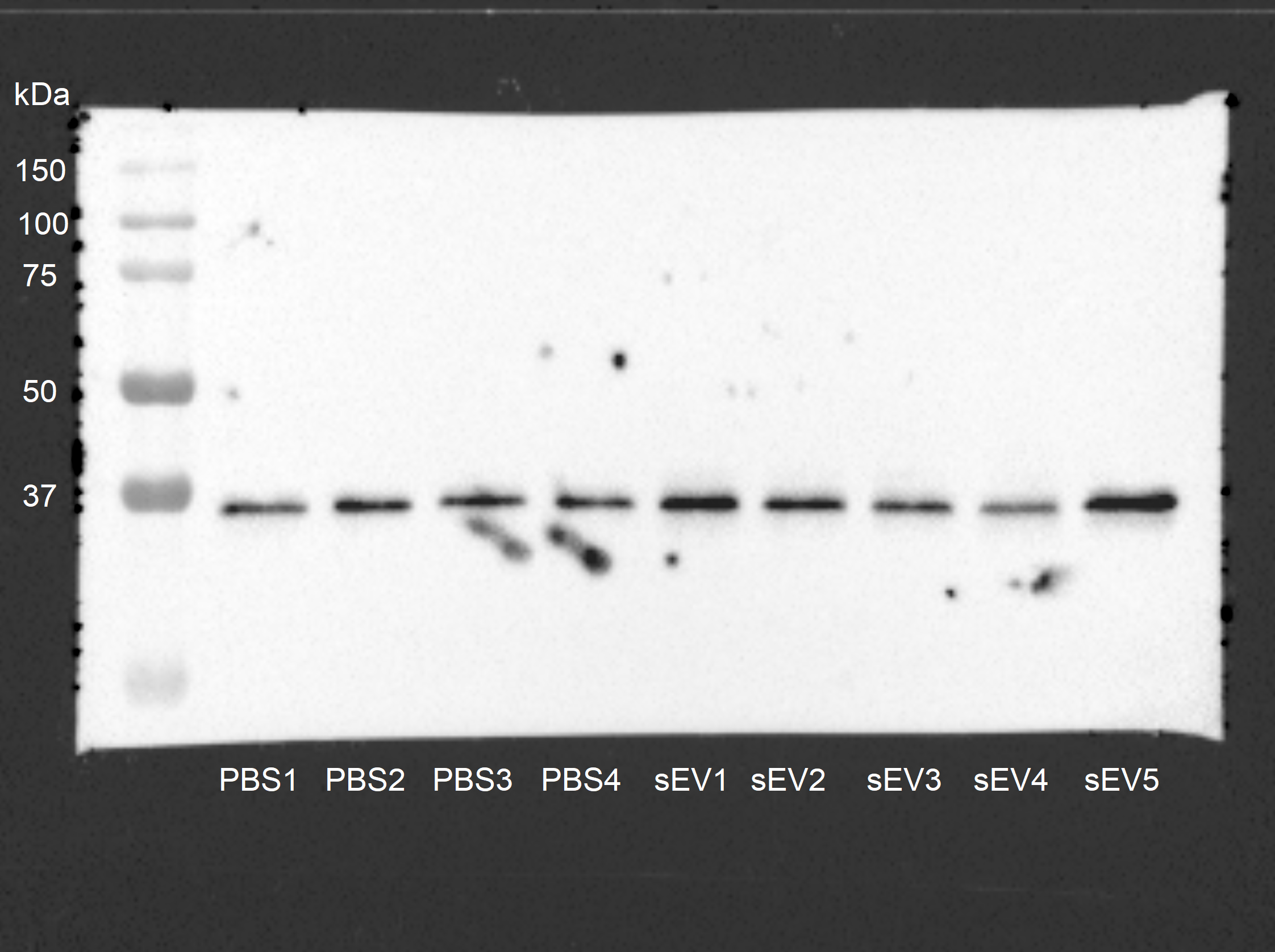

Supplement: Supplementary file 1 [file cancers-18-02219-s001.zip › supplement_proteomics_WB/full_WB_images_and_data/Fig4B_24h_IKBa_1.tif]

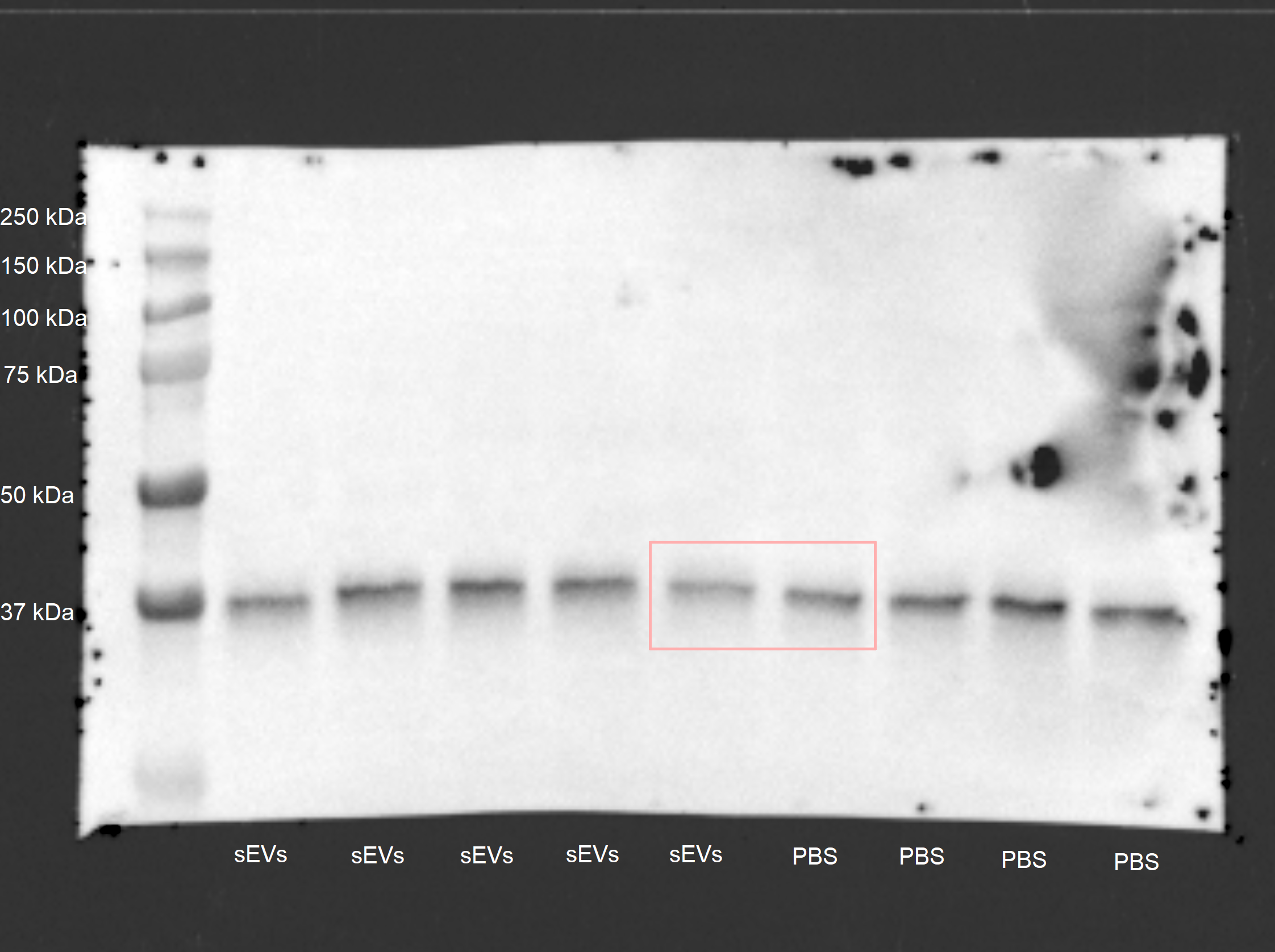

Supplement: Supplementary file 1 [file cancers-18-02219-s001.zip › supplement_proteomics_WB/full_WB_images_and_data/Fig4B_24h_IKBa_sEVs_PBS_2.tif]

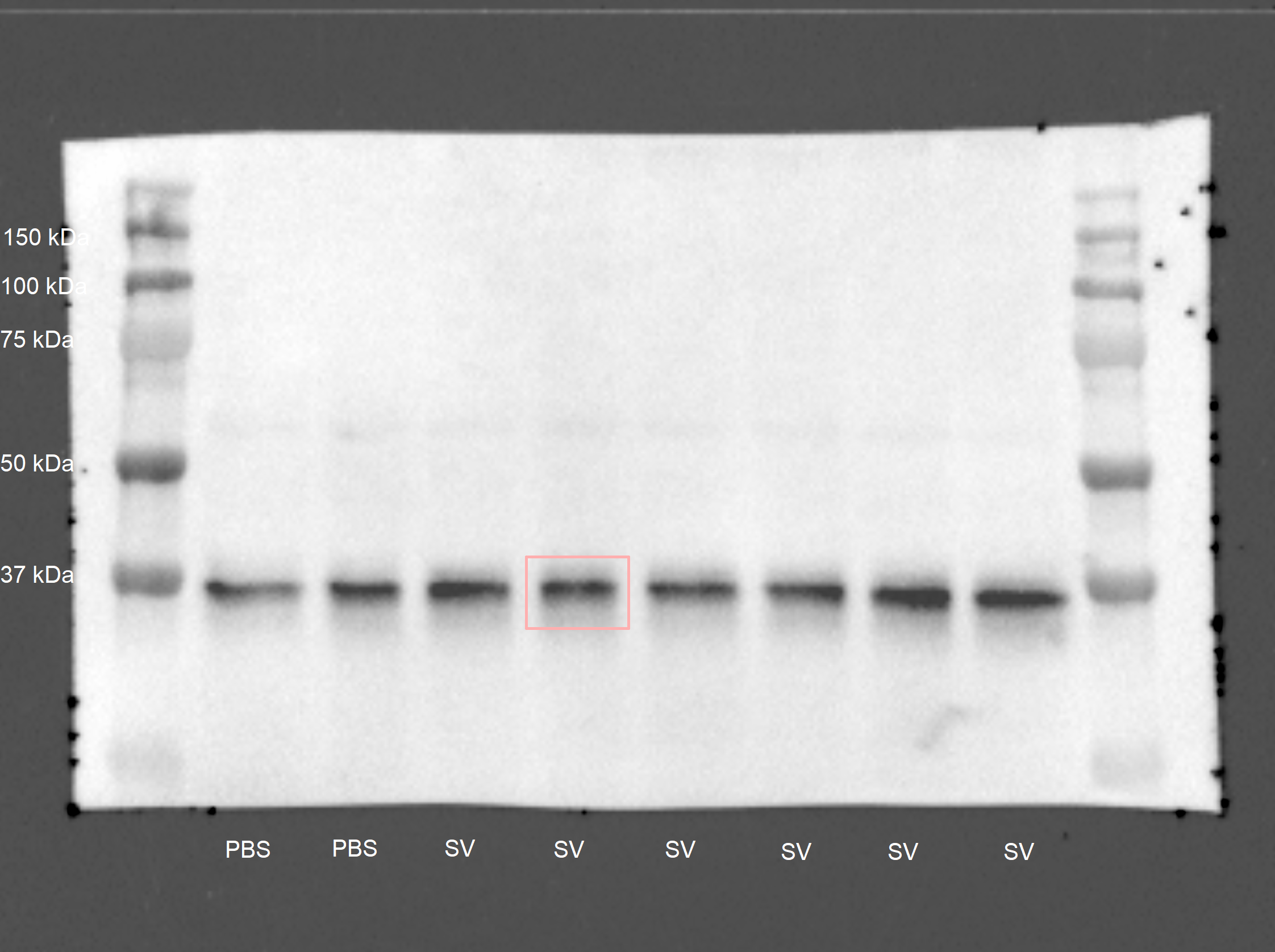

Supplement: Supplementary file 1 [file cancers-18-02219-s001.zip › supplement_proteomics_WB/full_WB_images_and_data/Fig4B_24h_IKBa_SV_2.tif]

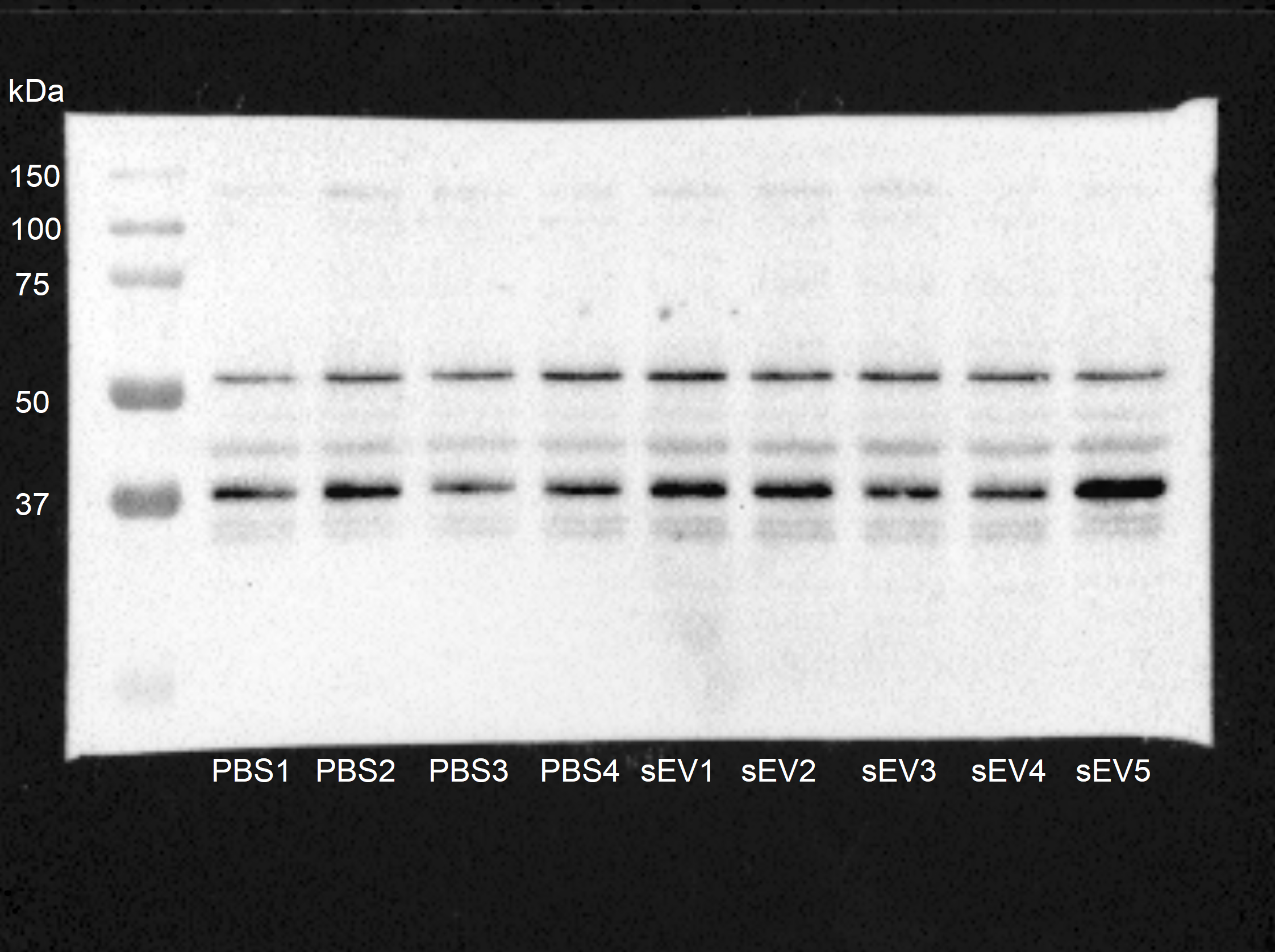

Supplement: Supplementary file 1 [file cancers-18-02219-s001.zip › supplement_proteomics_WB/full_WB_images_and_data/Fig4B_24h_pIKBa_1.tif]

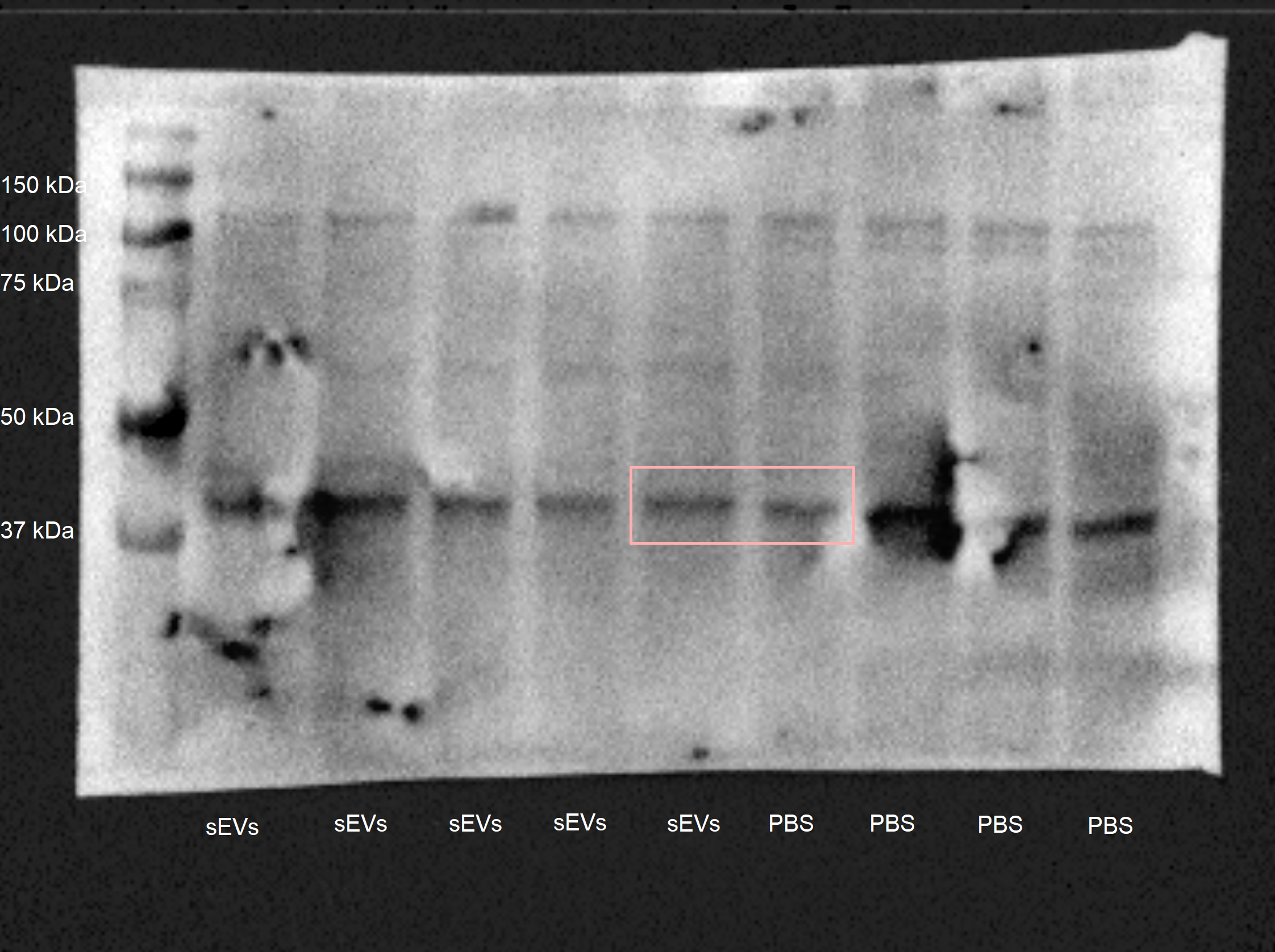

Supplement: Supplementary file 1 [file cancers-18-02219-s001.zip › supplement_proteomics_WB/full_WB_images_and_data/Fig4B_24h_pIKBa_sEVs_PBS_2.tif]

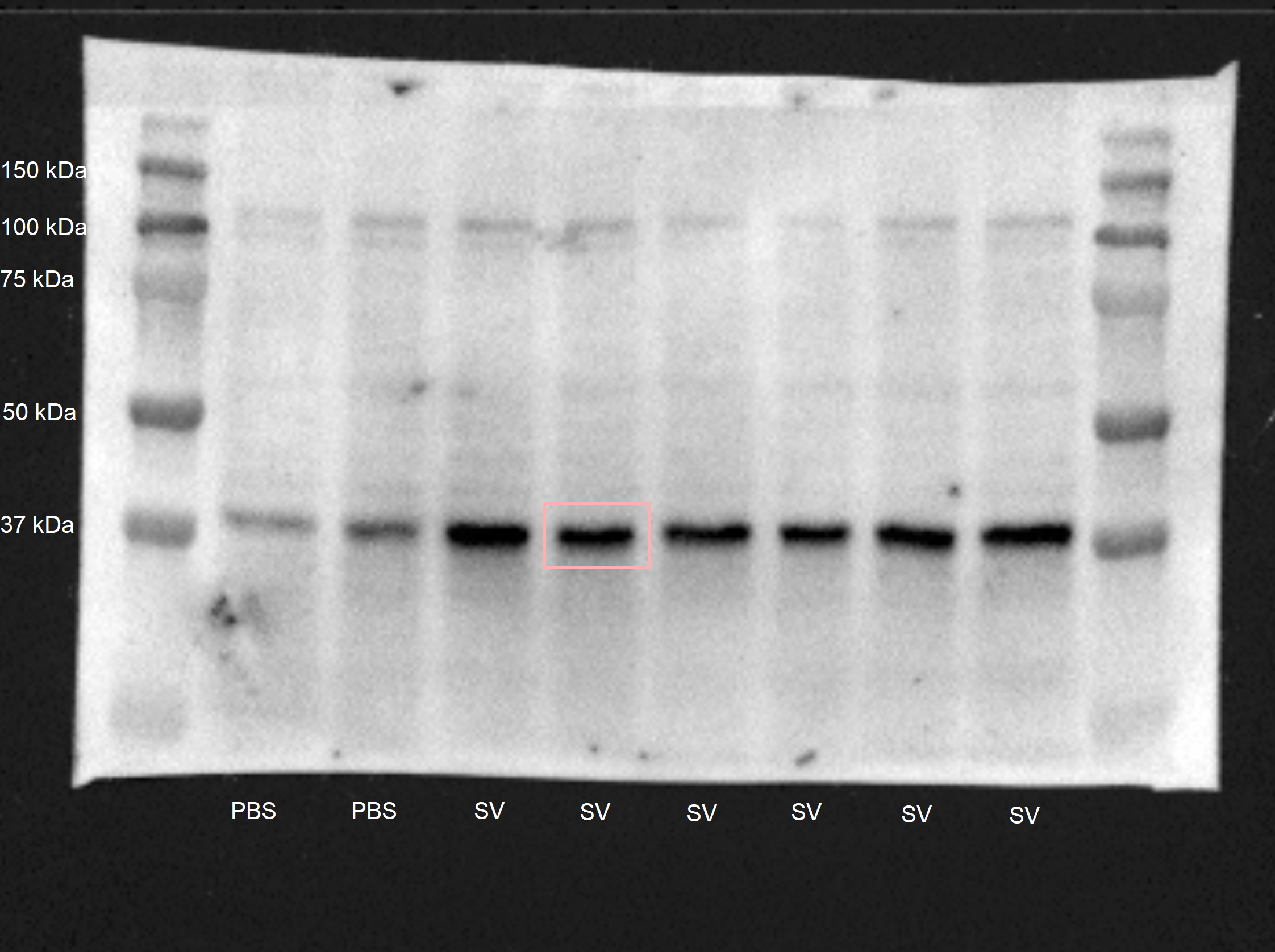

Supplement: Supplementary file 1 [file cancers-18-02219-s001.zip › supplement_proteomics_WB/full_WB_images_and_data/Fig4B_24h_pIKBa_SV_2.tif]

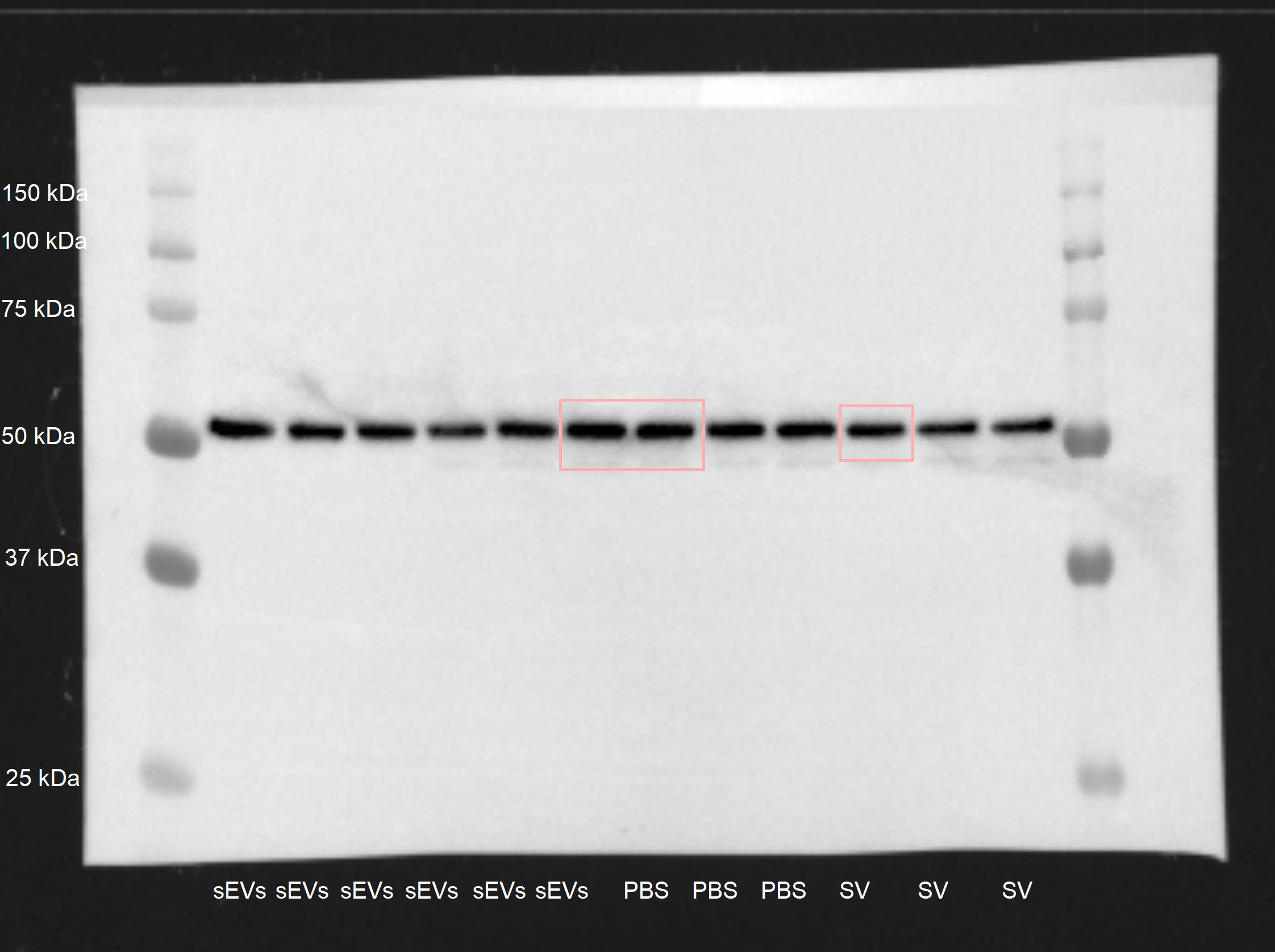

Supplement: Supplementary file 1 [file cancers-18-02219-s001.zip › supplement_proteomics_WB/full_WB_images_and_data/Fig4B_2h_aTubulin_for_IKBa_1.tif]

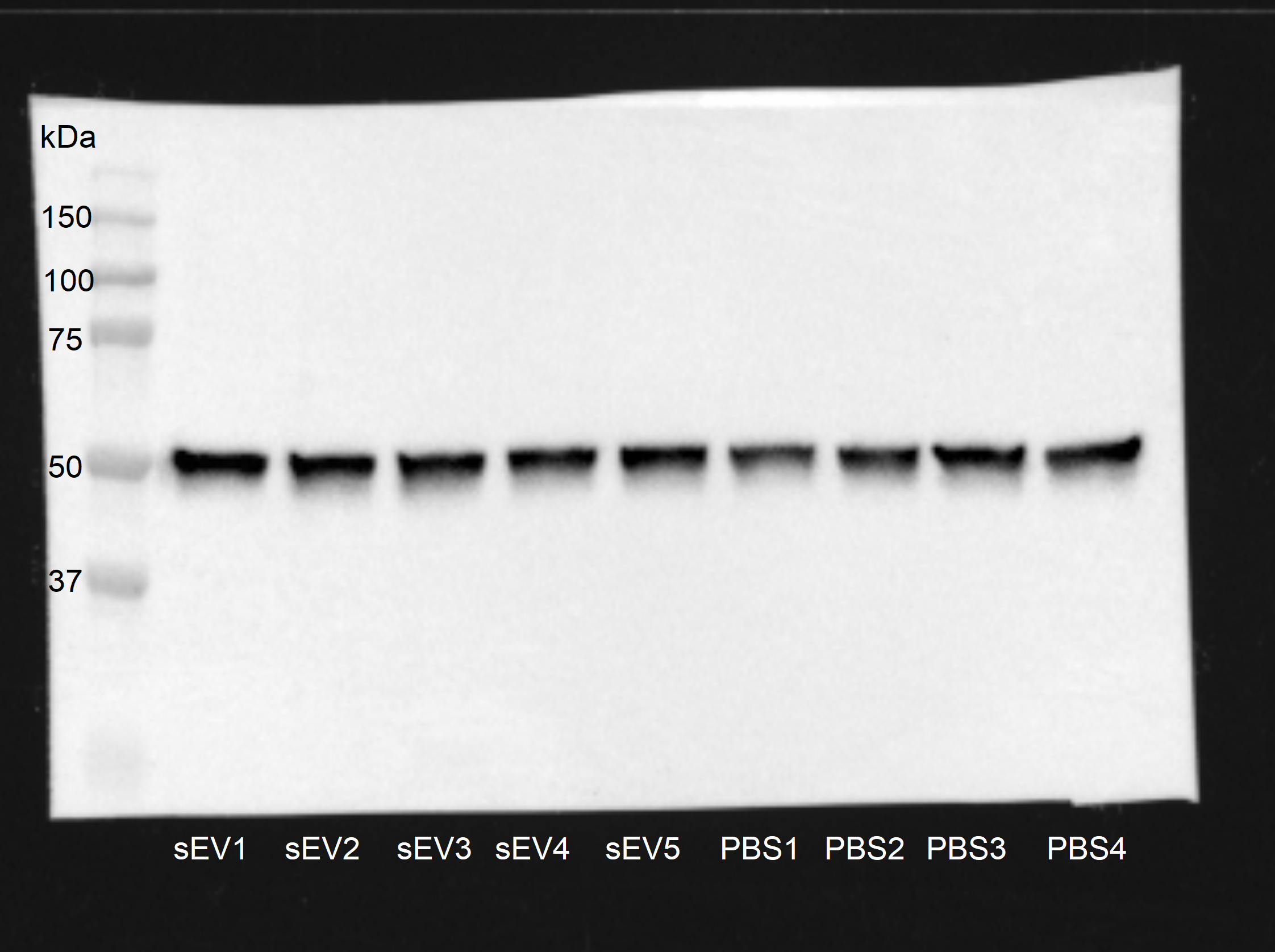

Supplement: Supplementary file 1 [file cancers-18-02219-s001.zip › supplement_proteomics_WB/full_WB_images_and_data/Fig4B_2h_aTubulin_for_IKBa_sEVs_2.tif]

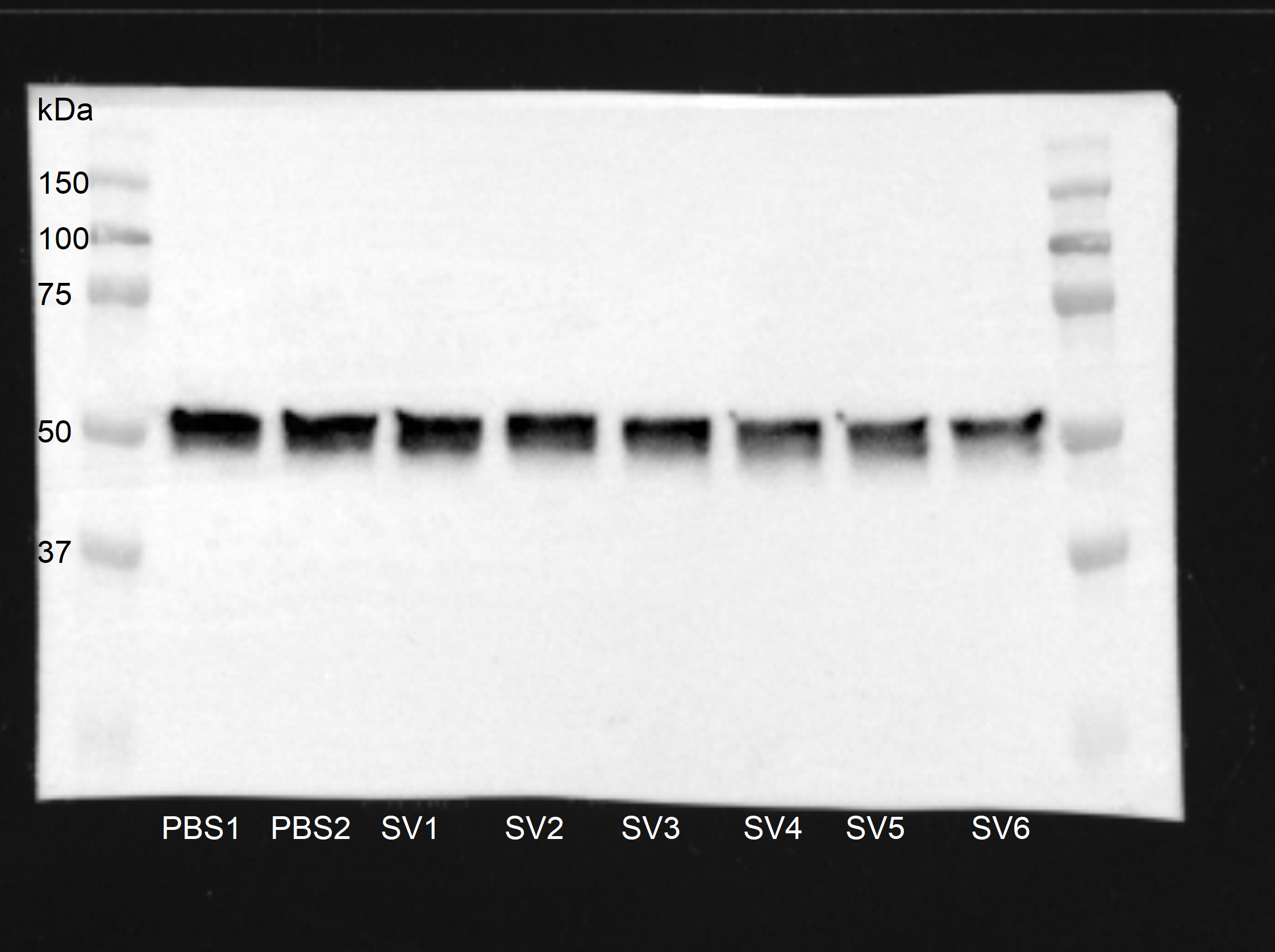

Supplement: Supplementary file 1 [file cancers-18-02219-s001.zip › supplement_proteomics_WB/full_WB_images_and_data/Fig4B_2h_aTubulin_for_IKBa_SV_2.tif]

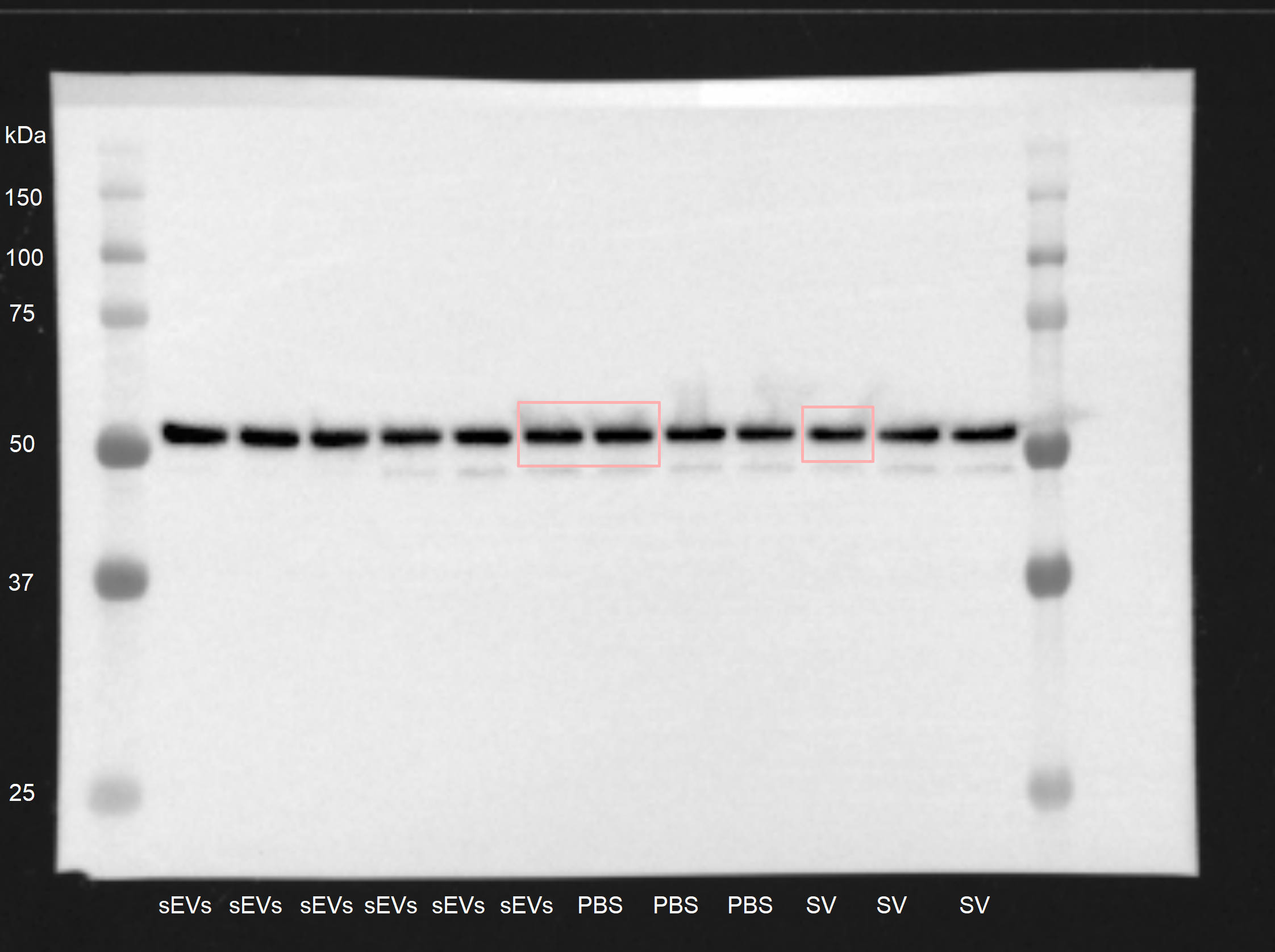

Supplement: Supplementary file 1 [file cancers-18-02219-s001.zip › supplement_proteomics_WB/full_WB_images_and_data/Fig4B_2h_aTubulin_for_pIKBa_1.tif]

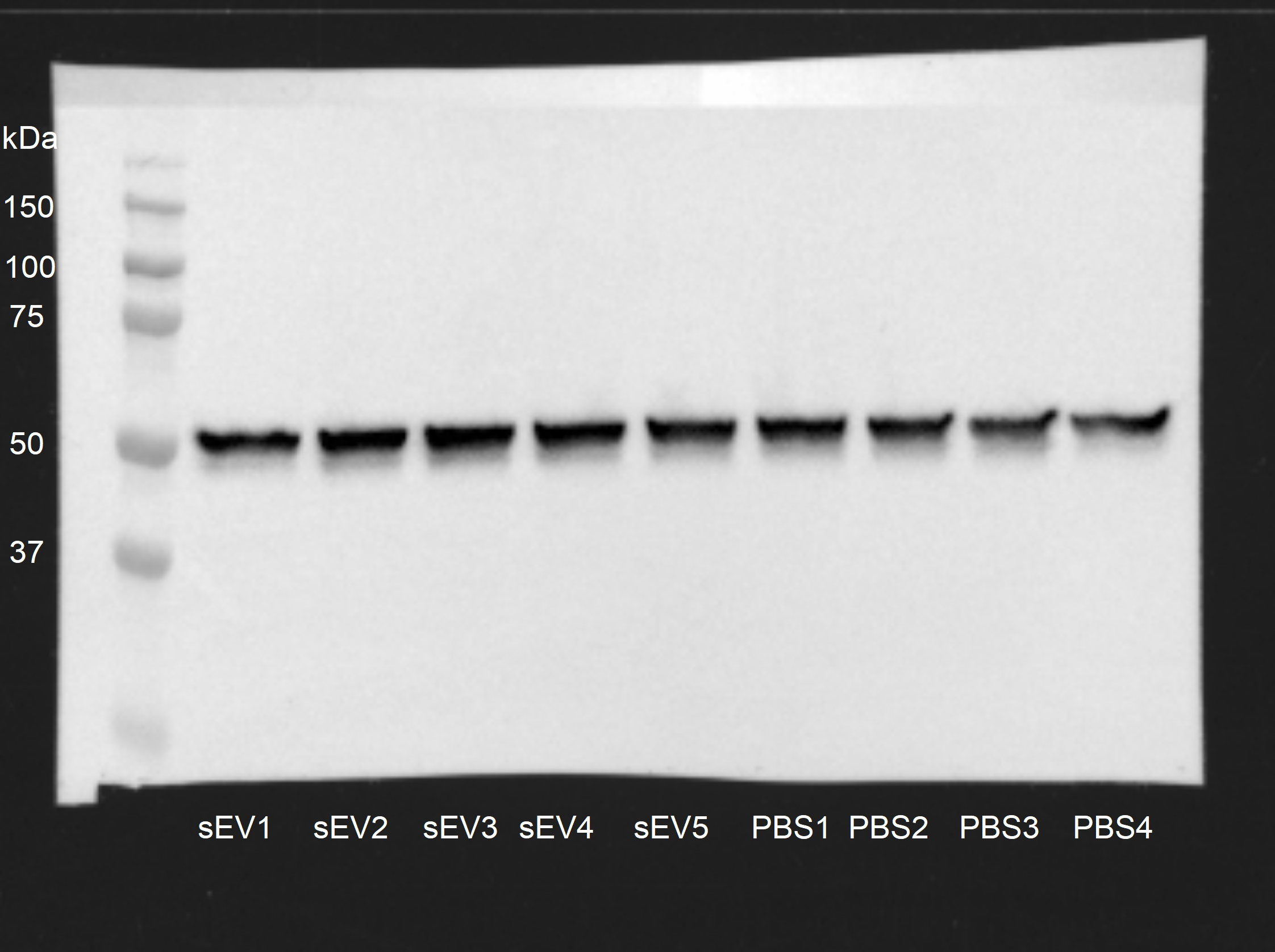

Supplement: Supplementary file 1 [file cancers-18-02219-s001.zip › supplement_proteomics_WB/full_WB_images_and_data/Fig4B_2h_aTubulin_for_pIKBa_sEVs_2.tif]

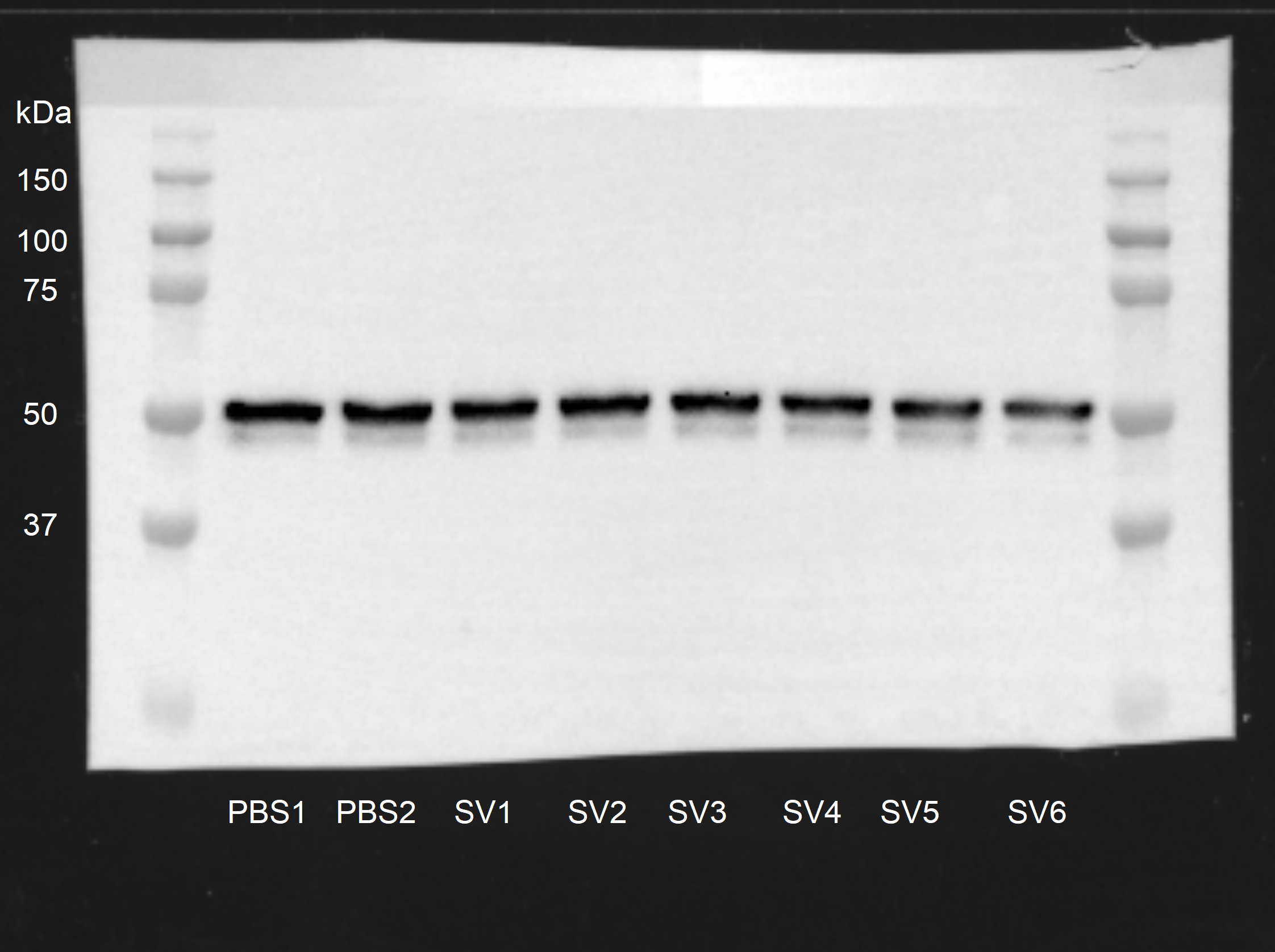

Supplement: Supplementary file 1 [file cancers-18-02219-s001.zip › supplement_proteomics_WB/full_WB_images_and_data/Fig4B_2h_aTubulin_for_pIKBa_SV_2.tif]

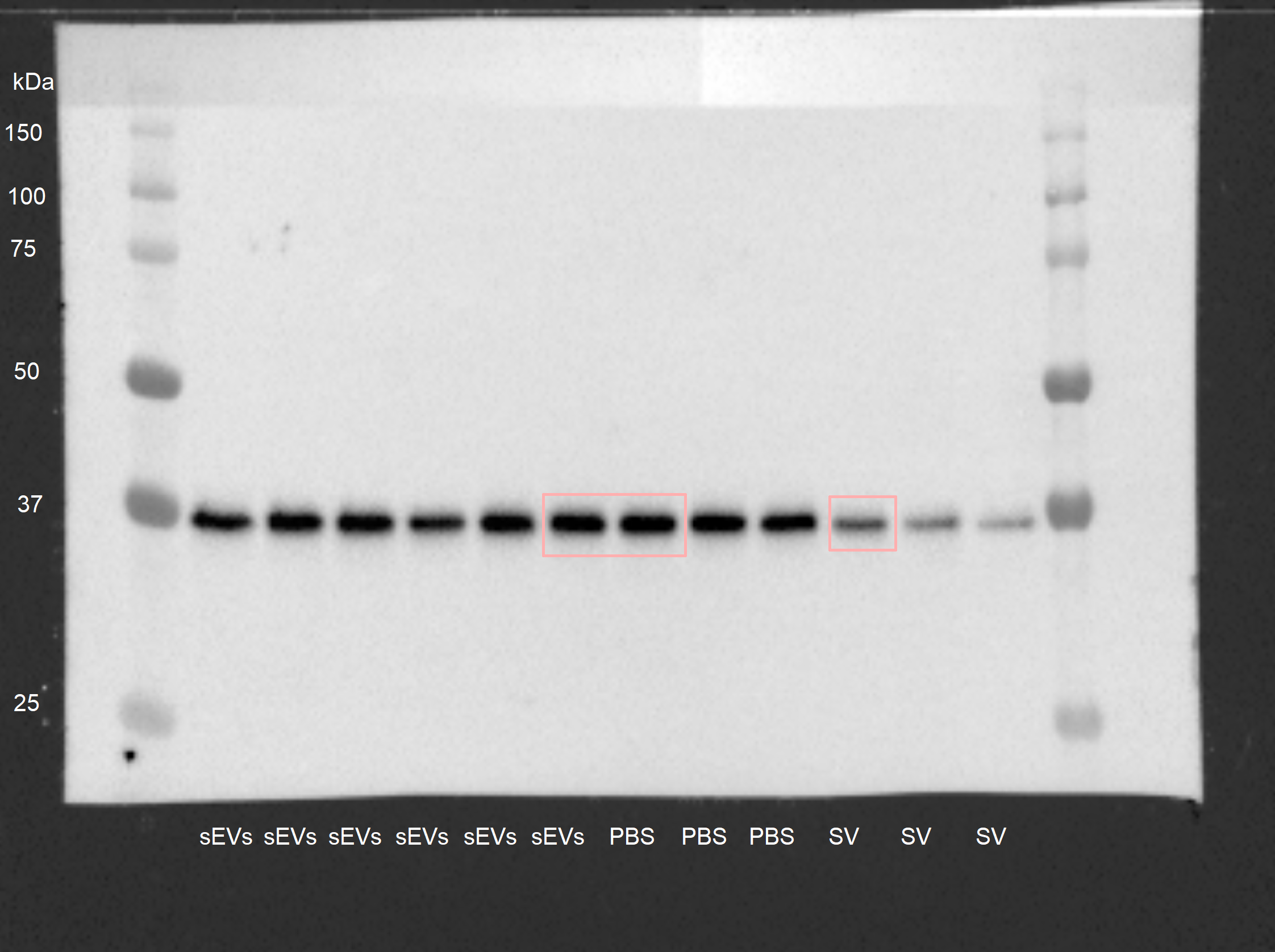

Supplement: Supplementary file 1 [file cancers-18-02219-s001.zip › supplement_proteomics_WB/full_WB_images_and_data/Fig4B_2h_IKBa_1.tif]

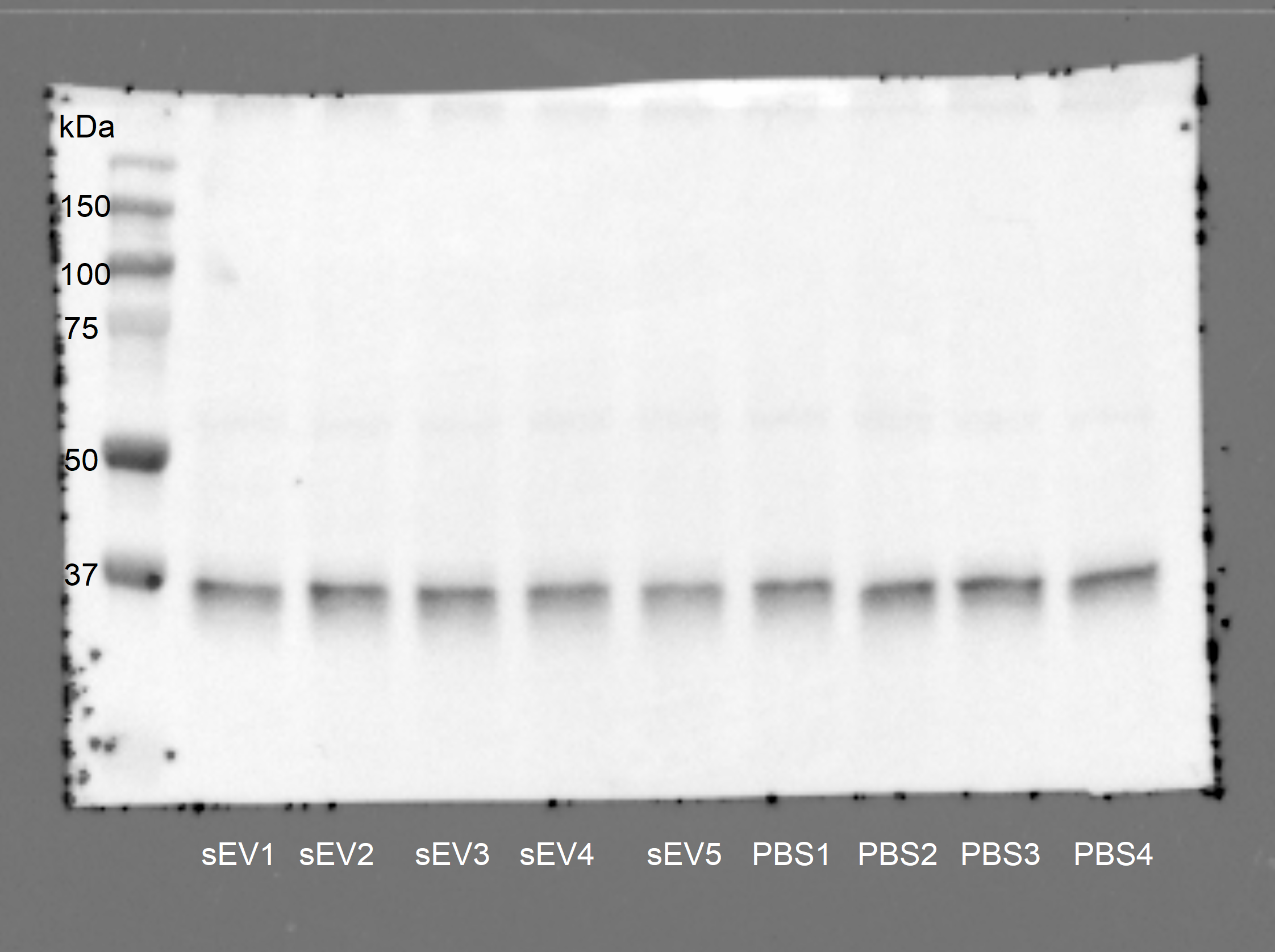

Supplement: Supplementary file 1 [file cancers-18-02219-s001.zip › supplement_proteomics_WB/full_WB_images_and_data/Fig4B_2h_IKBa_sEVs_2.tif]

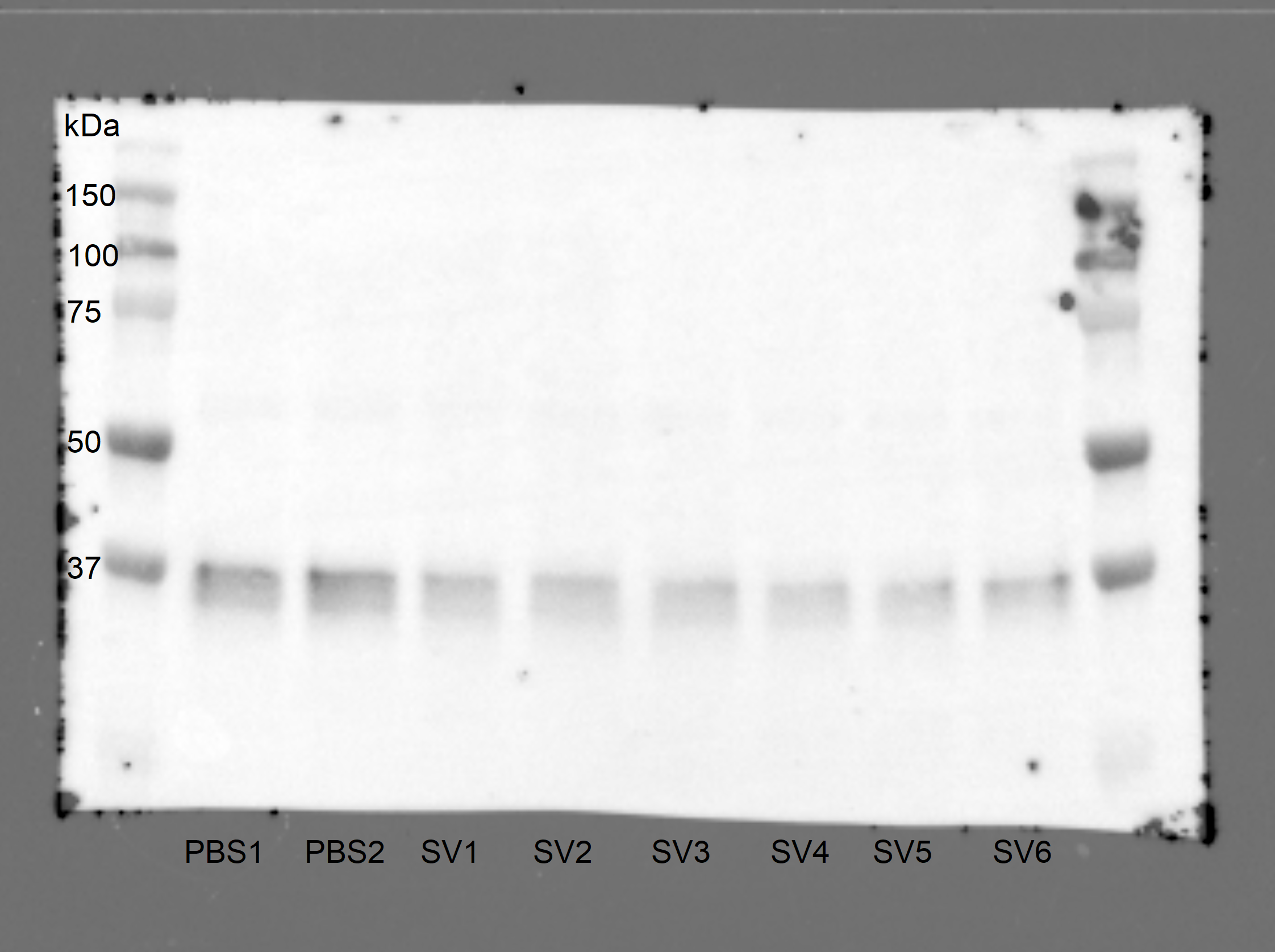

Supplement: Supplementary file 1 [file cancers-18-02219-s001.zip › supplement_proteomics_WB/full_WB_images_and_data/Fig4B_2h_IKBa_SV_2.tif]

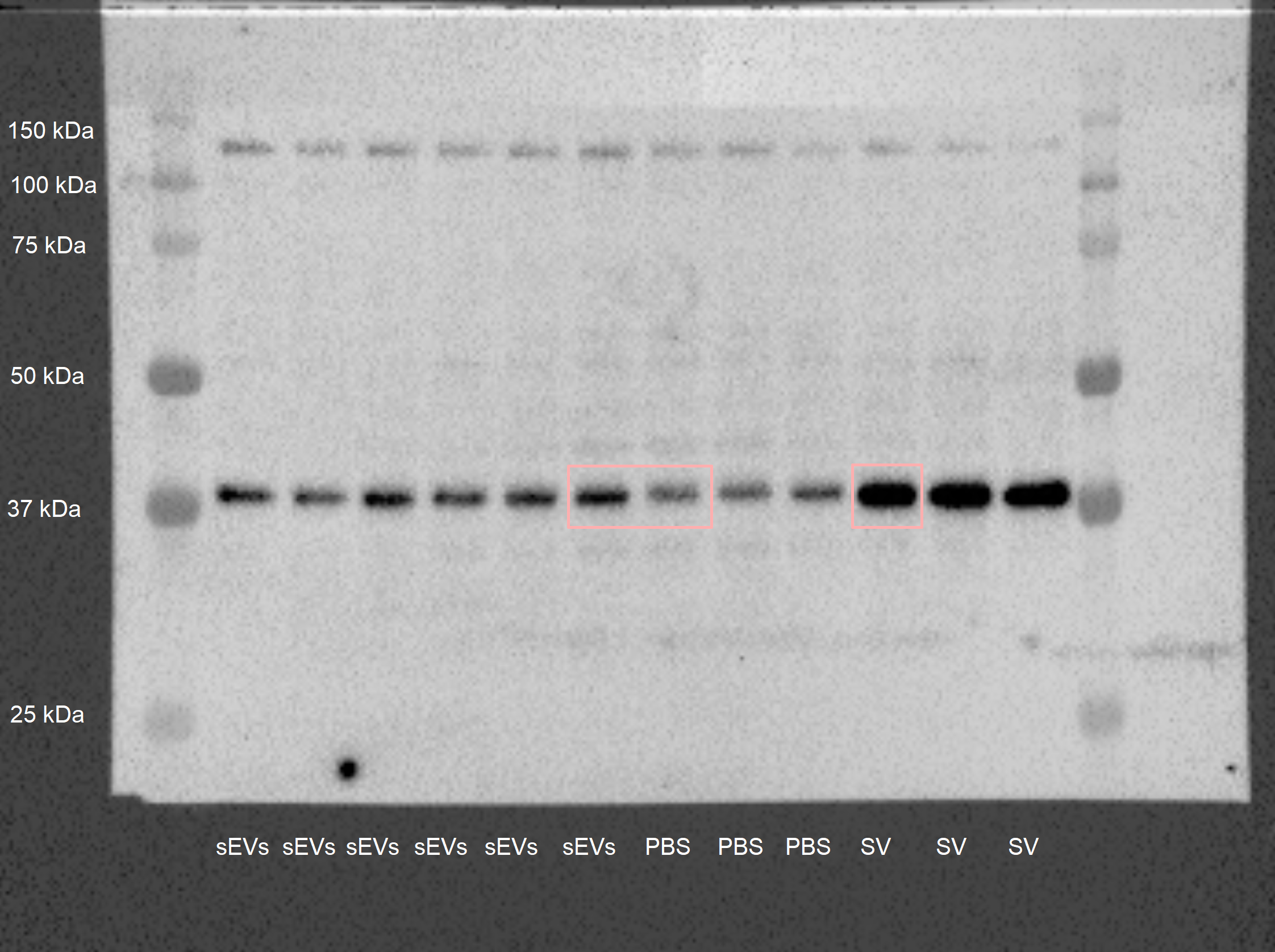

Supplement: Supplementary file 1 [file cancers-18-02219-s001.zip › supplement_proteomics_WB/full_WB_images_and_data/Fig4B_2h_pIKBa_1.tif]

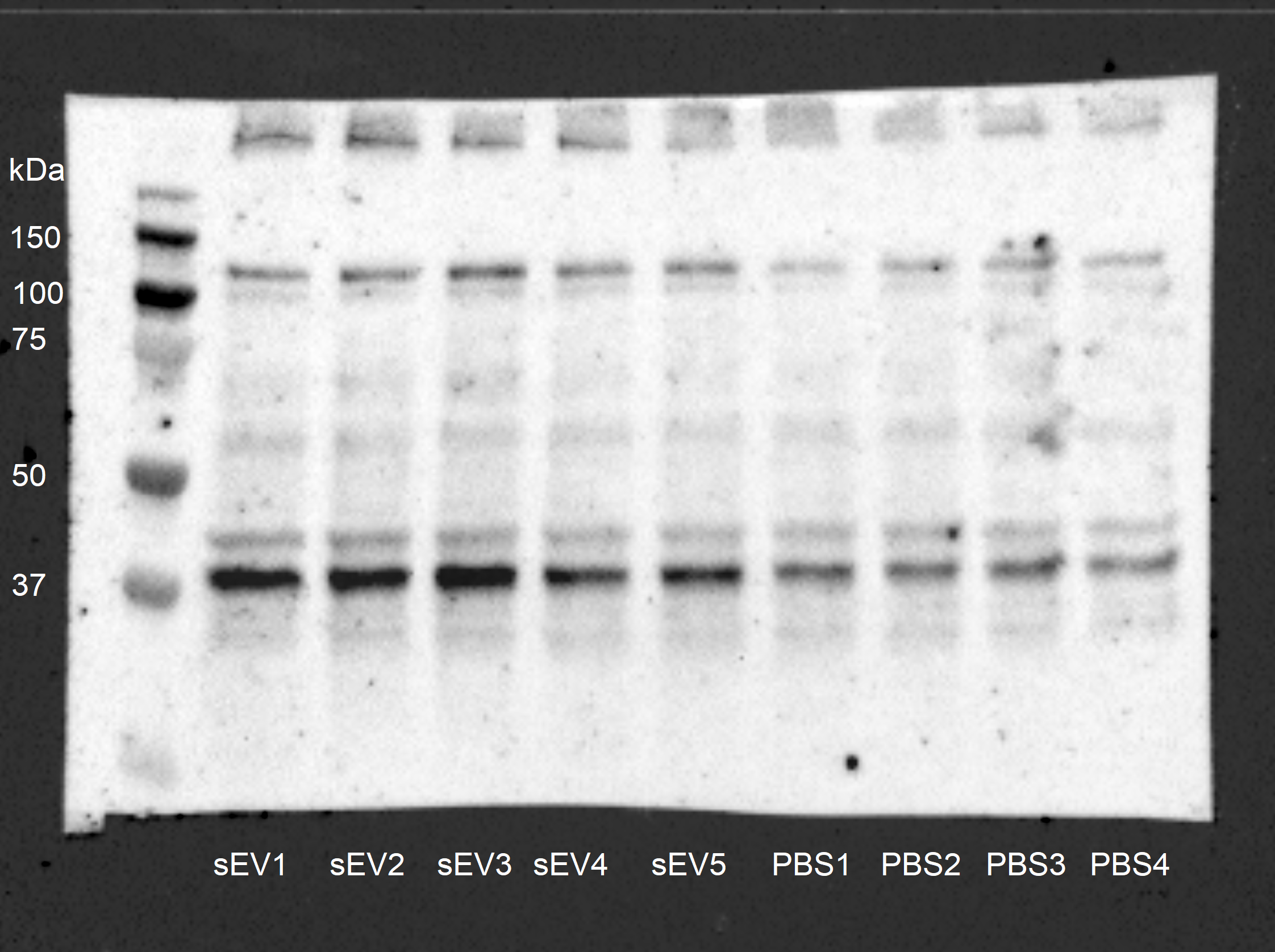

Supplement: Supplementary file 1 [file cancers-18-02219-s001.zip › supplement_proteomics_WB/full_WB_images_and_data/Fig4B_2h_pIKBa_sEV_2.tif]

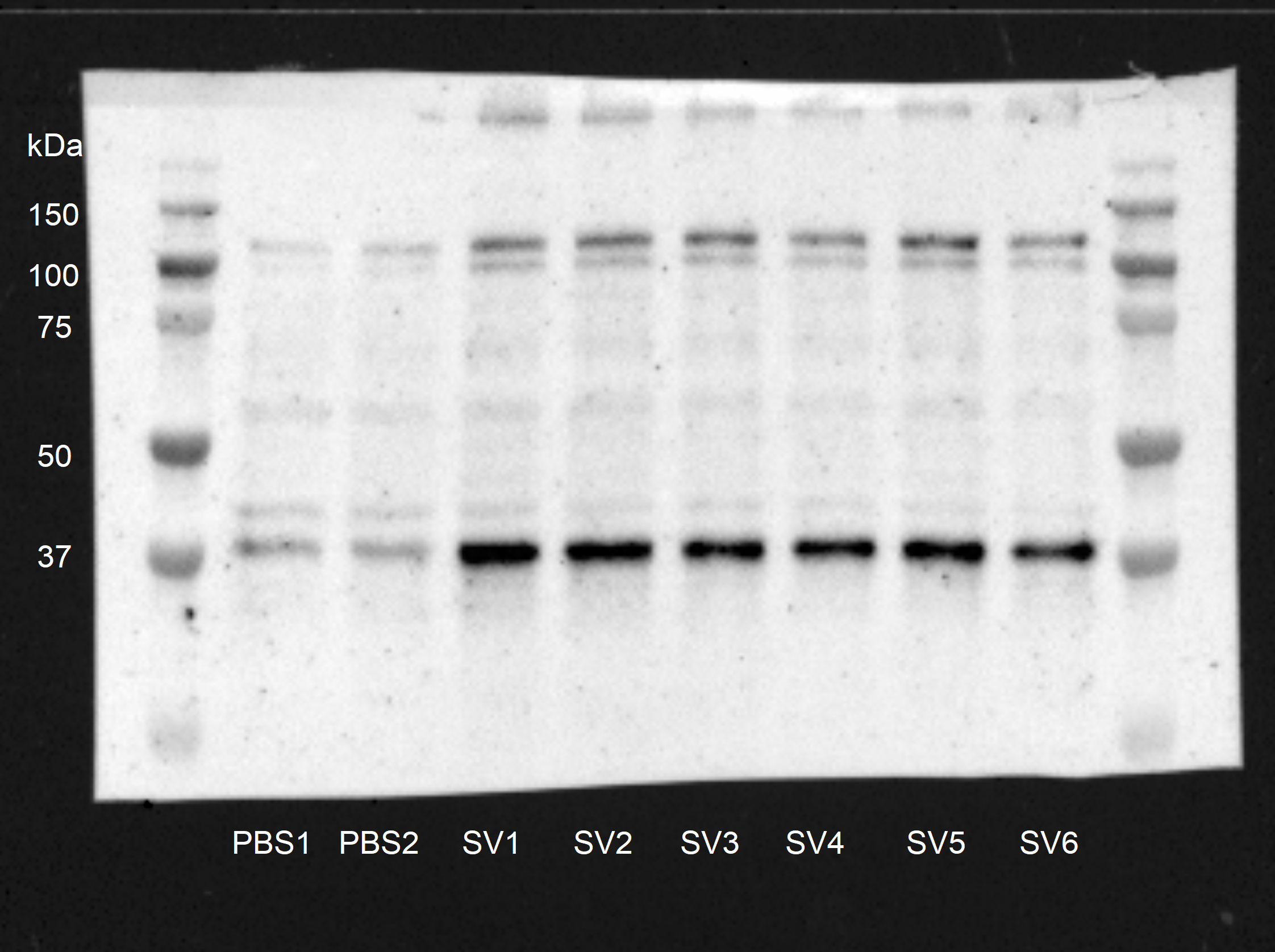

Supplement: Supplementary file 1 [file cancers-18-02219-s001.zip › supplement_proteomics_WB/full_WB_images_and_data/Fig4B_2h_pIKBa_SV_2.tif]

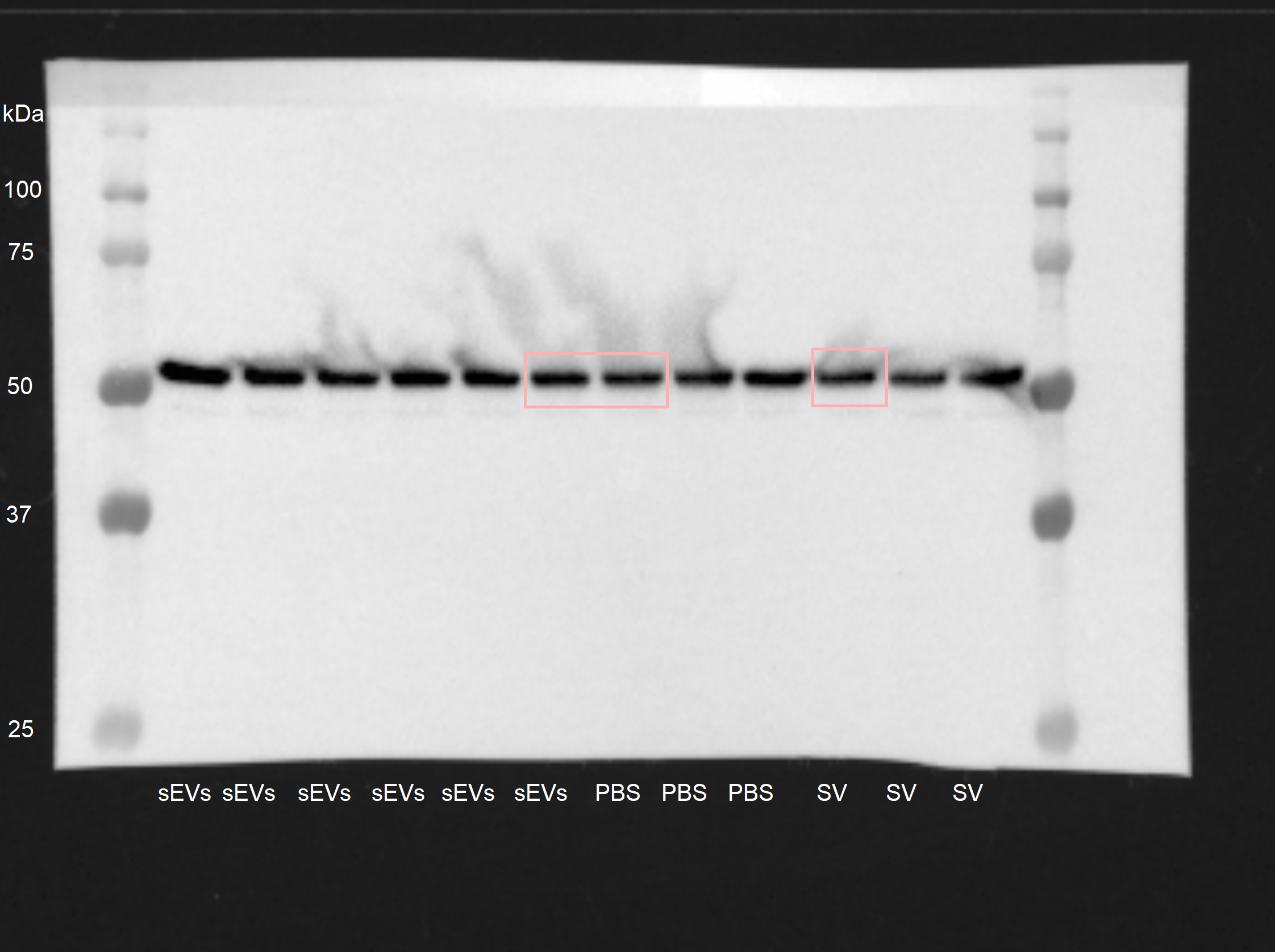

Supplement: Supplementary file 1 [file cancers-18-02219-s001.zip › supplement_proteomics_WB/full_WB_images_and_data/Fig4B_4h_aTubulin_for_IKBa_sEVs_PBS_SV_1.tif]

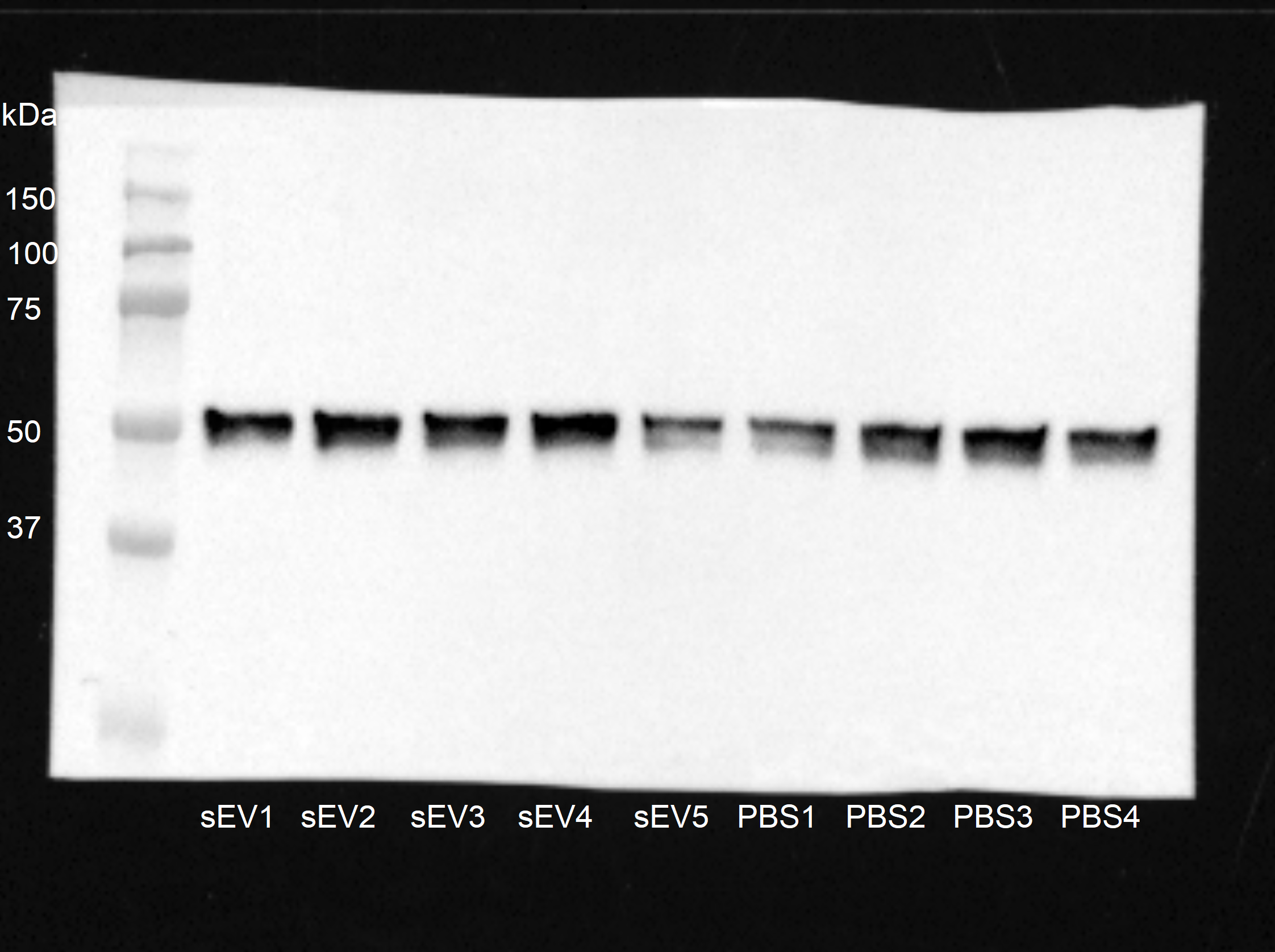

Supplement: Supplementary file 1 [file cancers-18-02219-s001.zip › supplement_proteomics_WB/full_WB_images_and_data/Fig4B_4h_aTubulin_for_IKBa_sEV_2.tif]

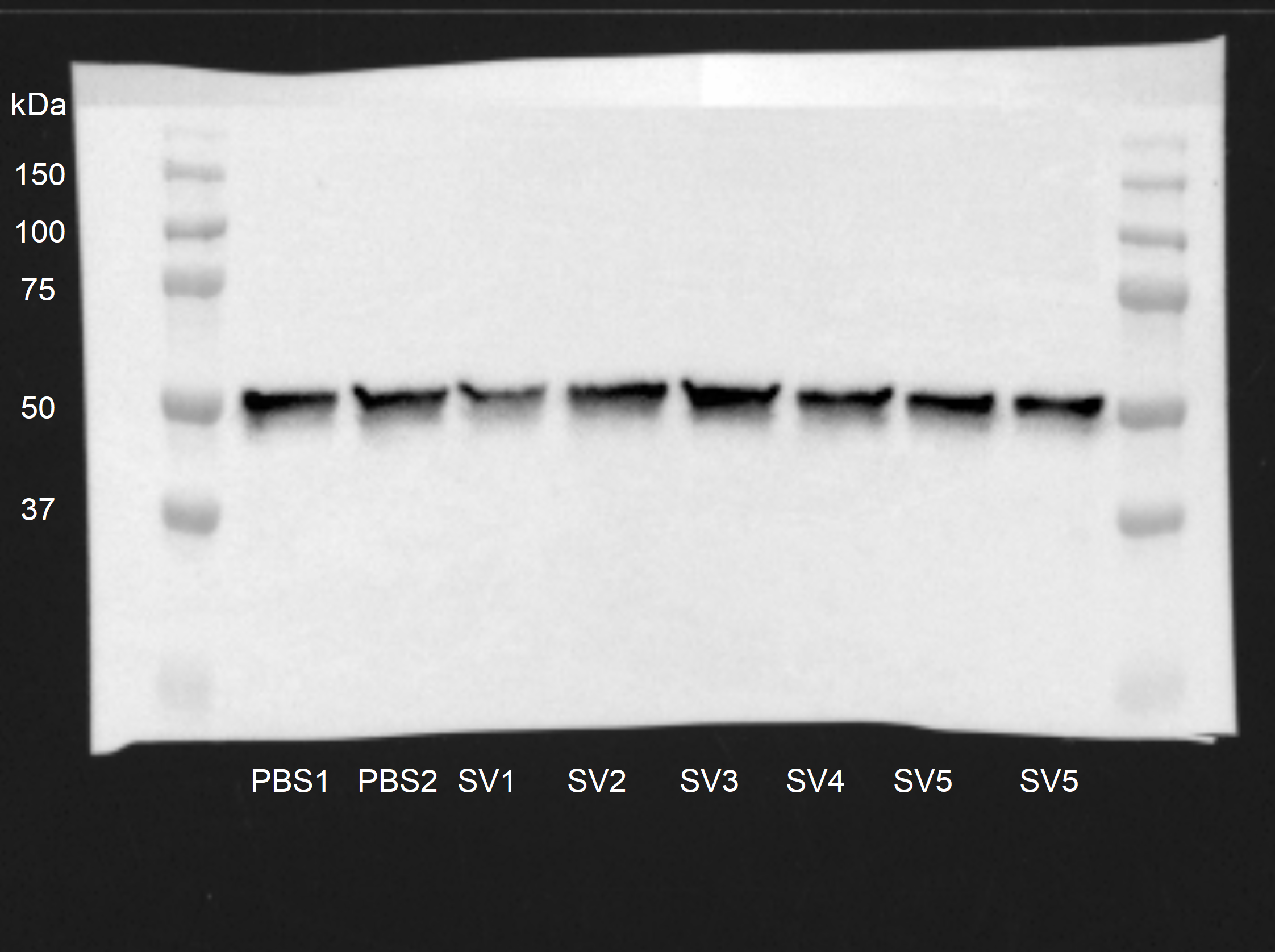

Supplement: Supplementary file 1 [file cancers-18-02219-s001.zip › supplement_proteomics_WB/full_WB_images_and_data/Fig4B_4h_aTubulin_for_IKBa_SV_2.tif]

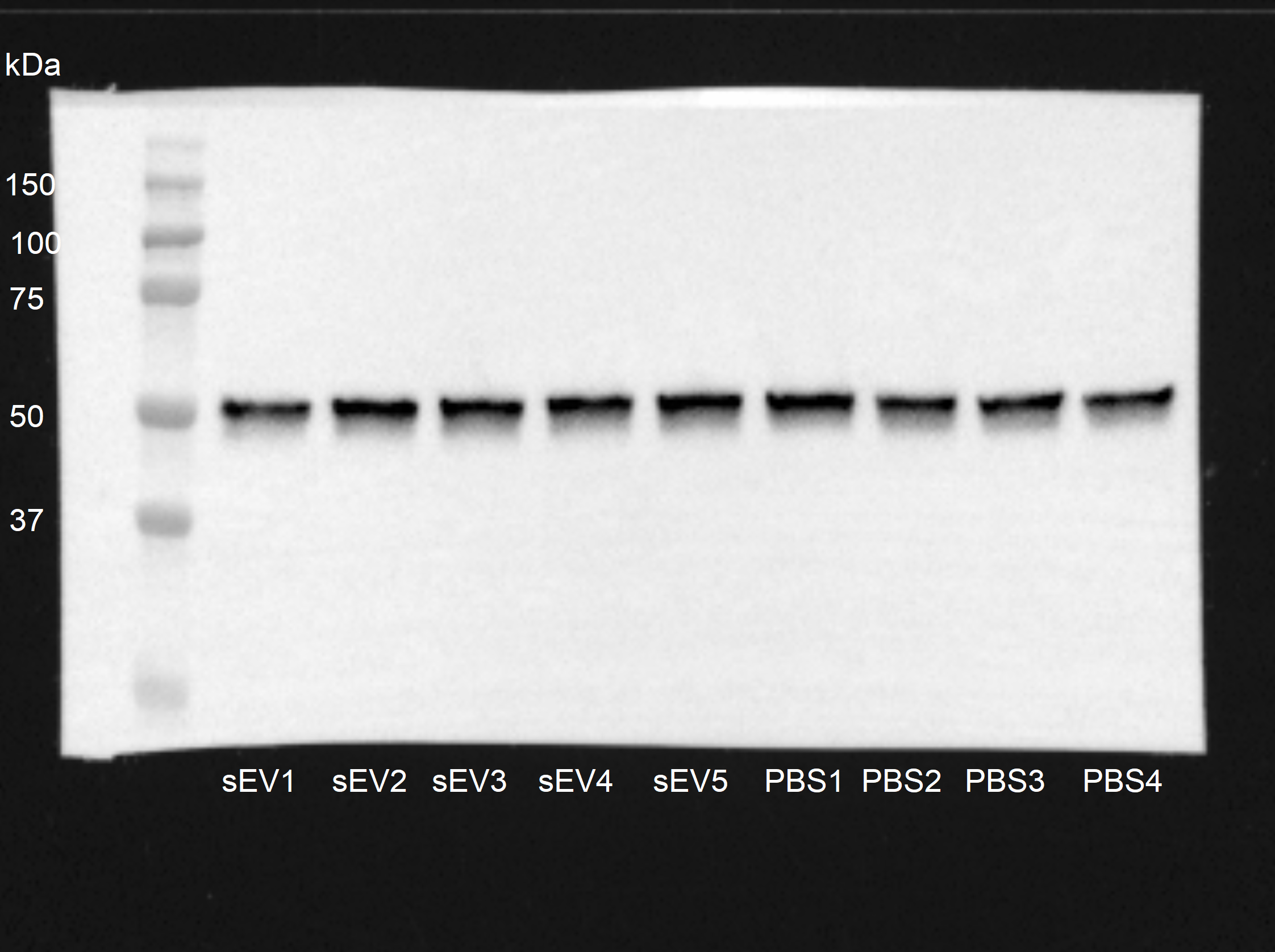

Supplement: Supplementary file 1 [file cancers-18-02219-s001.zip › supplement_proteomics_WB/full_WB_images_and_data/Fig4B_4h_aTubulin_for_pIKBa_sEVs_2.tif]

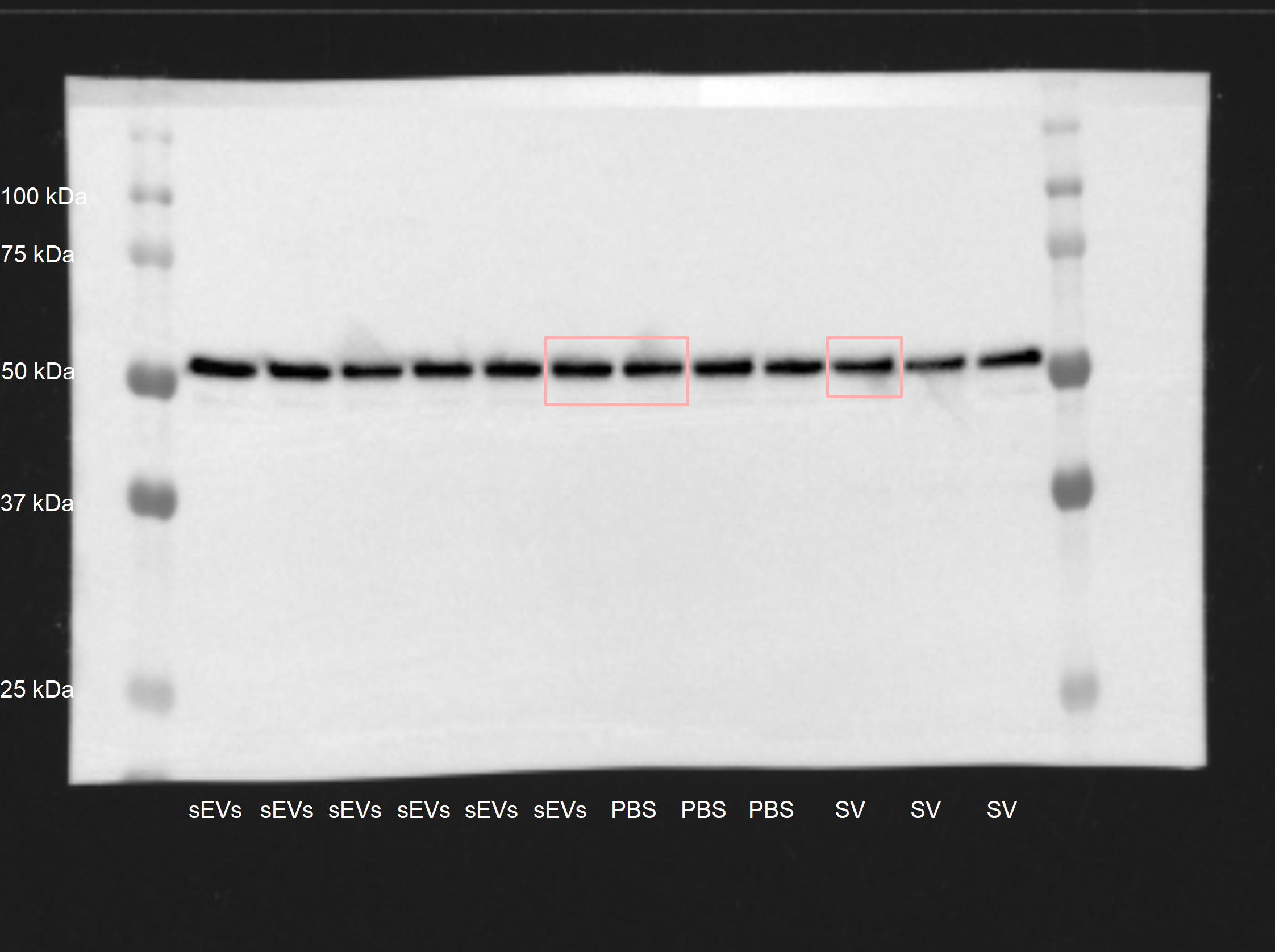

Supplement: Supplementary file 1 [file cancers-18-02219-s001.zip › supplement_proteomics_WB/full_WB_images_and_data/Fig4B_4h_aTubulin_for_pIKBa_sEVs_PBS_SV_1.tif]

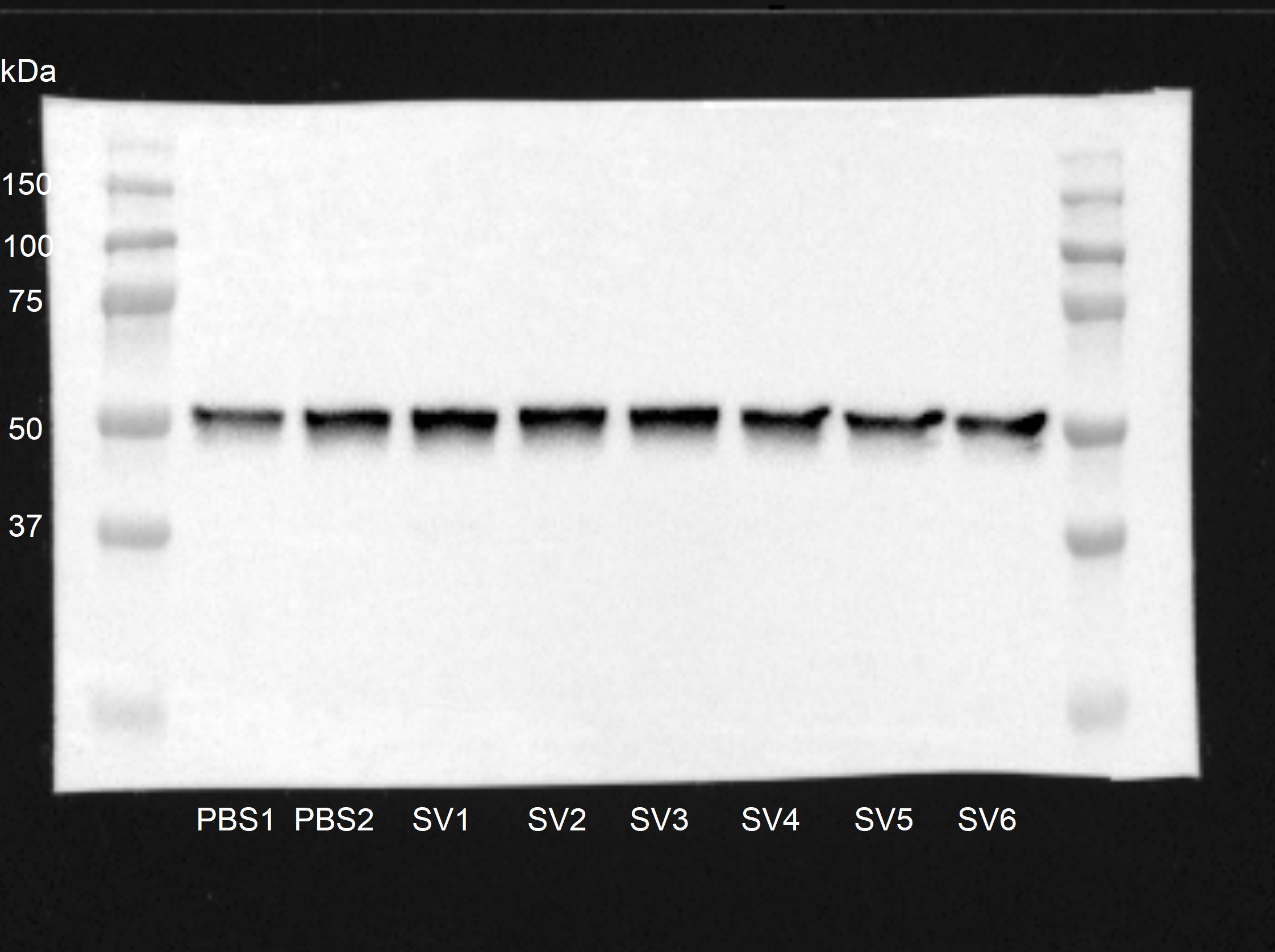

Supplement: Supplementary file 1 [file cancers-18-02219-s001.zip › supplement_proteomics_WB/full_WB_images_and_data/Fig4B_4h_aTubulin_for_pIKBa_SV_2.tif]

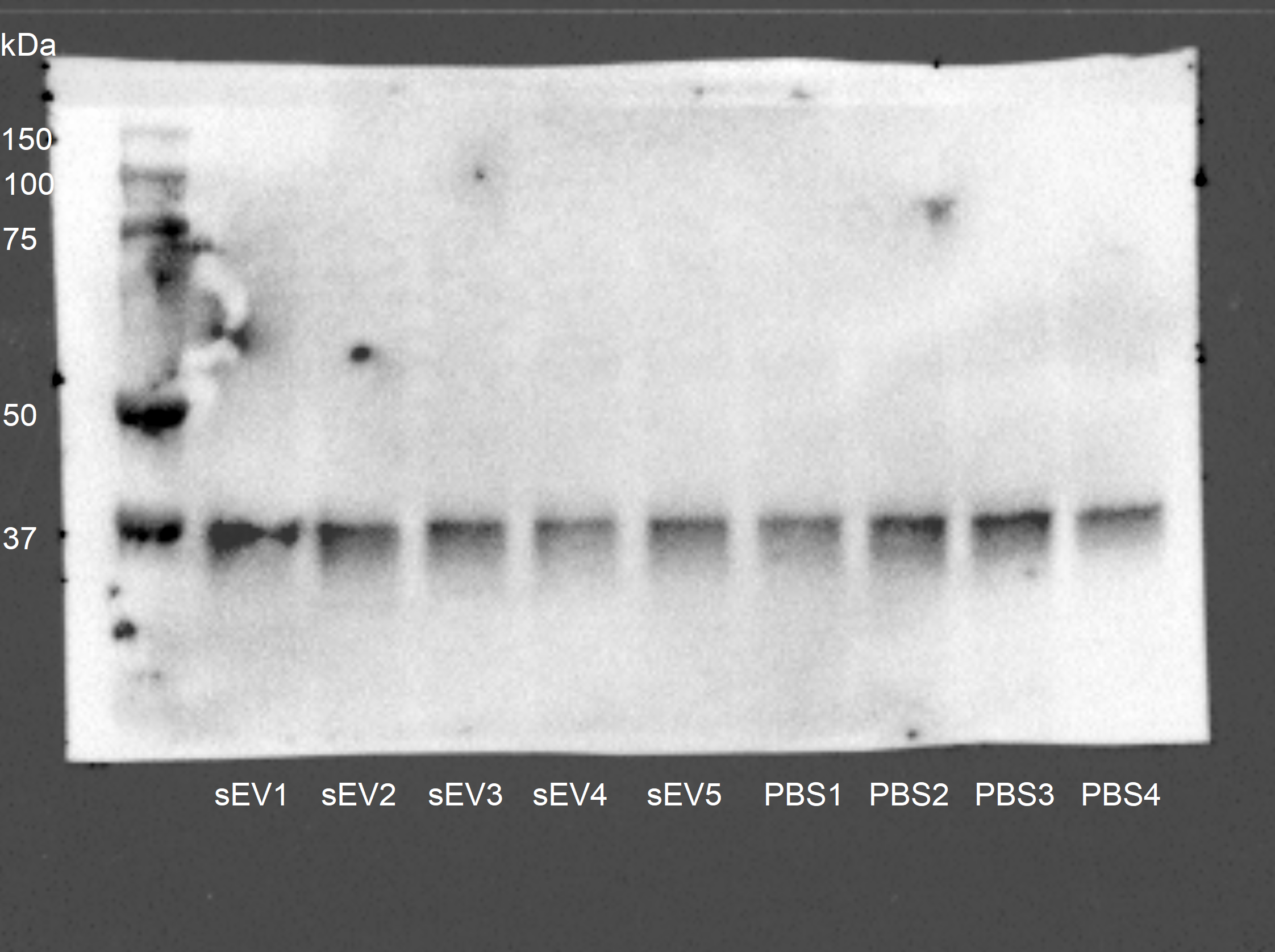

Supplement: Supplementary file 1 [file cancers-18-02219-s001.zip › supplement_proteomics_WB/full_WB_images_and_data/Fig4B_4h_IKBa_sEVs_2.tif]

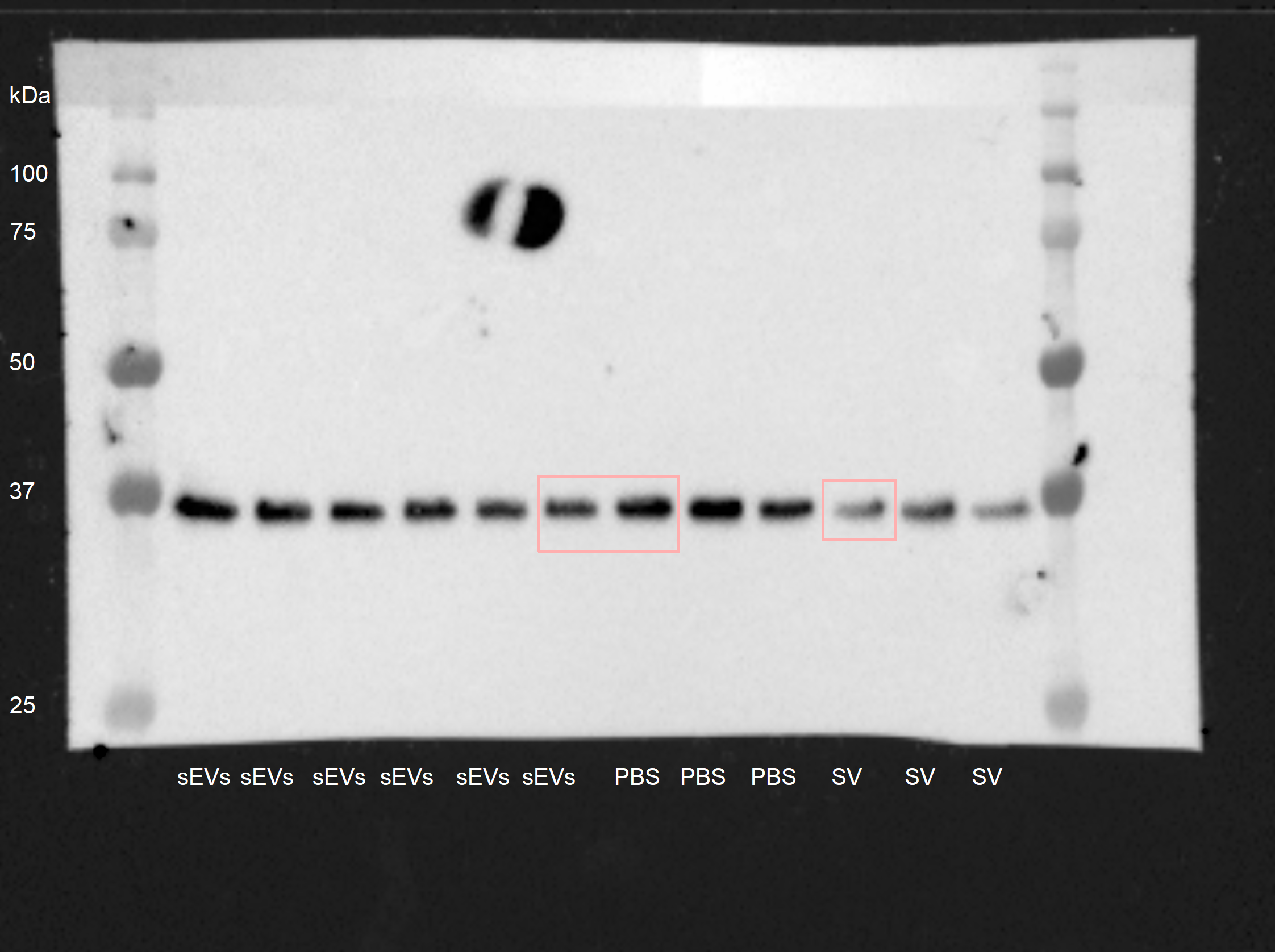

Supplement: Supplementary file 1 [file cancers-18-02219-s001.zip › supplement_proteomics_WB/full_WB_images_and_data/Fig4B_4h_IKBa_sEVs_PBS_SV_1.tif]

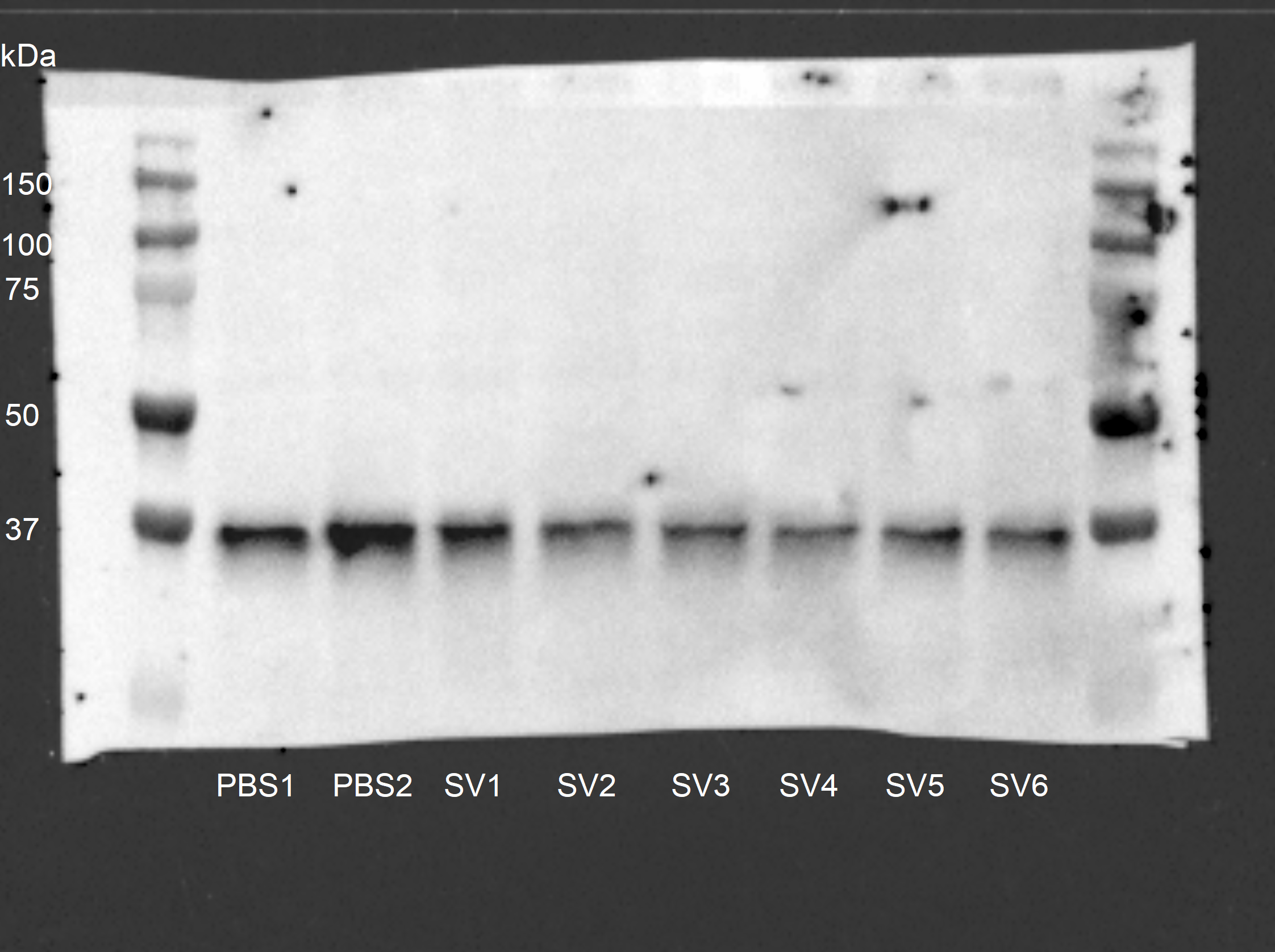

Supplement: Supplementary file 1 [file cancers-18-02219-s001.zip › supplement_proteomics_WB/full_WB_images_and_data/Fig4B_4h_IKBa_SV_2.tif]

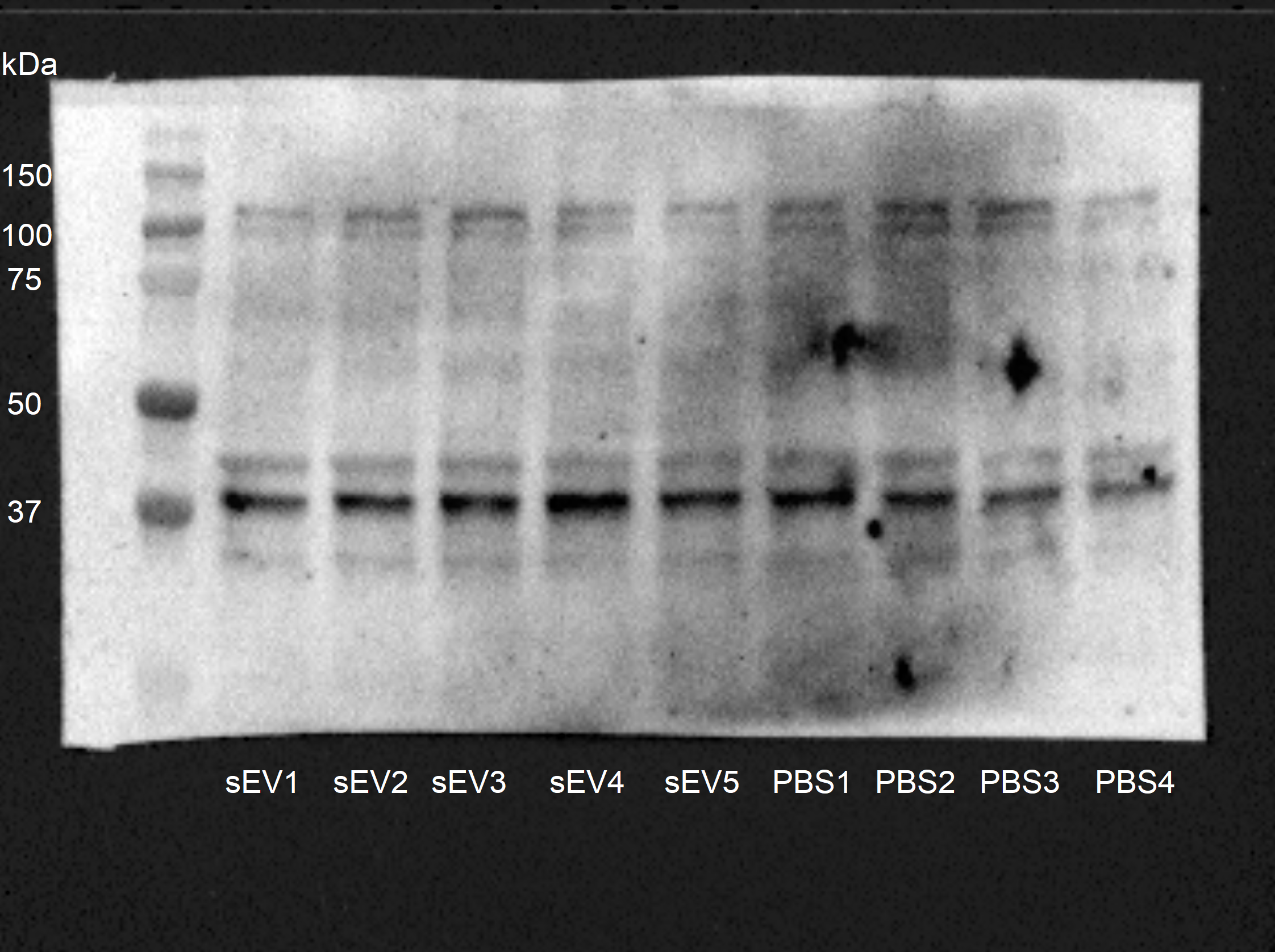

Supplement: Supplementary file 1 [file cancers-18-02219-s001.zip › supplement_proteomics_WB/full_WB_images_and_data/Fig4B_4h_pIKBa_sEVs_2.tif]

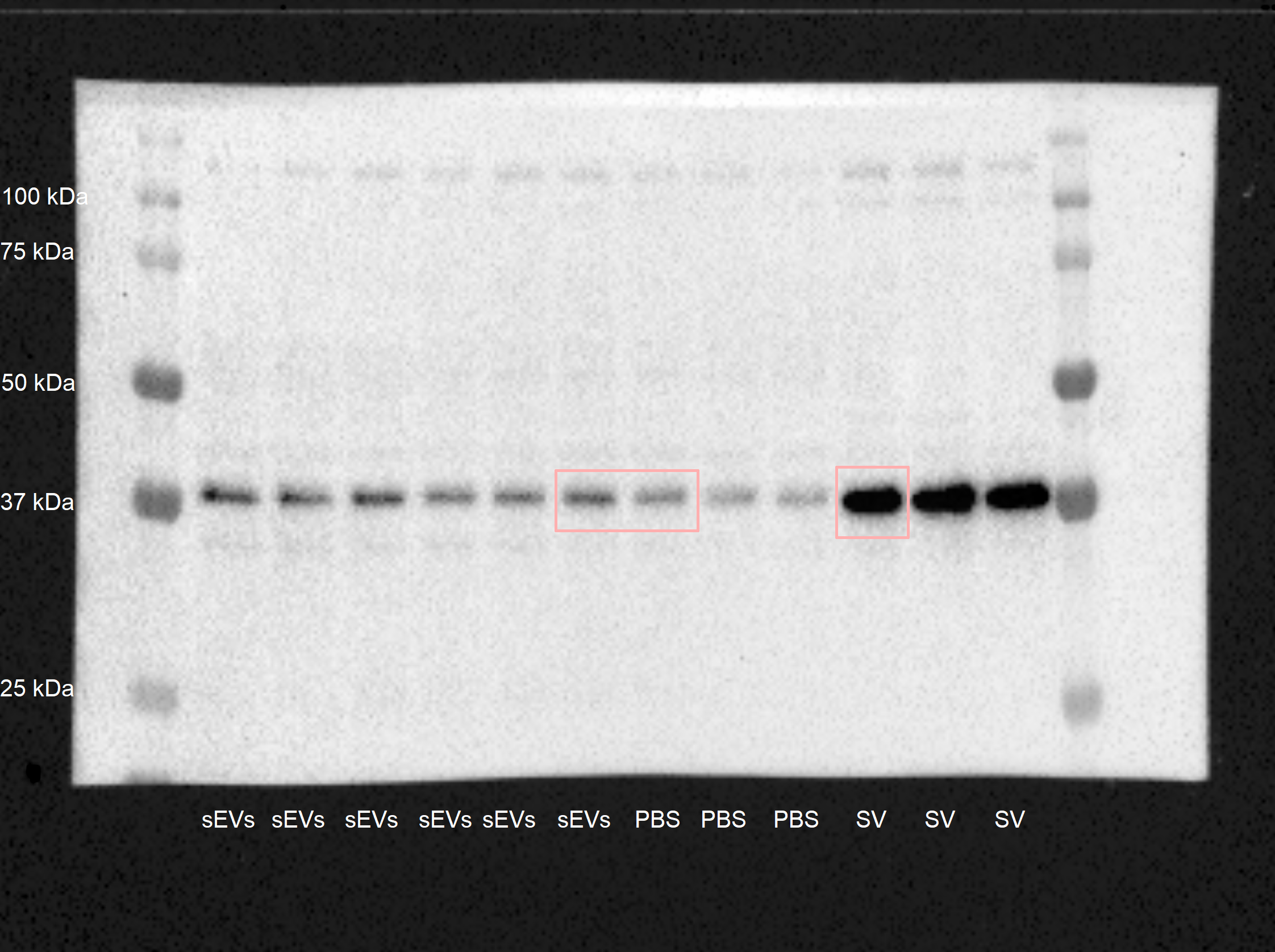

Supplement: Supplementary file 1 [file cancers-18-02219-s001.zip › supplement_proteomics_WB/full_WB_images_and_data/Fig4B_4h_pIKBa_sEVs_PBS_SV_1.tif]

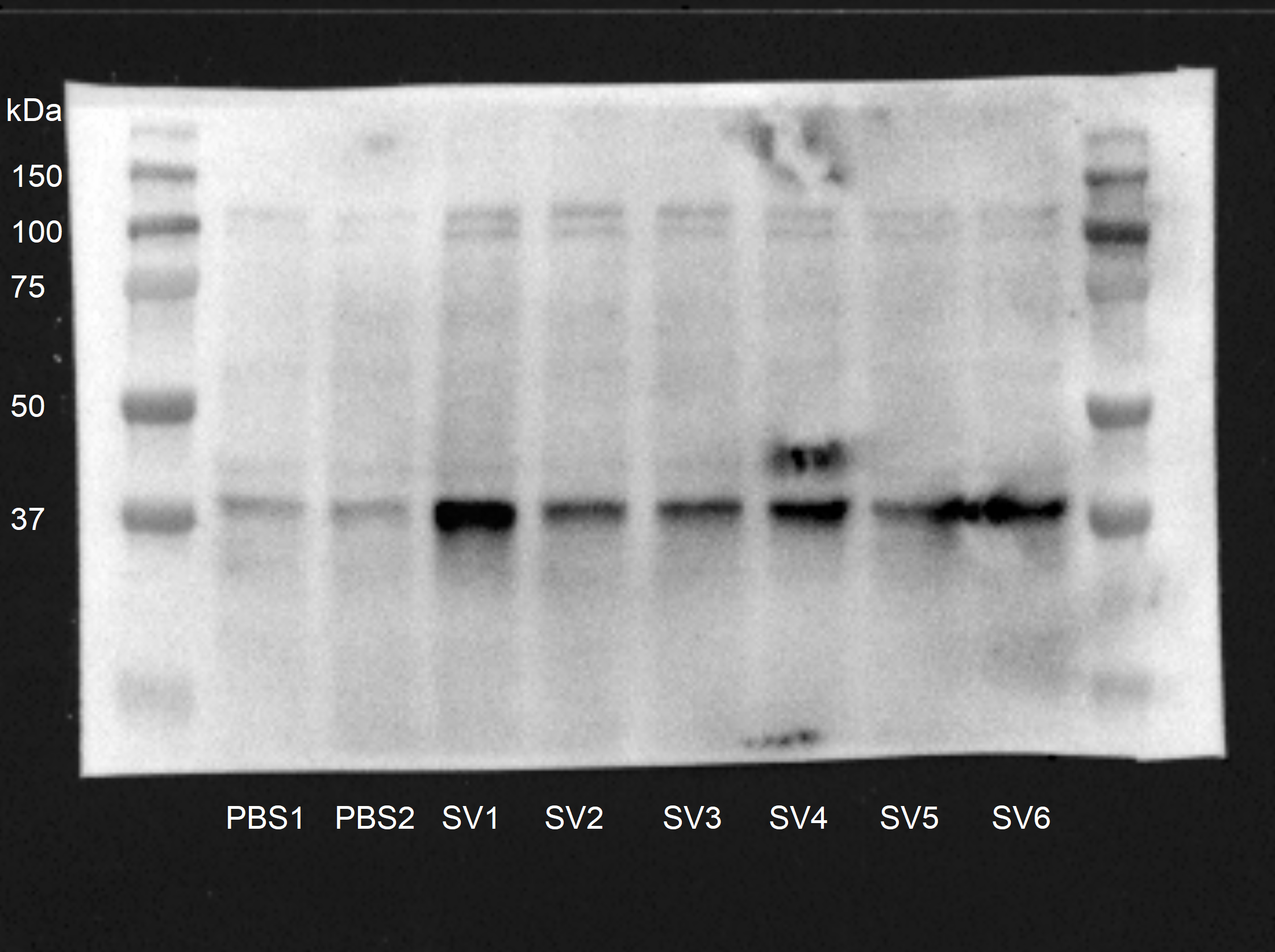

Supplement: Supplementary file 1 [file cancers-18-02219-s001.zip › supplement_proteomics_WB/full_WB_images_and_data/Fig4B_4h_pIKBa_SV_2.tif]

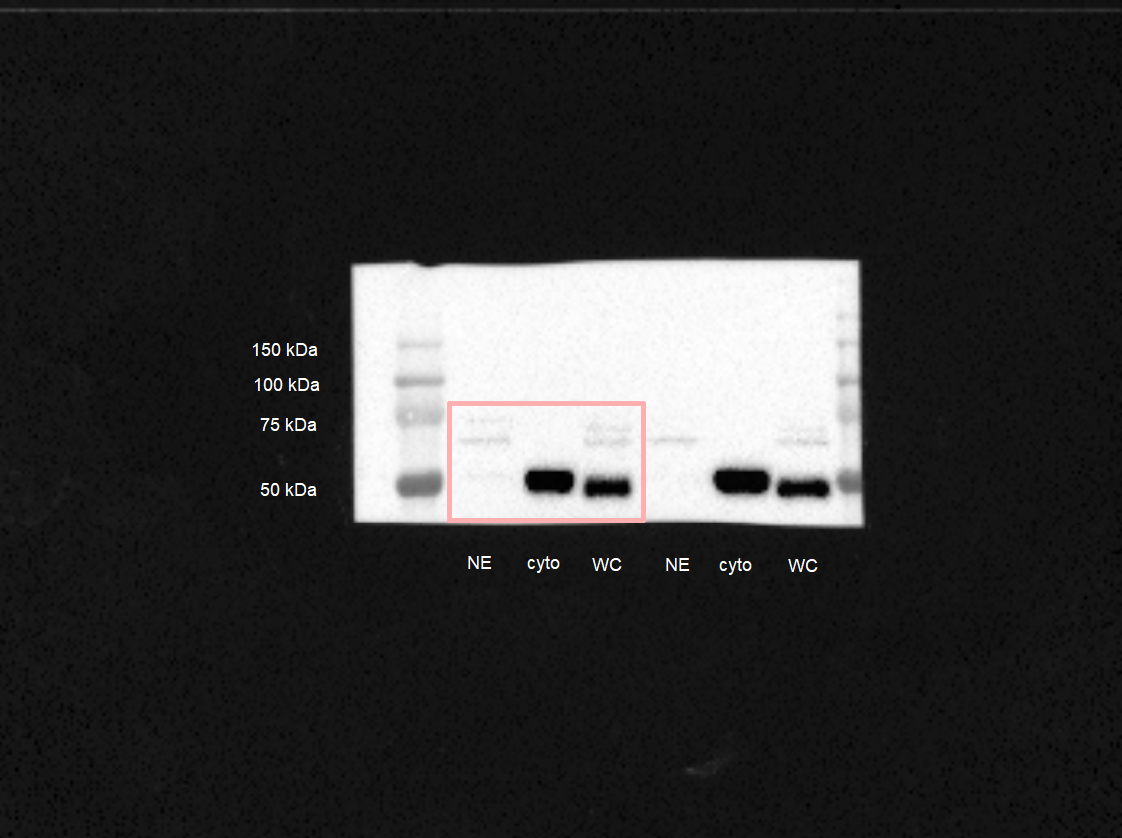

Supplement: Supplementary file 1 [file cancers-18-02219-s001.zip › supplement_proteomics_WB/full_WB_images_and_data/Fig4C_NE_Purity_aTub_marked.tif]

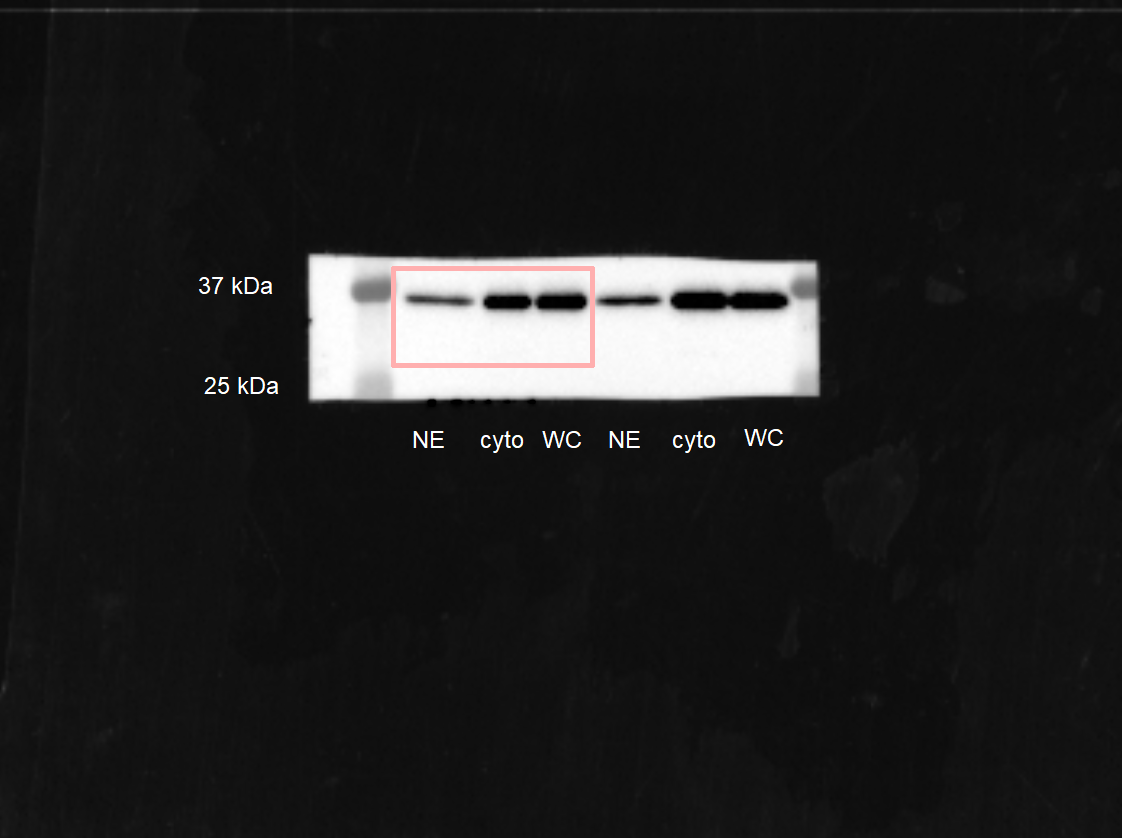

Supplement: Supplementary file 1 [file cancers-18-02219-s001.zip › supplement_proteomics_WB/full_WB_images_and_data/Fig4C_NE_Purity_GAPDH_marked.tif]

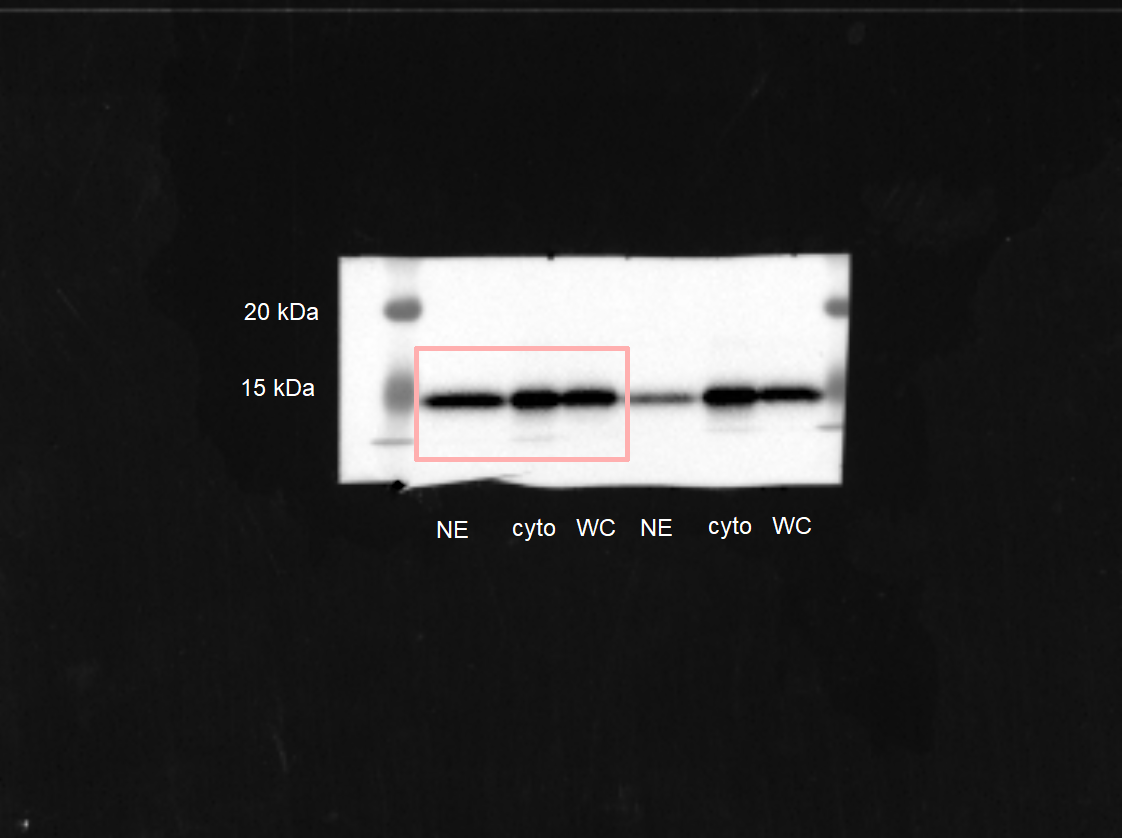

Supplement: Supplementary file 1 [file cancers-18-02219-s001.zip › supplement_proteomics_WB/full_WB_images_and_data/Fig4C_Purity_NE_CoxIV_marked.tif]

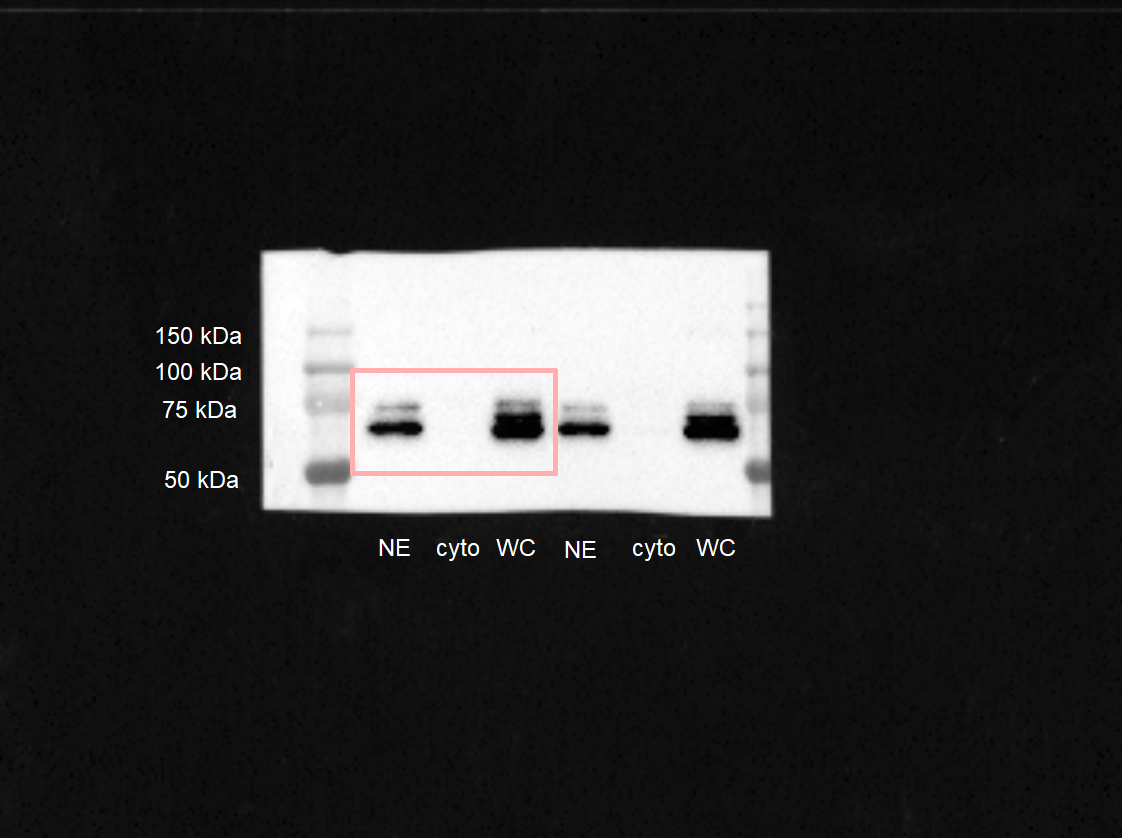

Supplement: Supplementary file 1 [file cancers-18-02219-s001.zip › supplement_proteomics_WB/full_WB_images_and_data/Fig4C_Purity_NE_LaminAC_marked.tif]
